# The harmonized activities of HER2–HER3 heterodimer and deacetylated FOXA1 evade hormone response by regulating FOXA1 chromatin binding

**DOI:** 10.1093/nar/gkaf1086

**Published:** 2025-11-13

**Authors:** Shixiong Wang, Gemma Santacana-Font, Darek Kedra, Siv Gilfillan, David Tena-Chaves, Helga Bergholtz, Olav Engebraaten, Ole Christian Lingjaerde, Javier Gutiérrez-Fernández, Jens Henrik Norum, Therese Sørlie, Sandra López-Aviles, Antoni Hurtado

**Affiliations:** Cell cycle regulation group, Institute of Biosciences (IBV), University of Oslo, Kristine Bonnevies hus, Blindernveien 31, 0371 Oslo, Norway; Functional Cancer Genomics group, Centro de Investigación del Cáncer and Instituto de Biología Molecular y Celular del Cáncer, Consejo Superior de Investigaciones Científicas and Universidad de Salamanca, Salamanca, 37007 Salamanca, Spain; Functional Cancer Genomics group, Centro de Investigación del Cáncer and Instituto de Biología Molecular y Celular del Cáncer, Consejo Superior de Investigaciones Científicas and Universidad de Salamanca, Salamanca, 37007 Salamanca, Spain; Cell cycle regulation group, Institute of Biosciences (IBV), University of Oslo, Kristine Bonnevies hus, Blindernveien 31, 0371 Oslo, Norway; Functional Cancer Genomics group, Centro de Investigación del Cáncer and Instituto de Biología Molecular y Celular del Cáncer, Consejo Superior de Investigaciones Científicas and Universidad de Salamanca, Salamanca, 37007 Salamanca, Spain; Department of Cancer Genetics, Institute for Cancer Research, The Norwegian Radium Hospital, N-0310 Oslo, Norway; Department of Oncology, Oslo University Hospital, and Department of Tumor Biology, Institute for Cancer Research, University of Oslo, N-0310 Oslo, Norway; Department of Cancer Genetics, Institute for Cancer Research, The Norwegian Radium Hospital, N-0310 Oslo, Norway; Department of Computer Science, University of Oslo, P.O. 1137 Blindern, 0318 Oslo, Norway; Institute of Biosciences (IBV), University of Oslo, Kristine Bonnevies hus, Blindernveien 31, 0371 Oslo, Norway; Department of Cancer Genetics, Institute for Cancer Research, The Norwegian Radium Hospital, N-0310 Oslo, Norway; Department of Cancer Genetics, Institute for Cancer Research, The Norwegian Radium Hospital, N-0310 Oslo, Norway; Cell cycle regulation group, Institute of Biosciences (IBV), University of Oslo, Kristine Bonnevies hus, Blindernveien 31, 0371 Oslo, Norway; Functional Cancer Genomics group, Centro de Investigación del Cáncer and Instituto de Biología Molecular y Celular del Cáncer, Consejo Superior de Investigaciones Científicas and Universidad de Salamanca, Salamanca, 37007 Salamanca, Spain

## Abstract

FOXA1 is a key transcription factor that mediates the effects of estrogen receptor (ER) and HER2 signaling in breast cancer. However, the mechanisms underlying FOXA1 regulation by HER2 and ER remain poorly understood. Here, we investigated FOXA1 regulation in cells with varying HER2 levels and its impact on endocrine therapy response. Chromatin interaction analyses revealed that high HER2 levels enhance FOXA1 binding to chromatin regions while reducing ER occupancy. Mechanistically, FOXA1 is acetylated by the histone acetyltransferase EP300 at the WD1 domain in ER-positive cells, attenuating its DNA binding at HER2-induced chromatin regions. Conversely, FOXA1 deacetylation—triggered by HER2/HER3 activation—increases its binding to ER-independent regions and promotes insensitivity to hormone therapy. In a luminal breast cancer patient-derived xenograft model, HER2/HER3 signaling increased FOXA1 chromatin binding and reduced sensitivity to ER-targeted treatment. We identify HDAC2 as a key deacetylase modulating FOXA1 acetylation and partially mediating the effects of HER2/HER3 signaling. Altogether, our findings highlight the significance of FOXA1 acetylation, regulated by the HER2/HER3–HDAC2–FOXA1 axis, in controlling FOXA1 chromatin binding and shaping breast cancer progression and therapy response. These insights may inform future therapeutic strategies.

## Graphical Abstract

**Graphical Abstract:**
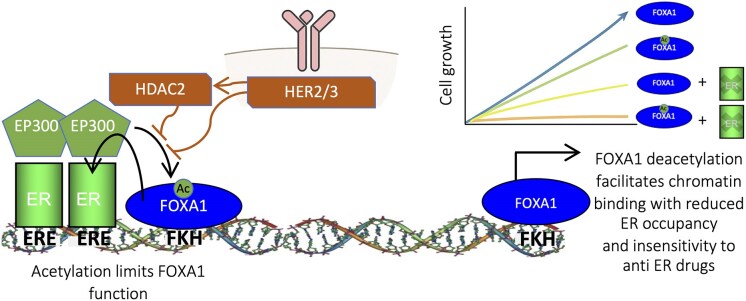

## Introduction

FOXA1 is a transcription factor belonging to the Forkhead family, and it plays a critical role in estrogen receptor (ER) signaling. It is essential for cell cycle progression and proliferation in luminal breast cancer. FOXA1 acts as a pioneer factor, facilitating the expression of most ER-regulated genes during the initiation and progression of luminal breast cancers [1]. Luminal subtypes are the most common types of breast cancer and are characterized by the expression of ER [2].

Estrogen signaling exerts its growth-promoting effects by inducing ER binding to various chromatin sites [3], leading to changes in the expression of coding and noncoding RNAs [4]. Hormone therapies are used to inhibit the function of ER, reduce tumor growth, and improve survival. ER expression serves as an important diagnostic and predictive marker in breast cancer [5]. Patients with ER-positive (ER+) and HER2-negative tumors exhibit the best responses to endocrine agents. However, dysregulated expression and function of FOXA1, caused by amplification, mutation, or upregulation, can drive resistance to endocrine and HER2-targeted therapies, resulting in worse patient outcomes [6]. In fact, resistance to these treatments has become a significant clinical challenge. Clinical studies have shown that ∼10%–15% of patients with early stage ER+ breast cancer experience recurrence on adjuvant endocrine therapy within 5 years [7], and the recurrence reaches rates as high as 30% by 15 years [8]. Several mechanisms of resistance to endocrine therapies have been identified, often involving enhanced growth factor signaling, mutations in ER or FOXA1, and changes in the expression or action of ER. Notably, HER2 activation is a frequently observed event in growth factor signaling, explaining resistance to endocrine treatment. Preclinical data strongly support HER2 signaling as a mechanism underlying endocrine treatment resistance in ER+ breast cancer [9, 10].

HER2 is a member of the HER family of mitogenic receptors, which includes ERBB1/HER1/EGFR, ERBB2/HER2, ERBB3/HER3, and ERBB4/HER4. Both amplification and mutation of HER2 have been causally linked to endocrine treatment resistance. While preclinical studies show that treating patients with such tumors using HER2 or pan-HER inhibitors significantly improves survival, clinical outcomes have not consistently mirrored these preclinical findings [11]. Furthermore, in most cases of ER+ tumors that are HER2-low and lack HER2 amplification or mutation, treatment with conventional HER inhibitors has shown some clinical benefit [12]. However, the molecular mechanisms through which HER2 regulates FOXA1 in ER+/HER2-low breast cancer require further investigation. In this study, we aimed to explore the differential regulation of FOXA1 in cells with varying levels of HER2 and its implications in response to endocrine treatment. Our findings reveal that the HER2–HER3 heterodimer is a key driver of tumor growth in endocrine-resistant patients with ER+/HER2-low tumors. Additionally, we provide mechanistic data explaining the differential regulation of FOXA1 by HER2 and ER in breast cancer cells.

## Materials and methods

### Cell culture and drug treatment

Cell lines were obtained from American Type Culture Collection (ATCC, Manassas, VA). BT474, MCF-7, and MDA-MB-453 cell lines were cultured in Dulbecco’s modified Eagle’s medium (DMEM) (4.5 g/l glucose). MCF-7–HER2 cell line was kindly shared by Prof. Maurizio Scaltriti (AstraZeneca, USA). MCF-7 cells expressing FOXA1-WT, FOXA1-WD1R, FOXA1-WD2R, and FOXA1-WD12R stably transfected cell lines were created for this work in our laboratory.

Cells were grown in DMEM and treated with growth factors—heregulin at 25 ng/ml and EGF at 100 ng/ml—and/or anti-HER2 drugs (trastuzumab, 20 ng/ml) for 60 min.

### Plasmids

HA-tagged FOXA1 was subcloned into pCI-neo (E1841, Promega, Madison, WI, USA) expression vector for transient transfection. Two lysines in Wing 1 (K237 and 240) and three lysines in Wing 2 (264, 267, and 270) were mutated into arginine (WD1R and WD2R) or all mutated (WD12R). The mutagenesis was carried out with QuikChange Lightning Site-Directed Mutagenesis Kit (Agilent, Santa Clara, CA, USA).

### Chromatin Immunoprecipitation

FOXA1 genomic regions were identified by using the cross-linking (X)-chromatin immunoprecipitation (ChIP) protocol as described previously [13]. Chromatin was incubated with ChIP-grade FOXA1 antibodies (5 mg of antibodies Abcam ab5089 and ab23738) and Protein A/G agarose beads (Life Technologies). Library preparation for sequencing was done following the instructions of TruSeq DNA Sample Preparation Kit from Illumina or MicroPlex Library Preparation Kit from Diagenode.

### ChIP sequencing data analyses

Reads in FASTQ files were first analyzed through a quality control process (fastqc version 0.11.9) to obtain the number of reads. Optical duplicated reads were filtered out using fastx-toolkit (version 0.0.14). Once filtered, FASTQ reads were aligned to the hg38 human genome (index file hg38.4.bt2) using bowtie2 (version 2.4.2) and converted to BAM format using samtools view (version 1.7). Files from different replicates or runs were merged using samtools merge, and duplicates were removed using samtools markdup. The final BAM file was converted to BED using bedtools samtools bamtobedToBed. Peak calling was performed with MACS 3.0 for ChIPs from cell lines or patient-derived xenograft (PDX) tumors (Supplementary Tables S1 and S2).

To assess the overlapping peaks, bed files were uploaded to a Jupyter notebook (Python 3.8.8 and Conda 4.11.0). Datasets were read, processed, and saved using Pandas (version 1.2.4). Afterward, files were uploaded to RStudio (R 4.0.3) to compute the overlapping using BiocManager (version 1.30.17). Functions Granges and make VennDiagram were used to plot the overlapping peak. To perform the filtering of unique peaks, bed files were uploaded to Jupyter Notebook and to a local database with sqlite3 (version 2.6.0). Peaks were annotated using TxDb.Hsapiens-USCD.hg38gh38.knownGene (version 3.2.2).

Prior to mapping, we preprocessed individual FASTQ files from analyses of the PDX tumors with clumpify and fastp clustering reads based on the sequence and removing optical duplicates. For the PDX data filtering out mouse sequences was done as follows: we did map FASTQ reads twice: (i) to human GRCh38.p13hg38 (ii) and mouse GRCm39 genomes using bwa-mem2 aligner. Sorted BAM files were obtained using samtools. Starting with mouse-mapped reads with MAPQ > 0 and with mouse_MAPQ > human_MAPQ, we created text file exclusion lists of read names using a custom Python script employing polars library. We filtered out these mouse reads from the corresponding human BAM files using Picard FilterSamReads. Next step (both for PDX and cell lines); we used samtools to exclude mappings (i) MAPQ < 10 (ii) SUPPLEMENTARY, SECONDARY, and QCFAIL (iii) blacklisted regions (https://www.encodeproject.org/files/ENCFF356LFX/) (iv) non-chromosomal contigs. Prior to merging replicates, we removed the duplicated reads using samtools markdup. Merged sample replicate BAM files underwent another round of duplicate removal using samtools. Narrow peaks were called using MACS3. ChIP-seq QC was done using deepTools. Data processing steps were performed on an SLURM cluster using Nextflow workflow manager.

To calculate genomic distribution of peaks, we used ENSEMBL 109 canonical transcripts, separating it into following sets: (i) protein coding, (ii) lncRNA, and (iii) other (various other RNA types, pseudogenes, etc.). For the first two sets we created putative promoters sets obtaining intervals 1 Kb upstream of the canonical transcription start site using bedtools flank.

ChIP-quantitative polymerase chain reaction (qPCR) data presented in the manuscript were normalized using the percent of input method. To ensure consistency across samples with variable chromatin yields, we first normalized the qPCR values from both ChIP and input samples based on the chromatin concentration (ng/µl) measured after DNA purification. This correction was applied prior to calculating % input and enrichment values and was used consistently across all samples and conditions. This approach helped account for differences in chromatin recovery that could otherwise influence qPCR outcomes. In the figures, we additionally expressed the data as fold enrichment relative to the untreated control condition (e.g. ± heregulin or heregulin plus trastuzumab) to emphasize the biological effects of these treatments on FOXA1 chromatin binding.

### Motif analyses

We used HOMER software for motif discovery to identify the known motif of MCF-7 and BT474 shared FOXA1 chromatin regions (Supplementary Table S3), FOXA1 chromatin regions specific for MCF-7 (Supplementary Table S4), and FOXA1 chromatin regions specific for BT474 (Supplementary Table S5). Enrichment *P*-values reported by HOMER are selected as significant when *P* < 1e^−^100. The motif matrices were retrieved from the JASPAR database [14].

### Transfection and stable cell line generation

Cells were seeded in six-well plate to be 50% confluent upon transfection. Cells were transfected with siRNA targeting FOXA1 (ON-TARGET J-010319-05-0005, Thermo Fisher Scientific), siSIRT6 (Silencer^®^ Select 4392420, Thermo Fisher Scientific), siHDAC1 (Silencer^®^ Select AM51331, Thermo Fisher Scientific), siHDAC2 (Silencer® Select AM167081, Thermo Fisher Scientific), and siControl nontargeting (siNT) (SI03650318 from Promega) using Lipofectamine RNAiMax (Life Technologies) to a final concentration of 55 nM. MCF-7 FOXA1 inducible expression stable cells were reverse transfected with siRNA targeting EP300 (sc-29431, Santa Cruz) and siControl with the same method as siFOXA1 at a final concentration of 10 nM. For the transfection with FOXA1-WT and acetylation mutants, MCF-7 cells were transfected with Lipofectamine 3000 (Invitrogen) following the manufacturer’s protocol, selected with antibiotic, and tested for the expression of FOXA1 by western blot. Expression of FOXA1 was induced with doxycycline (100 mg/ml).

### Immunoprecipitation

Immunoprecipitation was performed with Pierce™ Crosslink IP Kit (26147, Thermo Scientific) by following the manufacturer’s protocol. In brief, 5 μg of HA antibody (St. Cruz; 16B12), FOXA1 (Diagenode; C15410231-100), or IgG (2729, Cell Signaling Technology) was crosslinked to protein A/G beads in each binding column. MCF-7 or MDA-MB-453 cells were lysed with IP Lysis/Wash buffer, and 500 μg of total protein was loaded to each column for immunoprecipitation. Protein lysate and antibody-conjugated beads were incubated together at 4°C overnight followed by three washes with IP Lysis/Wash Buffer. Proteins that were pulled down by corresponding antibodies were eluted with Elution Buffer.

### RNA extraction and qPCR

Total RNA was isolated with TRIzol Reagent (15596018, Invitrogen) following the manufacturer’s protocol. Complementary DNA was synthesized with SuperScript III (18080093, Invitrogen), and real-time PCR was performed with Power SYBR Green PCR Master Mix (4368702, Applied Biosystems). Primers for genes tested and chromatin regions are listed in Supplementary Table S6.

### Western blots

Protein lysate was resolved using precast sodium dodecyl sulfate–polyacrylamide gel electrophoresis (SDS–PAGE) gels and transferred to PVDF membrane. Blots were blocked and incubated overnight at 4°C with primary antibodies. Antibodies used from Santa Cruz Biotechnologies were: ERα (sc-543), p300 (sc-585), ERBB3 (sc-285), HDAC2 (sc-6298), and HA (16B12). Antibodies from Abcam were: FOXA1 (ab55178), ErbB2/Her2 (ab16901), and histone H3 (ab1791). HDAC1 (40967) from Active Motif. Following antibodies from Cell Signaling Technology: RPL13A (2765), β-actin (4970S), phospho-HER2/ErbB2 (Tyr1221/1222) (2243), acetylated-lysine (Ac-K2-100) (9814), phospho-HER3/ErbB3 (Tyr1289; 12D3), and SIRT6 (2590).

### Chromatin fractionation

Cells were grown in the same manner as described in the previous sections. Cells were scraped in cold PBS containing proteinase and phosphatase inhibitors (Thermo Scientific). To isolate the nuclei, the cell pellets are lysed for 10 min in 200 μl of cold buffer A [0.1% Triton X-100 (Sigma–Aldrich), 10 mM Hepes (pH 7.9), 10 mM KCl, 1.5 mM MgCl_2_, 0.34 M sucrose, 10% glycerol, 1 mM DTT, and protease/phosphatase inhibitor cocktail (Thermo Scientific)]. Nuclei are then collected by low-speed centrifugation (4 min, 1300 × *g*, 4°C) and washed once with 200 μl of buffer A (without Triton X-100) followed by another low-speed centrifugation (4 min, 1300 × *g*, 4°C).

Next, nuclei are lysed in 200 μl of buffer B [3 mM ethylenediaminetetraacetic acid, 0.2 mM EGTA, 1 mM DTT, and protease/phosphatase inhibitor cocktail (Thermo Scientific)] and kept on ice for 30 min with occasional vortexing. Afterward, the nuclei are washed three times with buffer B (each with 200 μl of buffer B, separated by centrifugation for 4 min, 1300 × *g*, 4°C). The chromatin pellets are obtained by resuspending isolated nuclei in 200 μl of buffer B and sonicating for 30 s (Bioruptor, Diagenode) to shear the DNA. The chromatin is then pelleted by centrifugation (5 min, 16 000 × *g*, 4°C), and the supernatant is discarded. The pellet is resuspended in 80 μl of buffer B, followed by the addition of 26.6 μl of 4× loading dye (Life Technologies GmbH) and 10.6 μl of 1 M DTT. Typically, 10 μl of protein is loaded per lane on an SDS gel, followed by western blotting to detect the protein of interest.

### PDX models of breast cancer and volume calculation

The serially transplantable luminal-like PDX model MAS98.06 was established by implanting tumor tissue (2–3 mm) in SCID mice as previously described [15]. Mice with tumors of maximally 1 cm^3^ of volume were euthanized and 1–2 mm^3^ pieces of tumor tissues were directly transplanted into the mammary fat pad number 4 on both sides of 4- to 8-week-old female NOD/SCID interleukin-2 receptor gamma chain null (Il2rg^−^/^−^) (NSG) mice. In experimental mice, when the tumor volume was 100–500 mm^3^ the administration of heregulin (5 μg/day), EGF (10 μg/day), or NaCl was initiated by implanting micro-osmotic pumps (Alzet) subcutaneously under general anesthesia. The osmotic pumps were set for drug delivery for up to 28 days. Fulvestrant (Faslodex^®^, 5 mg/mouse), trastuzumab (Herceptin^®^ 0.05 mg/mouse), and/or NaCl were administered by subcutaneous injections twice weekly. Each treatment group included five mice. Animal experiments were performed in multiple batches for logistical reasons; however, care was taken so that mice from different treatment groups were distributed between batches to avoid unwanted batch effects.

Tumor diameters (dmin and dmax) were measured using a caliper and the tumor volume was calculated using the formula (*d_min_^2^*)*(d_max_)*(π/6). All further statistical analyses were conducted with tumor volumes relative to the corresponding tumor volume at time of pump insertion and treatment initiation. As tumor volumes were not measured on the same day after treatment start for all mice, we interpolated the tumor volume at day 20 using the formula from Huang *et al.* [16], based on flanking tumor measurements. Normal distribution of tumor volumes could not be assumed, and we therefore performed nonparametric tests: Kruskal–Wallis test was used to determine whether there was statistical difference between tumor volumes at day 20 between any of the groups. This was followed by post-hoc Dunn’s tests to compare relevant individual treatment groups. The Benjamini–Hochberg procedure was used to correct for multiple testing.

### Proliferation assay

Proliferation assay was carried out by using crystal violet staining of live cells and IncuCyte^®^ Live Cell Imager.

For siRNA-mediated silencing, MCF-7-FOXA1-WT and MCF-7-FOXA1-WD1R cells were reverse transfected with 6 pmols/well of siFOXA1 or siNT (non-targeted) plus 9 μl of Lipofectamine RNAiMAX Transfection Reagent (Invitrogen, 13778150) and 150 μl of Opti-MEM (Gibco, 11058-021). The mixture was allowed to incubate for 20 min at RT and added to each well. Then, 30 000 cells/well were added to each well with 10% FBS DMEM without antibiotics. This mixture was incubated for 16 h at 37°C to allow attachment of cells and reverse transfection. Subsequently, media was replaced with DMEM containing 5% FBS supplemented with doxycycline at 100 ng/μl and/or heregulin beta-1 (SRP3055, Sigma) at 10 nM. Plate was placed on IncuCyte^®^ Live Cell Imager utilizing phase contrast channel with a 10× objective. Sixteen images per well were captured every 24 h for 5 days and cells were retreated every day. Image analysis was conducted using the Incucyte^®^ SX5 2022B software, employing the Al Analysis Software Module (BA-04871). The data were analyzed and presented using GraphPad Prism 8 and Excel.

Proliferation assay to test HDAC2 inhibition was carried out by using crystal violet staining of live cells. MCF-7-FOXA1-WT cells and MCF-7-FOXA1-WD1R cells were seeded in a 24-well plate (1424175, Nunclon™ Delta Surface, Thermo Fisher Scientific) at a density of 40 000 cells per well in triplicate for each condition. As MCF-7 cells expressed a doxycycline-inducible FOXA1 gene, cells were treated for 16 h with doxycycline (D9891, Sigma) at 100 ng/ml to induce expression of FOXA1 wild type (WT) or WD1R FOXA1 mutant deficient in acetylation. Vorinostat (S1047, Selleckchem) (2 µM) and Romidopsin (S3020, Selleckchem) (2 nM) treatments were performed for 24 h. Furthermore, HDAC2 silencing was also carried out by using crystal violet staining of live cells. MCF-7 cells were reverse transfected with 6 pmols/well of siHDAC2 or siNT (non-targeted) plus 9 μl of Lipofectamine RNAiMAX Transfection Reagent (Invitrogen, 13778150) and 150 μl of Opti-MEM (Gibco, 11058-021). This mixture was incubated for 16 h at 37°C to allow attachment of cells and reverse transfection. Subsequently, media was replaced with DMEM containing 5% FBS supplemented with vehicle (as a control treatment) or heregulin beta-1 (SRP3055, Sigma) at 10 nM for 24 h. Afterward, cells were fixed with 2% formaldehyde (252931, PanReac, AppliChem, ITW Reagents) for 20 min and stained with 0.25 mg/ml of crystal violet solution (C6158, Sigma–Aldrich) for 30 min at RT. Cell viability was determined after dissolving the stained cells in 10% acetic acid (1.00063, Sulpeco, Analytical Products) and absorbance was measured at 590 nm using a microplate reader (Infinite 200 PRO, Tecan).

### Statistical analyses

Student’s *t*-test (two-tailed) was applied when comparing two experimental groups with parametric distributions, for data presented in bar or line plots (Figs 2E, 3A–D, and 5A; Supplementary Figs S1E, S3D, S5B, S5D and S5E, S6C, and S7D and S7E). Data are expressed as mean ± standard deviation (SD). Comparisons between matched samples were performed using a paired, two-tailed Student’s *t*-test. Statistical significance was defined as *P* < .05.

**Figure 1.**
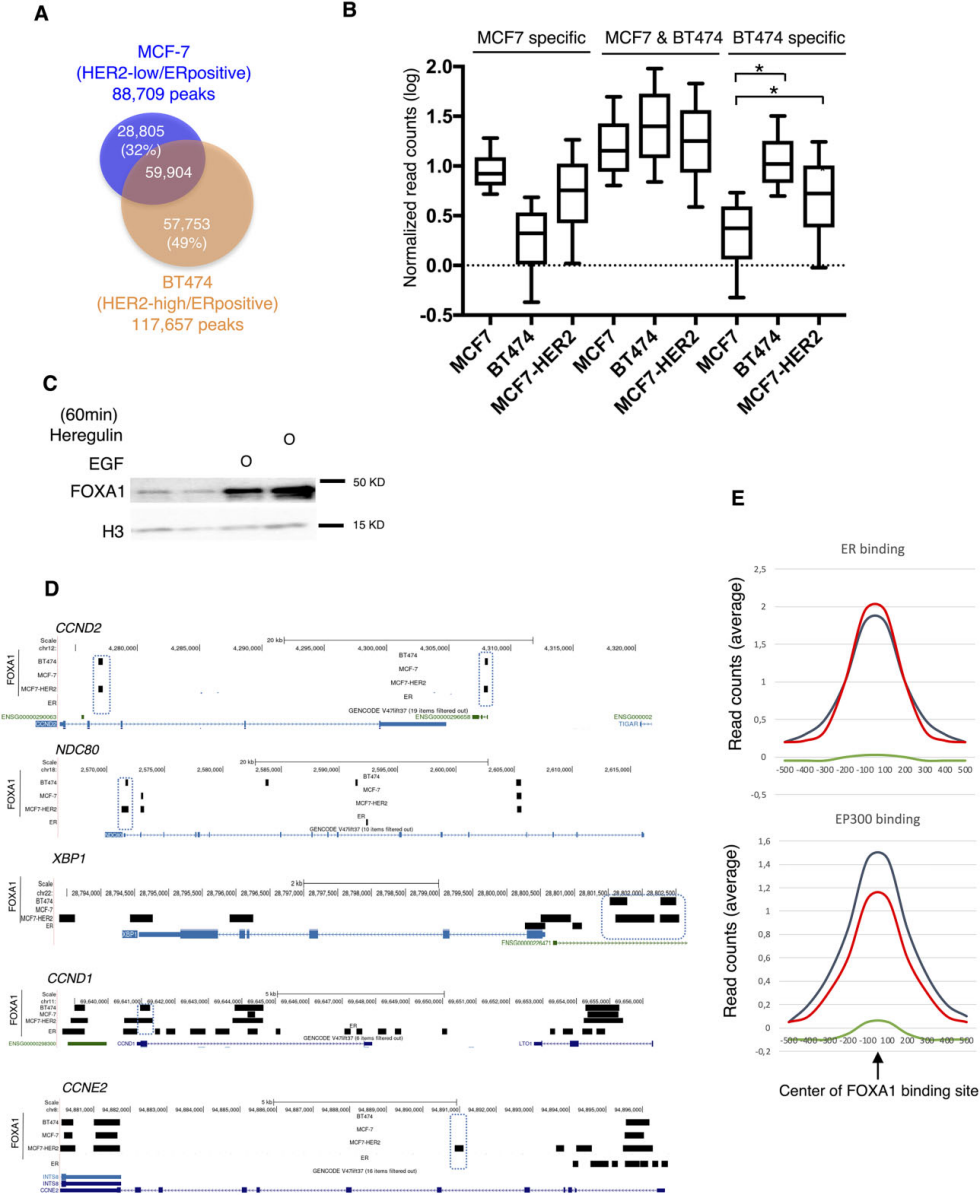
FOXA1 binding to chromatin is increased by HER2. (**A**) Venn Diagram showing the overlap in FOXA1 chromatin interactions (ChIP sequencing) between MCF-7 (*n* = 3) and BT474 (*n* = 3) cells. (**B**) Box plot indicating the average binding intensity of FOXA1 normalized reads (in a logarithmic scale) for peaks from MCF-7 and BT474 cells at chromatin regions identified in MCF-7 and BT474 cells. The FOXA1 binding of MCF-7 cells overexpressing HER2 was also analyzed at the same chromatin regions identified at section A. FOXA1 ChIP reads from 28 411 peaks (MCF-7 unique), 60 298 peaks (MCF-7 and BT474 shared), and 59 915 peaks (BT474 unique) were normalized versus respective inputs. Standard deviation is shown, and Wilcoxon statistical test was performed (5% FDR with two tails). (**C**) FOXA1 western blot from chromatin pellets of BT474 cells treated with growth factors EGF or heregulin for 1 h. H3 was used as a loading control. (**D**) FOXA1 binding sites (in MCF-7, BT474, and MCF-7–HER2 cells) and ER binding sites in (MCF-7 cells) at genes associated with poor prognosis (CCND1, NDC80, XBP1, CCDN1, and CCNE2) in breast cancer patients. (**E**) The relative difference in ER or EP300 signal within the FOXA1 cell type-specific binding sites is shown. The blots show the average binding intensity of ER (upper panel) or EP300 (lower panel) normalized reads (in a logarithmic scale) for peaks from MCF-7 and BT474 cells at chromatin regions identified in MCF-7 and BT474 cells. ER or EP300 reads were subtracted from previous publication [24] using as a reference 28 805 peaks (MCF-7 specific in red), 59 904 peaks (MCF-7 and BT474 shared in dark green), and 57 753 peaks (BT474 specific in green) were normalized versus respective inputs.

To visualize the distribution, central tendency, and variability of the data, box plots were generated for each experimental condition. Box plots display the median (horizontal line) and interquartile range (box). For paired comparisons of matched or repeated measurements, a paired Student’s *t*-test was used when data were parametric (Supplementary Fig. S7B and F). For non-parametric data, the Wilcoxon signed-rank test was employed (Figs 1B and 4B; Supplementary Fig. S6A and B). All statistical tests were two-sided, and a *P*-value of lower than 0.05 was considered statistically significant. Analyses were performed using GraphPad Prism or Microsoft Excel.

Statistical analyses for the *in vivo* data are described in the PDX model section.

## Results

Given the association between HER2 status and the resistance to endocrine treatments, we initially analyzed FOXA1 chromatin interactions in breast cancer cells with varying levels of HER2 (Supplementary Fig. S1A). FOXA1 binding interactions were identified using MACS [17] in HER2-low (MCF-7) and HER2-high (BT474) cell lines. We identified 88 709 FOXA1 binding events in MCF-7 and 117 657 in BT474 cells (Fig. 1A). Comparison of FOXA1 binding between the two cell lines revealed that BT474 cells exhibited the highest number of unique binding sites, accounting for 49% of the total FOXA1 sites identified in BT474. Interestingly, the genomic distribution analysis of FOXA1 unique sites in BT474 revealed an increased enrichment toward promoters (8%) compared to unique sites in MCF-7 (2%) (Supplementary Fig. S1B). To validate the influence of increased HER2 levels on differential FOXA1-binding events observed in BT474 cells, we overexpressed HER2 in MCF-7 cells and performed FOXA1 ChIP-seq. We identified a higher number of FOXA1 binding events in MCF-7–HER2 cells (143 731 peaks) compared to MCF-7 (88 709 peaks) (Supplementary Fig. S1C). Furthermore, the comparison of FOXA1 binding events showed a higher overlap of FOXA1 peaks between BT474 (73%) and MCF-7–HER2 (Supplementary Fig. S1C) compared to the overlap observed between BT474 (51%) and MCF-7 cells (Fig. 1A). The comparative analysis between the unique 59 915 FOXA1 sites of BT474 (upon comparison with MCF-7) and the unique 77 829 FOXA1 sites of MCF-7–HER2 (upon comparison with MCF-7) revealed a high overlap (73% of BT474 FOXA1 sites were shared with MCF-7–HER2 sites) (Supplementary Fig. S1C). Notably, the signal intensity of FOXA1 binding in MCF-7–HER2 cells at chromatin regions specific to BT474 was significantly higher than the signal observed in MCF-7 cells (Fig. 1B). It has been previously reported that heterodimerization of HER2 with EGFR or HER3 is required for HER2 activation. To selectively activate EGFR or HER3, cells were treated with EGF (a specific ligand for EGFR) or heregulin (a specific ligand for HER3), respectively. A global analysis of FOXA1 binding in MCF-7 cells stimulated with these HER2-activating growth factors confirmed that HER2 activation leads to increased FOXA1 binding (Fig. 1C and Supplementary Fig. S1D). Additionally, FOXA1 ChIP and quantitative PCR in MCF-7 cells treated with growth factors demonstrated an increased binding of FOXA1 at chromatin regions uniquely associated with BT474 cells, compared to control-treated cells. This enhanced binding was significantly diminished when MCF-7 cells were co-treated with growth factors and the HER2 inhibitor trastuzumab (Supplementary Fig. S1E).

Next, we aimed to gain insight into the mechanisms underlying specific chromatin binding of FOXA1 in cells with different levels of HER2. Given the differences observed in FOXA1 chromatin binding between HER2-high and HER2-low cell lines, we hypothesized that an additional effect in the former might explain the specific binding of FOXA1 to chromatin. To investigate this, we first identified the DNA motifs associated with shared and cell line–specific FOXA1 chromatin binding sites by comparing MCF-7 and BT474 cells. The comparative analysis revealed that numerous motifs (*n* = 181) were common to both cell lines. In addition, 26 motifs were uniquely enriched at MCF-7–specific FOXA1 binding sites, while 99 motifs were specific to BT474 regions (Supplementary Fig. S2A). Next, we analyzed the presence of recognition motifs for ER (ERE) and FOXA1 (FKH) within the differential FOXA1 binding events detected in HER2-low (MCF-7) and HER2-high (BT474) breast cancer cells. The FKH motifs were highly represented, irrespective of FOXA1-binding regions identified in MCF-7 or BT474 cells. Motif analysis of BT474-specific regions identified chimeric motifs such as FOXA1:AR and NF1:FOXA1, suggesting overlapping binding profiles between FOXA1 and androgen receptor (AR), or between FOXA1 and the transcription factor NF1. In contrast, ERE motifs were underrepresented in FOXA1-specific regions identified in BT474 cells (Supplementary Fig. S2B and Supplementary Tables S3–S5), suggesting that high HER2 signaling influenced FOXA1 to pioneer genomic regions with reduced ER binding and potentially different ER co-regulators. We then determined the binding of ER and the ER co-regulator EP300 to these FOXA1 regions. The results revealed poor enrichment of ER and EP300 in BT474 unique regions (Fig. 1D and E), supporting our hypothesis. We also investigated the frequency of motifs for other pioneer factors previously reported to play a substantial role in breast cancer, such as AP2γ and PBX1 [13]. Significant motif enrichment at all FOXA1 sites in both cell lines was identified for AP2γ but not for PBX1 (Supplementary Fig. S2B). Interestingly, the pioneer function of AP2γ has been reported to be dependent on FOXA1 [18], suggesting that AP2γ might cooperate with FOXA1 in the control of FOXA1 binding to chromatin.

**Figure 2.**
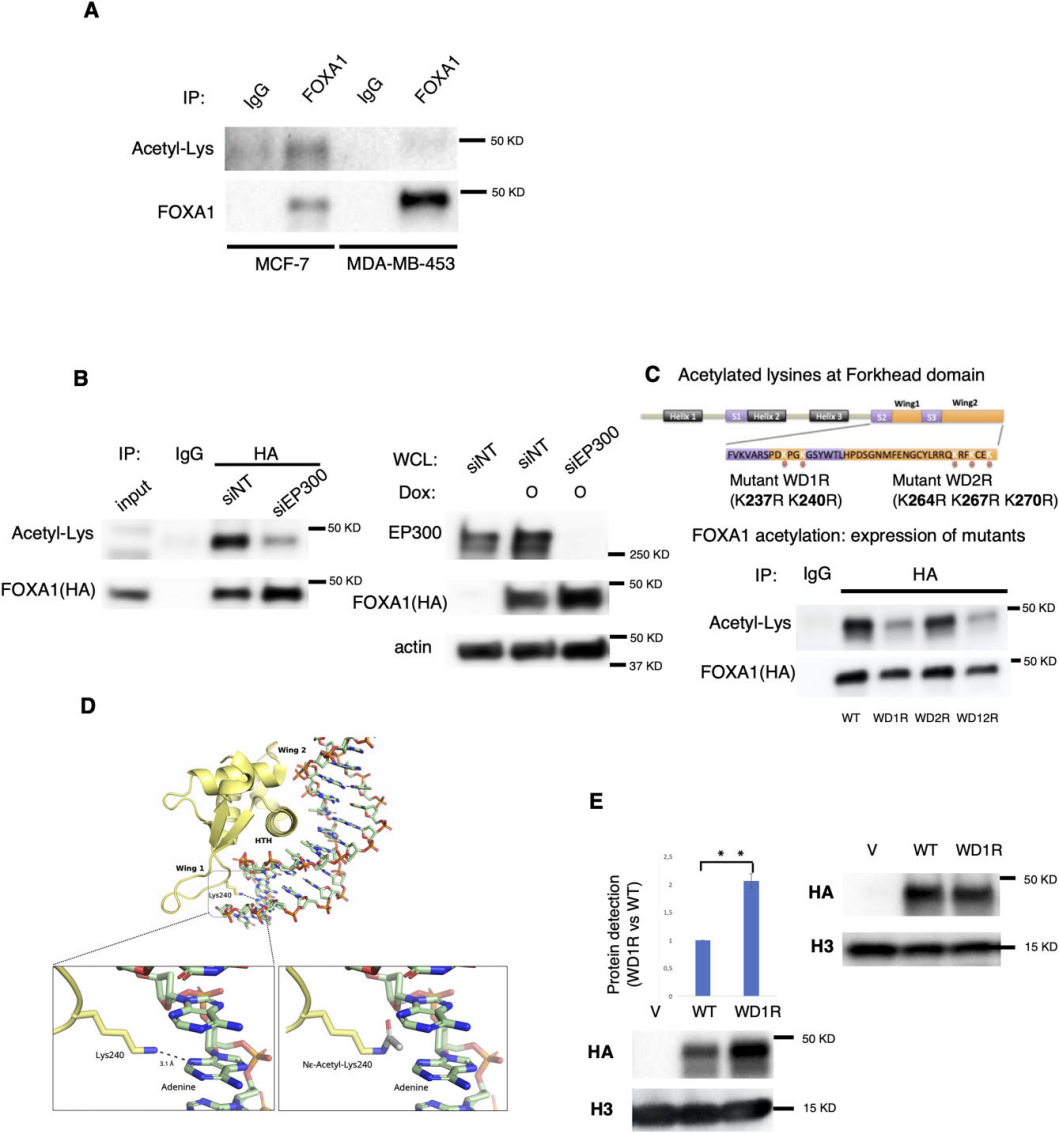
Deacetylation of FOXA1 WDR1 domain induces an increase of FOXA1 binding to chromatin. (**A**) Around 1 mg of protein extracts from MCF-7 (ER positive) and MDA-MB-453 (ER negative) cell lines were isolated to evaluate the acetylation of FOXA1. The protein extract from each cell line was divided in equal parts to mix FOXA1 or IgG (as control antibody) to evaluate FOXA1 acetylation. Immunoprecipitated proteins were blotted against acetyl-lysine or FOXA1 antibodies. (**B**) Stable MCF-7 cells expressing FOXA1-WT (with HA tag) were transfected with siControl RNA or siEP300 RNA. The EP300 protein levels were determined by western blot. Actin was used as a loading control (right panel). FOXA1 acetylation from FOXA1 or IgG IP was determined by western blot (left panel). The total HA-FOXA1 protein levels were also analyzed. (**C**) Potential acetylated lysine amino acids identified at Forkhead domain of FOXA1 (top panel). FOXA1 acetylation from immunoprecipitated HA-FOXA1 protein complex (with HA protein) in MCF-7 cells transfected with WT, lysine mutated into arginine at Wing Domain 1 (WD1R), lysine mutated into arginine at Wing Domain 2 (WD2R), and double mutant (WD12R) (bottom panel). (**D**) Top panel, experimental structure of FOXA1 domains involved in DNA binding (PDB ID 7VOX). The dashed line indicates the interaction between lysine 240 and DNA. Bottom-left square, amplified detail of the experimental structure showing the interaction between lysine 240 and the adenine (dashed line). Bottom-right square, computational model showing that the acetylation of lysine 240 would neutralize the side chain charge, preventing the interaction between lysine 240 and the adenine. These figures have been produced in PyMol (The PyMol Molecular Graphics System, Version 2.0 Schrödinger, LLC.). (**E**) The global binding of FOXA1 to the chromatin fraction protein pellet in MCF-7 cells transfected with WT and WD1R FOXA1 mutant was tested. Left panel, is shown the global FOXA1 binding at chromatin fraction (by western blot with HA antibody). Right panel is shown the FOXA1 expression in all the conditions (by using HA antibody). T-test: WDR1 versus control: **(*P *< .001).

Altogether, our findings suggest that ER with EP300 are less likely to bind to HER2-induced regions, proposing that EP300-mediated acetylation of FOXA1 may regulate FOXA1 binding at these chromatin regions. To test our hypothesis, we initially analyzed the acetylation of FOXA1 in ER+ (MCF-7) and ER-negative (MDA-MB-453) breast cancer cell lines. We conducted immunoprecipitation of FOXA1 followed by western blot analysis of FOXA1 and acetyl-lysine modification. FOXA1 was only acetylated in the ER+ cell line (Fig. 2A), and this acetylation was dependent on EP300 (Fig. 2B). A previous study [19] has reported that FOXA1 contains five putative acetylation sites identified at the wings of the fork-head domain: two at wing 1 (WD1) and three at wing 2 (WD2). To determine which FOXA1 domains were acetylated in ER+ cells, we created FOXA1 mutants defective for acetylation at WD1 (WD1R) or WD2 (WD2R) and compared their acetylation to wild-type FOXA1 (WT) transfected cells. We also created a mutant defective for both domains (WDR12) (Fig. 2C, top panel). Both FOXA1 WT and WD2R mutant were acetylated, but the acetylation of the WD1R mutant was reduced (Fig. 2C, bottom panel), indicating that lysines at the WD1 domain are acetylated in ER+ cells.

Next, we aimed to determine the physiological relevance of FOXA1 acetylation in ER+ cells. It is well established that lysine acetylation neutralizes the positive charge of the lysine side chain and increases its steric bulk. Consequently, not only the protein charge affected, but also the protein–DNA binding affinity might also be attenuated [19]. Importantly, FOXA1 interacts with DNA through a helix-turn-helix (HTH) structure and the wing domains WD1 and WD2 [20]. A previously published structure of FOXA1 DBD in complex with p53 DNA [21] showed that lysine 240 contributes to FOXA1 DNA binding through the formation of hydrogen bonds between the primary amine group of the lysine side chain, an adenine base, a ring oxygen in the DNA backbone, and a thymine on the complementary strand (PDB ID: 7VOX). In order to gain insight into the effect of acetylation of lysine 240, we carried out structural modeling of the modified residue. This analysis indicated that, in the same configuration of the lysine side chain, acetylation of lysine 240 would result in steric clash between the acetyl group and the adenine. In addition, the close proximity of the carbonyl group and the ring oxygen (1.4 Å) would also lead to steric strain due to repulsion between the lone electron pairs (Fig. 2D). This, together with the fact that expression of FOXA1 deficient in the acetylation of the WD1 domain (WD1R) increased the global binding to chromatin compared to FOXA1 WT (Fig. 2E), suggests that acetylation of lysine 240 by EP300 destabilizes the binding of FOXA1 to DNA. Altogether, the results of this section suggest that EP300 mediates the acetylation of FOXA1 in the WD1 domain of ER+ cells to restrict FOXA1 genomic distribution and facilitate ER function.

**Figure 3.**
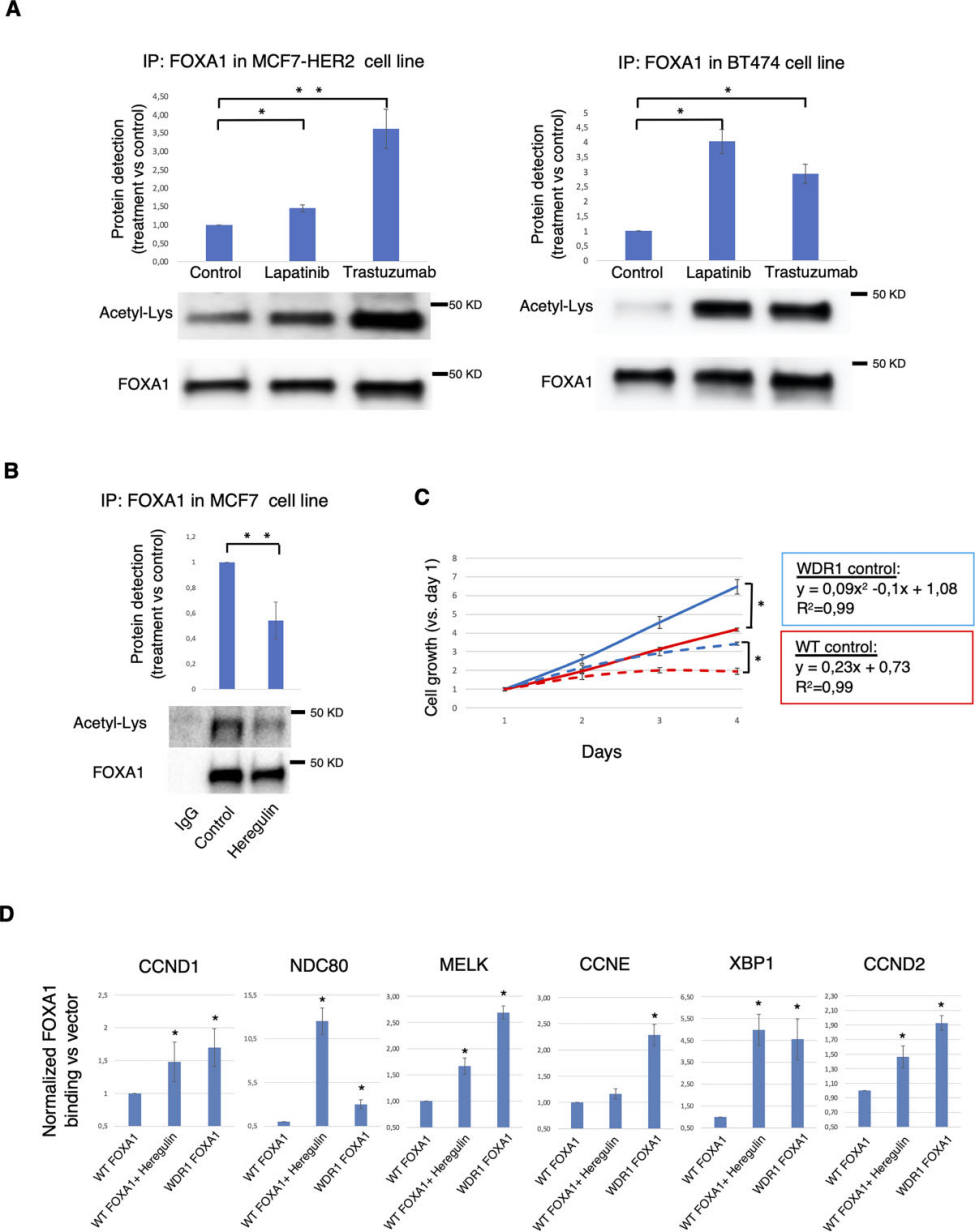
FOXA1 deacetylation is regulated by HER2/3 signaling and confers insensitivity to anti-ER therapy. (**A**) FOXA1 acetylation from immunoprecipitated FOXA1 protein was determined by western blot in MCF-7–HER2 and BT474 cell lines and compared to nontreated cells (Veh) or cells treated with anti-HER2 drugs Lapatinib (Lap) or Trastuzumab/Herceptin (Her). Protein quantification was determined for each experimental condition (from at least two replicates) and *t*-test (two tails) was applied to determine statistical differences. T-test: Lapatinib or Trastuzumab versus Control: *(*P *< .01), **(*P *< .001). (**B**) FOXA1 acetylation from immunoprecipitated FOXA1 protein complex in MCF-7 cells treated with heregulin. Protein quantification was determined for each experimental condition (from at least two replicates) and *t*-test (two tails) was applied to determine statistical differences. T-test: Heregulin versus control: **(*P *< .001) (**C**) MCF-7 cells were stably transfected with WT FOXA1 (blue line) and WD1R mutant (red line) upon a doxycycline-inducible vector. Cells were treated with fulvestrant (1 μM; dashed blue lane for WT and dashed red lane for WD1) and cell growth was monitored by Incucyte *in vivo* system. Left panel shows equations measuring growth trend of FOXA1 WT cells (linear) or FOXA1 WD1R cells (logarithmic) and their respective *R*^2^. T-test: FOXA1 WT versus FOXA1 WDR1: *(*P *< .01). Fulvestrant: FOXA1 WT versus FOXA1 WDR1: *(*P *< .01). (**D**) FOXA1 binding to chromatin (by using HA-antibody) at HER2-enriched sites in MCF-7 cells transfected with FOXA1-WT and treated with heregulin (1 h) or in MCF-7 cells transfected with FOXA1 WD1R mutant. As control (vehicle) FOXA1 WT cells were used. Data represent fold change of FOXA1 binding (WD1R or WT with heregulin) versus control. The data are the mean of 3 independent replicates ± SD. T-test (two tails) was applied to determine statistical differences. T-Test: *: FOXA1 WT + Heregulin or FOXA1 WD1R versus vector (*P *< .05).

Next, we investigated how FOXA1 acetylation is influenced by HER2. First, we examined the impact of HER2 inhibition on the acetylation of FOXA1 in ER+ breast cancer cell lines with high HER2 levels (BT474 and MCF-7–HER2). The inhibition of HER2 increased the acetylation of FOXA1 in these cell lines (Fig. 3A and Supplementary Fig. S3A and B). Subsequently, we treated MCF-7 cells with heregulin and performed FOXA1 immunoprecipitation. The results revealed that HER2/3 activation prevented FOXA1 acetylation at WD1 in MCF-7 cells (Fig. 3B). Overall, these results support the notion that HER2/3 and ER play opposing roles in the regulation of FOXA1 acetylation.

FOXA1 mutations in the wing domains have been previously identified and shown to produce distinct chromatin profiles that influence therapeutic response in breast cancer [22]. Specifically, lysine residues are rarely mutated according to available public databases (e.g. COSMIC). However, the following mutations have been identified in patients: K237E, K237R, K237N, K240T, and K267E. All these mutations are missense changes and were identified in individual patients. While substitution of lysine 240 for a threonine would not result in steric clash, the short size of the side chain of threonine would hinder the formation of hydrogen bonds with the DNA bases, as suggested by structural modeling (Supplementary Fig. S3C). Therefore, the presence of this mutation in patients may affect the ability of FOXA1 to interact with chromatin at its potential DNA-binding sites.

To determine whether deacetylated FOXA1 might affect response to endocrine therapy, we investigated the growth ability of ER+/HER2-low cells when they ectopically expressed a FOXA1 mutant defective in acetylation at WD1 domain (WD1R). Furthermore, the experiment was analyzed by comparing the following conditions: control treated cells or treated with ER degrader (fulvestrant). As the figure shows, the growth is affected in cells expressing the WD1R mutant when compared to the WT (Fig. 3C). Our findings indicate that cells expressing the FOXA1 mutant deficient for acetylation at WD1 domain, show a different pattern of cell growth. Whereas the WT cells growth follows a lineal trend (*R*^2 ^= 0.99), the WD1R growth follows an exponential trend (*R*^2 ^= 0.99), indicating that WD1R cells grow faster than WT cells. By contrast, by adding the ER degrader fulvestrant (at day 1), the WT cells stop growing but cell growth is still maintained in cells (although slowed) expressing the WDR1 mutant, reaching a significant difference in cell growth confluence at day 4 (Fig. 3C). Altogether, these findings support the notion that deacetylation of the FOXA1 WDR1 domain enables FOXA1 binding to chromatin regions induced by HER2/3, providing a growth advantage. In fact, FOXA1 binding in MCF-7 cells is induced at HER2-regulated chromatin regions of genes associated with poor prognosis in breast cancer, either when the WDR1 domain is mutated or when HER2/3 signaling is triggered (Fig. 3D). To validate that the effect of FOXA1 deacetylation upon HER2/HER3 activation is less likely dependent on ER, we evaluated the expression of genes associated with worse prognosis in breast cancer upon heregulin and fulvestrant treatment for 3 h. The expression of all the genes tested indicated a significant increase of expression upon heregulin stimulation (Supplementary Fig. S3D).

**Figure 4.**
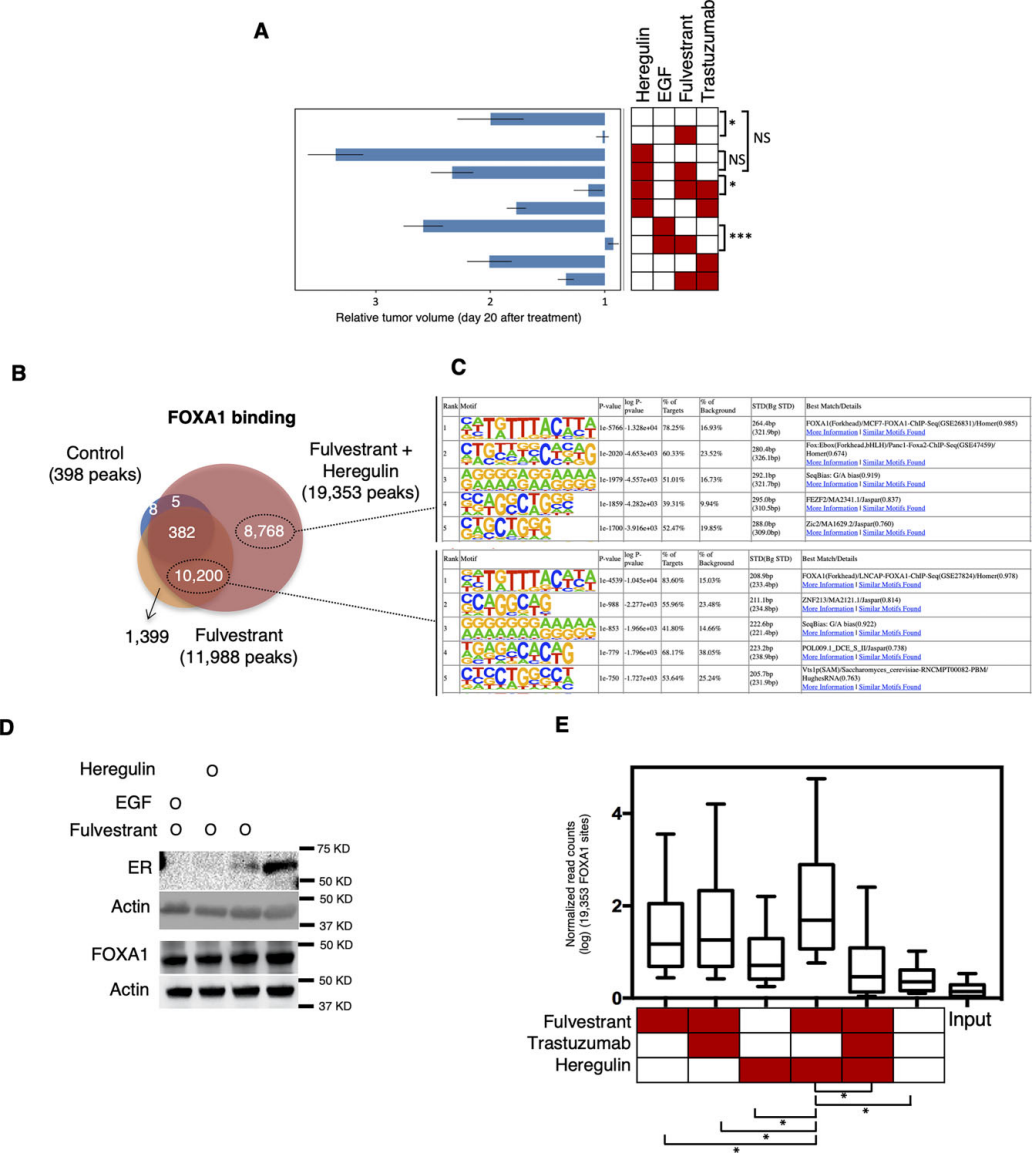
HER2/3 induces tumor growth *in vivo* and gain of FOXA1 binding to chromatin independently of ER. (**A**) Change in PDX tumor volume across the first 20 days after pump insertion and initiation of treatment (five mice per condition). The bars indicate tumor volume at day 20 relative to treatment start, and the heatmap illustrates the corresponding treatment combinations. * (*P* < .05), *** (*P* < .001), NS (not significant). (**B**) Venn diagram showing the overlap in FOXA1 chromatin interactions (ChIP sequencing) in PDX tumors from two replicates of control animals (398 peaks) or treated with fulvestrant (11 988 peaks) or treated with fulvestrant and heregulin (19 353 peaks). (**C**) Top 5 motifs identified by Homer motif analysis within FOXA1 unique chromatin regions identified with fulvestrant (10 200 peaks) or heregulin + fulvestrant (8768 peaks). (**D**) Western blot of ER, FOXA1, and β-actin (loading control) from protein extract of PDX tumors from animals control-treated or animals treated with fulvestrant or treated with heregulin and fulvestrant or treated with EGF and fulvestrant. (**E**) Box plot indicating the average binding intensity of FOXA1 normalized reads (in a logarithmic scale) from 19 353 FOXA1 peaks identified in fulvestrant- and heregulin-treated animals. The FOXA1 binding intensity from ChIP-seq PDX tumors of animals with different treatments. Wilcoxon rank-sum test was used to test any statistical difference between samples (5% FDR with two tails; *[*P *< .01)].

To further confirm our findings, we used an *in vivo* luminal-like breast cancer PDX mouse model [15]. We examined the binding of FOXA1 and the response to anti-ER therapy in tumors with increased HER2 signaling. To selectively activate EGFR or HER3, mice were treated with EGF (a specific ligand of EGFR) or heregulin (a specific ligand of HER3). Exposure of the animals to the growth factors EGF or heregulin resulted in HER2 activation through phosphorylation (Supplementary Fig. S4A). Mice were treated with vehicle, fulvestrant, or combinations of fulvestrant with growth factors. Tumor size was measured regularly, and relative tumor volume at 20 days post-treatment was compared across groups. A Kruskal–Wallis test indicated a significant difference in tumor volume across treatment groups (*P *< .001). We then conducted post-hoc pairwise comparisons between relevant treatment groups using Dunn’s tests. As expected, fulvestrant significantly inhibited tumor growth compared with vehicle controls (*P* = .024), whereas tumors in control mice approximately doubled in volume within 20 days (Fig. 4A). Heregulin treatment promoted tumor growth, while EGF induced a modest increase relative to controls. When combined with fulvestrant, EGF did not interfere with growth suppression, resulting in volumes comparable to fulvestrant alone (*P* < .001). In contrast, heregulin markedly reverted the efficacy of fulvestrant, producing tumor growth rates close to controls. However, tumor volume in the fulvestrant + heregulin group was not significantly different from heregulin alone (*P* = .13). Addition of trastuzumab to fulvestrant + heregulin restored growth inhibition (*P* = .02), bringing tumor volumes close to those seen with fulvestrant monotherapy. We corroborated these findings in breast cancer cell lines (Supplementary Fig. S5A–C). HER2/HER3 activation in these cells rescued the growth arrest induced by the ER inhibitor fulvestrant, an effect that was prevented by genomic depletion of FOXA1 (Supplementary Fig. S5D and E). These results indicate that HER2/HER3 activation led to insensitivity to hormone, as observed with FOXA1 deacetylation at the WDR1 domain.

To investigate whether *in vivo* HER2/HER3 signaling influences FOXA1 chromatin binding, we performed FOXA1 ChIP-seq in PDX tumors and compared binding profiles across different treatment groups. FOXA1 gained chromatin-binding sites in tumors treated with fulvestrant (Fig. 4B), with these regions enriched for forkhead motifs (Fig. 4C). Notably, co-treatment with fulvestrant and heregulin resulted in an even greater gain of FOXA1 binding (Fig. 4B), with regions enriched for forkhead motifs (Fig. 4C). These treatments did not alter FOXA1 protein levels. On the contrary, fulvestrant reduced ER protein levels (Fig. 4D). This HER2/HER3-induced binding was significantly reduced by HER2 inhibition (Fig. 4E). To further assess the HER2/HER3-mediated modulation of FOXA1 binding, we compared FOXA1 ChIP-seq signals in MCF-7 and MCF-7–HER2 cells at regions identified in PDX tumors treated with heregulin and fulvestrant. Based on prior observations of reduced ER binding under these conditions (Fig. 1), we hypothesized that HER2/HER3 activation facilitates FOXA1 recruitment to additional chromatin regions. For that, we analyzed the FOXA1 binding intensity of MCF-7 or MCF-7–HER2 at heregulin-induced regions observed in PDX tumors. Supporting this, FOXA1 binding was significantly elevated in MCF-7–HER2 cells relative to parental MCF-7 cells at these PDX-derived sites (Supplementary Fig. S6A). To validate the ER-independent role of FOXA1 triggered by heregulin, we examined the expression of genes associated with poor breast cancer prognosis. Importantly, they localized near HER2/HER3-driven FOXA1 binding sites (Supplementary Fig. S6B). Upon treatment with fulvestrant alone or in combination with heregulin, we observed upregulation of all tested ER-regulated genes, with a further significant increase following heregulin stimulation (Supplementary Fig. S6C). Collectively, our *in vivo* and *in vitro* findings demonstrate that FOXA1 chromatin binding is regulated by HER2/HER3 signaling via acetylation-dependent modulation of the WD1 domain. This signaling axis promotes FOXA1 binding at ER-independent sites and contributes to resistance to endocrine therapy.

**Figure 5.**
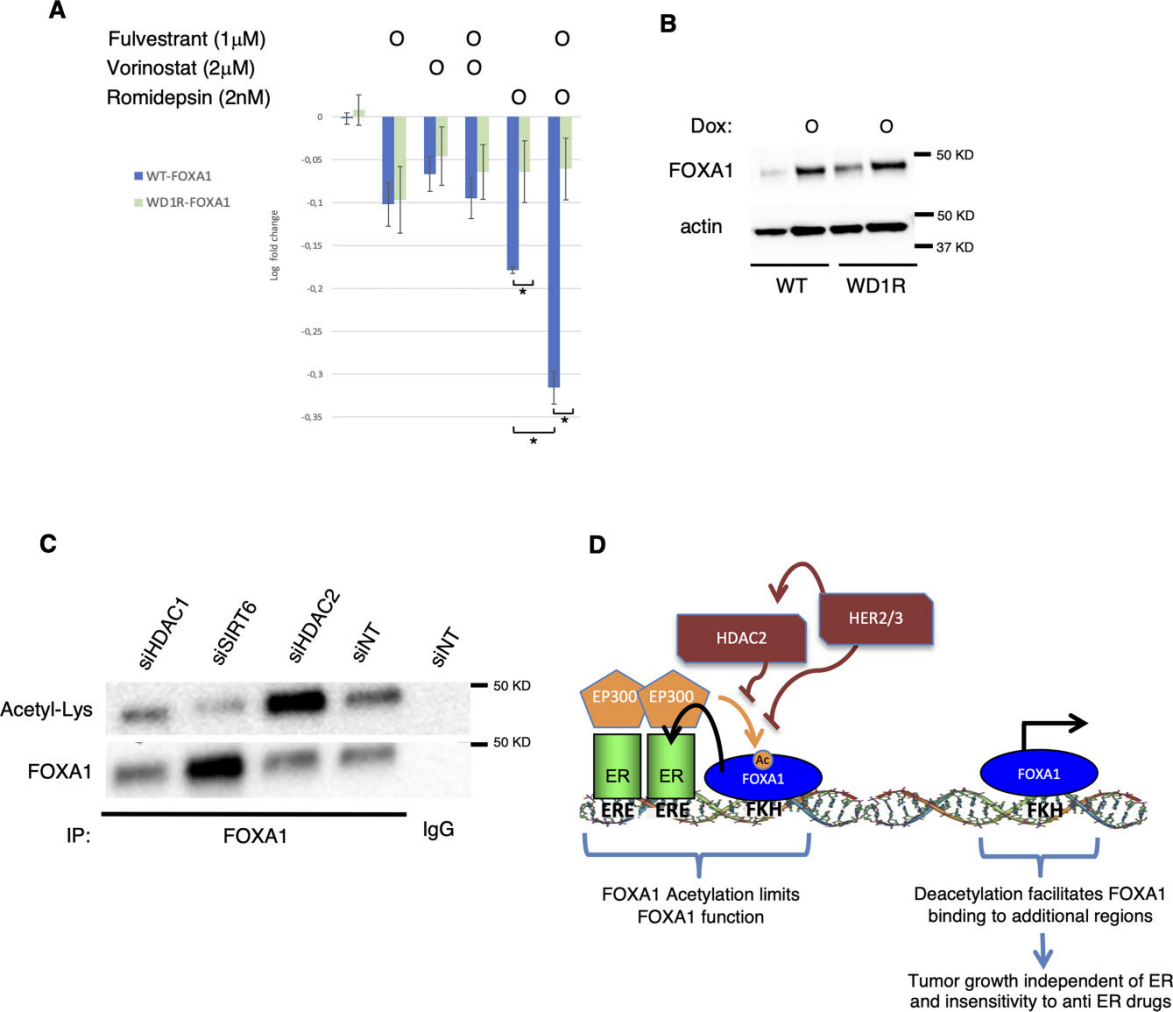
Inhibition of HDAC2 restores FOXA1 acetylation and inhibits cell growth. (**A**) Cell growth of MCF-7 cells stably transfected with FOXA1-WT (blue bar) or FOXA1 WD1R (green bar) mutant. Cells were control-treated (vehicle), treated with a specific HDAC1/2 inhibitor (romidopsin), or treated with a general HDAC inhibitor (vorinostat). Cells were also treated with fulvestrant alone or in combination with HDAC inhibitors for 24 h. Data are represented as logarithmic fold change of cell growth versus control treatment. The data are the mean of at least four independent replicates ± SD. T-test (two tails) was applied to determine statistical differences. T-test: Romidepsin WT versus WD1R: * (*P *<  003); WT Romidepsin versus fulvestrant + WT Romidepsin: * (*P *< .002); Romidespsin + fulvestrant WT versus WD1R: * (*P *< .002). (**B**) Western blot of FOXA1 and β-actin of FOXA1 WT and WDR1 MCF-7 expressing cells upon doxycycline treatment. (**C**) FOXA1 acetylation from immunoprecipitated FOXA1 protein complex in MCF-7 cells transfected with siRNA of HDAC2 or siHDAC1. As a control, siRNA of nontargeting control gene (siNT) and targeting acetylase SIRT6 (siSIRT6) was used. (**D**) Model of FOXA1 binding to chromatin regulated by acetylation and HER2 signaling.

Next, we explored whether inhibition of histone deacetylation affects the function of FOXA1. We start testing the impact of deacetylase inhibitors on the growth of HER2-low MCF-7 cells expressing either WT FOXA1 or a mutant FOXA1 protein that is deficient in acetylation at the WD1 domain (WD1R). We used a broad-spectrum HDAC inhibitor targeting classes I, II, and IV (vorinostat) and a selective inhibitor of HDAC1/2 (romidepsin), which belong to class I HDACs. MCF-7 cells expressing FOXA1 WT or the WD1-domain mutant were treated with these inhibitors alone or in combination with the ER degrader fulvestrant. The results showed that both romidepsin and fulvestrant inhibited cell growth in cells expressing FOXA1 WT. In contrast, the growth-inhibitory effect of romidepsin was significantly attenuated in cells expressing the FOXA1 WD1R mutant (Fig. 5A and B). Interestingly, the combination of fulvestrant with the selective HDAC1/2 inhibitor romidepsin had a stronger effect inhibiting cell growth than the single treatment with romidepsin (Fig. 5A). To assess which deacetylases regulate FOXA1 acetylation, we performed immunoprecipitation of FOXA1 in MCF-7 cells transfected with siRNA targeting HDAC1 or HDAC2. As controls, we included cells transfected with nontargeting siRNA and cells depleted of Sirtuin 6 (SIRT6), a non-class I HDAC (Fig. 5C). We observed increased FOXA1 acetylation upon HDAC2 knockdown, compared to both control cells and cells with knockdown of the other deacetylases tested (Fig. 5C and Supplementary Fig. S7A). We confirmed the cooperative role of ER and HDAC2 inhibition in limiting cell growth when combining genomic depletion of HDAC2 in combination with fulvestrant treatment (Supplementary Fig. S7B and C).

Finally, to determine whether HDAC2 mediates the effects of HER2/3 signaling on FOXA1 function, we examined FOXA1 protein levels, acetylation, and chromatin binding in MCF-7 cells transfected with either control or HDAC2-targeting siRNA. Western blot analysis confirmed efficient genomic depletion of HDAC2 without altering substantially the total FOXA1 protein levels (Supplementary Fig. S7C). Immunoprecipitation experiments showed increased FOXA1 acetylation in HDAC2-depleted cells compared to control transfected cells (Supplementary Fig. S7D), consistent with the role of HDAC2 in deacetylating FOXA1. Treatment with heregulin for 1 h reduced FOXA1 acetylation in both control and HDAC2-depleted cells, although acetylation levels remained elevated in the HDAC2-depleted cells (Supplementary Fig. S7D). ChIP assays revealed that heregulin treatment enhanced FOXA1 chromatin binding in control cells. However, this effect was abolished in HDAC2 knockdown transfected cells and treated by heregulin. This effect might be explained by the elevated levels of FOXA1 acetylation. These findings indicate that while HDAC2 deacetylates the WD1 domain of FOXA1, its depletion prevents FOXA1 recruitment to chromatin in response to heregulin (Supplementary Fig. S7E), impairs the cell growth response triggered by heregulin (Supplementary Fig. S7F).

## Discussion

In this study, we provide evidence that FOXA1 chromatin binding is dynamically regulated by its acetylation state. In HER2-low cells, FOXA1 is acetylated by EP300 and co-occupies ER-enriched regions, supporting ER signaling. However, activation of the HER2/HER3 pathway or the expression of HDAC2 inhibits FOXA1 acetylation, leading to its binding to additional chromatin regions with reduced ER occupancy and conferring insensitivity to anti-ER drugs (Fig. 5D). Previous studies have indicated that ER can influence FOXA1 chromatin binding [23]. In our work, we demonstrate that ER/EP300 restricts FOXA1 binding and offers a potential mechanistic explanation for how ER controls FOXA1 binding. While the ER/EP300 complex appears to restrict the binding landscape of FOXA1 to chromatin, our motif analysis of HER2-high BT474-specific FOXA1 binding regions suggests that transcription factors such as AR and NF1 may help recruit deacetylated FOXA1 to these novel sites. The enrichment of FOXA1:AR and NF1:FOXA1 chimeric motifs, combined with the paucity of EREs, supports the hypothesis in which AR and NF1 functionally cooperate with FOXA1 to establish an ER-independent regulatory program. Future experiments might address this question.

Furthermore, our findings suggest that alterations in the function of EP300 and HDAC2 may influence FOXA1. The role of EP300 in mediating ER transcription is well established. As a component of the ER co-activator complex [24], the histone acetyltransferase activity of EP300 influences ER gene expression [25]. Additionally, the interaction between ER and chromatin is mediated by FOXA1 [1]. Thus, by mediating the acetylation of FOXA1, EP300 would facilitate the transcription of ER-regulated genes. This effect would be the consequence of limiting acetylated FOXA1 to ERE-containing regions. Therefore, by acetylating FOXA1, ER/EP300 would enhance the expression of ER target genes. In contrast, when FOXA1 is deacetylated by HDAC2, it may bind to chromatin regions that are less likely to be enriched with ER binding, driving transcription independently of ER in breast cancer. In agreement with our data, clinical reports have shown that concomitant treatment with HDAC inhibitors and aromatase inhibitors (AIs) significantly improves progression-free survival in hormone receptor–positive advanced breast cancer patients who have progressed on previous endocrine therapies [26].

Moreover, our study addressed whether HDAC2 and HER2/3 regulate FOXA1 through independent pathways or as part of a convergent mechanism. We found that FOXA1 chromatin binding in response to heregulin requires functional HDAC2. Recent research has explored the connection between HER2 activation and HDAC2 activity, especially in breast cancer therapies. One key finding is that HER2 activation influences HDAC2 activity, contributing to therapeutic resistance [27]. HDACs, including HDAC2, are involved in epigenetic regulation and have been implicated in cancer cell survival and proliferation by modifying chromatin structure. Studies suggest that HDAC inhibition can reverse resistance to HER2-targeted therapies [28]. For instance, when HER2 signaling is activated, HDAC2 becomes phosphorylated and inhibited through downstream pathways involving MAPK and PKC, which are crucial for cell survival and growth. In resistant cancer cells. However, HDAC2 can be reactivated, repressing tumor-suppressor gene expression, such as PHLDA1. The combination of HDAC inhibitors with HER2-targeted therapies has been shown to restore sensitivity to treatments like lapatinib by reactivating PHLDA1 expression [28]. Our findings also indicate that HER2/3 signaling partially reduces FOXA1 acetylation in HDAC2-reduced cells, supporting a model in which HER2/3 orchestrates FOXA1 activity both by modulating its acetylation state through HDAC2 activation and via an HDAC2-independent mechanism (Fig. 5D). Previous studies have reported that ER is negatively regulated by HER3-mediated signaling [29]. Considering that EP300 is recruited to chromatin by ER, it is plausible that HER2/3 activation may also suppress EP300 activity. Altogether, our findings highlight the interplay between HER2 activation, HDAC2, and EP300 as a potential mechanism of FOXA1 regulation. Therefore, HER2/3 signaling appears to converge on both EP300 and HDAC2 to coordinate a deacetylated, chromatin-competent FOXA1 state, enabling transcriptional responses to mitogenic cues. This dual-layered regulation may provide a robust mechanism to fine-tune FOXA1 activity in hormone-independent breast cancer contexts.

Recent studies have highlighted the complex role of FOXA1 mutations in the response to endocrine treatments in breast cancer [22]. FOXA1, a pioneer transcription factor that influences ER activity, plays a crucial role in mediating the effects of endocrine therapy [3]. Mutations in FOXA1 have been linked to both increased and decreased sensitivity to these treatments, depending on the mutation site. For example, the study by Arruabarrena-Aristorena *et al.* [22] identified several hotspot mutations in FOXA1, particularly in the Wing2 region and the SY242CS mutation in the third β strand. These mutations have been associated with altered chromatin profiles and varying responses to AIs. The SY242CS mutation, in particular, exhibits neomorphic properties, altering chromatin binding in a way that opens new chromatin regions. This mutation leads to the activation of an alternative cistrome and transcriptome. Structural modeling suggests that the SY242CS mutation changes FOXA1 protein conformation, allowing it to bind to a non-canonical DNA motif, thereby contributing to therapeutic resistance. Interestingly, analysis of FOXA1 mutations in patients has not revealed frequent mutations at lysines located within any Wing domain. K240T in the WD1 domain, where lysine is replaced by threonine, is the most significant lysine mutation identified in tumors. In this work, we examined the interaction between lysine 240 and adenosine, a nucleotide that can potentially interact with lysine, threonine, asparagine, and glutamine residues according to computational analysis. Notably, threonine is the only amino acid that establishes direct recognition with adenosine in the same location as lysine [30]. The K240T mutation in FOXA1 would result in a lack of interaction between this residue and the DNA, as shown in our structural modeling, if the conformation of the WD1 is preserved. This is mainly due to the smaller size of the threonine side chain. However, due to the flexibility of the WD1 domain and the affinity of threonine for adenine, the K240T mutation could preserve the interaction and putative DNA binding *in vivo*. Altogether, our findings, along with the mutational analysis of FOXA1, reveal that understanding the alterations of FOXA1 that affect its chromatin interactions is crucial. These alterations can ultimately impact endocrine therapy resistance, which is vital for developing more personalized treatment strategies for patients with ER+ breast cancer.

Finally, the results of this study expand the role of HER2 beyond its previously reported direct interaction with ER [31]. We demonstrate that luminal-like breast tumors can acquire the ability to grow in an ER-independent manner when the HER2/HER3 signaling pathway is enhanced. Additionally, our findings establish that FOXA1 mediates HER2/HER3 signaling in a hormone-resistant context. We provide *in vivo* evidence that increased HER3 activity overcomes the inhibitory effects of ER degrader fulvestrant, enabling ER+ tumors to grow independently of ER. Consistent with preclinical [32] and clinical [33] studies showing that combining anti-ER therapies with novel anti-HER2 treatments delays resistance development, our *in vivo* experiments reveal that the HER2 inhibitor trastuzumab does not enhance the repressive effect of fulvestrant monotherapy on tumor growth. However, a benefit from dual ER and HER2 inhibition, with trastuzumab and fulvestrant, is observed when animals are treated with heregulin, suggesting that HER2 together with ER inhibition is more effective when the HER2/HER3 pathway is activated. This aligns with the idea that the HER3/HER2/FOXA1 axis can circumvent the inhibition of the EGFR/ER pathway [34]. In this context, clinical trials targeting HER2-low tumors (expressing low levels of HER2 and HER3) show improved responses in patients treated with anti-HER3 conjugated antibodies, such as patritumab deruxtecan [35].

Altogether, our study suggests that ER inhibition prevents tumor growth driven by EGFR/HER2 signaling, supporting the potential of combination therapies targeting both ER and HER2. However, HER3-mediated signaling undermines this effect, representing a key mechanism of resistance to anti-HER2 therapies [36]. Our findings reinforce the importance of targeting the HER3/HER2/FOXA1 axis to overcome resistance associated with the EGFR/ER pathway.

## Supplementary Material

gkaf1086_Supplemental_File

## Data Availability

Sequencing data are deposited to ENA with project identifier PRJEB79050 (ERP163266 FOXA1 ChIP-seq in PDX, MCF-7, and BT474 cell lines).
