# Supplementary material for: The harmonized activities of HER2–HER3 heterodimer and deacetylated FOXA1 evade hormone response by regulating FOXA1 chromatin binding
Source: Nucleic Acids Res. 2025 Nov 13;53(20):gkaf1086. doi: 10.1093/nar/gkaf1086 (PMC12611346; doi:10.1093/nar/gkaf1086)
Supplement: gkaf1086_Supplemental_File [file gkaf1086_supplemental_file.pdf]

Supplementary Table 1

List of sequencing files

ChIP experiment	Figures	ChIP sequencing file name	input sequencing file name
BT474 FOXA1 ChIP-seq	1A and Suppl 1B	Wang_BT474_chip__FOXA1_CTRL.50bp.rep1.r1	Wang_BT474_chip__INPUT.50bp.rep1.r1
	1A and Suppl 1B	Wang_BT474_chip__FOXA1_CTRL.76bp.rep1.r1	Wang_BT474_chip__INPUT.76bp.rep1.r1
	1A and Suppl 1B	Wang_BT474_chip__FOXA1_CTRL.76bp.rep2.r1	Wang_BT474_chip__INPUT.76bp.rep2.r1
	1A and Suppl 1B	Wang_BT474_chip__FOXA1_CTRL.76bp.rep3.r1	
MCF-7 FOXA1 ChIP-seq	1A and Suppl 1B	Wang_MCF7_chip__FOXA1_CTRL.50bp.rep1.r1	Wang_MCF7_chip__INPUT_CTRL.50bp.rep1.r1
	1A and Suppl 1B	Wang_MCF7_chip__FOXA1_CTRL.50bp.rep2.r1	
	1A and Suppl 1B	Wang_MCF7_chip__FOXA1_CTRL.76bp.rep1.r1	Wang_MCF7_chip__INPUT_CTRL.76bp.rep1.r1
MCF7-HER2 FOXA1 ChIP-seq	1B and Suppl 1B	Wang_MCF7-HER2up_chip__FOXA1_CTRL.76bp.rep1.r1	Wang_MCF7-HER2up_chip__INPUT_CTRL.76bp.rep1.r1
	1B and Suppl 1B	Wang_MCF7-HER2up_chip__FOXA1_CTRL.76bp.rep2.r1	
Control FOXA1 ChIP-seq	4B and 4D	PDX_FOXA1__NaCl.rep1.r1	PDX__INPUT_NaCl.rep1.r1
	4B and 4D	PDX_FOXA1__NaCl.rep2.r1	PDX__INPUT_NaCl.rep2.r1
Fulvestrant FOXA1 ChIP-seq	4B and 4D	PDX_FOXA1__fulwestrant.rep1.r1	PDX__INPUT_fulwerstrant.rep1.r1
	4B and 4D	PDX_FOXA1__fulwestrant.rep2.r1	PDX__INPUT_fulwerstrant.rep2.r1
Heregulin and Transtuzumab FOXA1 ChIP-seq	4B and 4D	PDX_FOXA1__herceptin_fulwestrant.rep1.r1	PDX__INPUT_fulwerstrant.rep1.r1
	4B and 4D	PDX_FOXA1__herceptin_fulwestrant.rep2.r1	PDX__INPUT_fulwerstrant.rep2.r1
Heregulin FOXA1 ChIP-seq	4D	PDX_FOXA1__heregulin.rep1.r1	PDX__INPUT_NaCl.rep1.r1
	4D	PDX_FOXA1__heregulin.rep2.r1	PDX__INPUT_NaCl.rep2.r1
Heregulin and Fulvestrant FOXA1 ChIP-seq	4D	PDX_FOXA1__heregulin_fulwestrant.rep1.r1	PDX__INPUT_fulwerstrant.rep1.r1
	4D	PDX_FOXA1__heregulin_fulwestrant.rep2.r1	PDX__INPUT_fulwerstrant.rep2.r1
Heregulin, Fulvestrant and Transtuzumab FOXA1 ChIP-seq	4D	PDX_FOXA1__heregulin_herceptin_fulwestrant.rep1.r1	PDX__INPUT_fulwerstrant.rep1.r1
	4D	PDX_FOXA1__heregulin_herceptin_fulwestrant.rep2.r1	PDX__INPUT_fulwerstrant.rep2.r1

Legend: List of sequencing files. Column: 1 (name of FOXA1 ChIP condition), 2 (Figure with data is shown), 3 (name of the ChIP-sequencing files used to call the peaks; rows 1-3: cell lines and rows 4-9: PDXs tumors) and 4 (name of the input files used to call the peaks; rows 1-3: cell lines and rows 4-9: PDXs tumors).

Supplementary Table 2

QC of sequencing files

Sample Name	% Duplicates	% GC	M sequencing reads	Number of unique reads
**Wang_BT474_chip__FOXA1_CTRL.50bp.rep1.r1**	89.6%	35%	43.6M	472624000
**Wang_BT474_chip__FOXA1_CTRL.76bp.rep1.r1**	13.3%	40%	42.3M	3667410000
**Wang_BT474_chip__FOXA1_CTRL.76bp.rep2.r1**	17.2%	45%	38.1M	3154680000
**Wang_BT474_chip__FOXA1_CTRL.76bp.rep3.r1**	14.1%	41%	64.5M	5540550000
**Wang_BT474_chip__INPUT.50bp.rep1.r1**	96.6%	35%	43.9M	149260000
**Wang_BT474_chip__INPUT.76bp.rep1.r1**	9.5%	41%	40.2M	3638100000
**Wang_BT474_chip__INPUT.76bp.rep2.r1**	7.9%	45%	27.6M	3702420000
**Wang_MCF7-HER2up_chip__FOXA1_CTRL.76bp.rep1.r1**	10.5%	40%	41.8M	3597900000
**Wang_MCF7-HER2up_chip__FOXA1_CTRL.76bp.rep2.r1**	12.9%	43%	32.3M	3501420000
**Wang_MCF7-HER2up_chip__INPUT_CTRL.76bp.rep1.r1**	9.4%	41%	44.2M	3642120000
**Wang_MCF7_chip__FOXA1_CTRL.50bp.rep1.r1**	75.7%	39%	63.5M	976860000
**Wang_MCF7_chip__FOXA1_CTRL.50bp.rep2.r1**	19.8%	39%	57.7M	3224040000
**Wang_MCF7_chip__FOXA1_CTRL.76bp.rep1.r1**	10.4%	40%	41.8M	3601920000
**Wang_MCF7_chip__INPUT_CTRL.50bp.rep1.r1**	81.6%	36%	36.7M	739680000
**Wang_MCF7_chip__INPUT_CTRL.76bp.rep1.r1**	12.1%	41%	62.6M	3533580000
**Wang_MCF7_chip__INPUT_CTRL.76bp.rep2.r1**	6.9%	48%	32.8M	3742620000
**PDX_FOXA1__NaCl.rep1.r1**	16.9%	49%	39.3M	3315690000
**PDX_FOXA1__NaCl.rep2.r1**	44.7%	43%	34.7M	1918911919
**PDX_FOXA1__fulwestrant.rep1.r1**	12.1%	51%	23.5M	2065654131
**PDX_FOXA1__fulwestrant.rep2.r1**	46.6%	44%	53.5M	2856908571
**PDX_FOXA1__herceptin_fulwestrant.rep1.r1**	60.4%	52%	77.8M	3080892324
**PDX_FOXA1__herceptin_fulwestrant.rep2.r1**	42.5%	41%	39.3M	2259761299
**PDX_FOXA1__heregulin.rep1.r1**	39.4%	50%	63.5M	3848123089
**PDX_FOXA1__heregulin.rep2.r1**	17.9%	41%	34.4M	2824259770
**PDX_FOXA1__heregulin_fulwestrant.rep1.r1**	43.8%	49%	69.3M	3894691157
**PDX_FOXA1__heregulin_fulwestrant.rep2.r1**	37.4%	41%	26.4M	1652654874
**PDX_FOXA1__heregulin_herceptin_fulwestrant.rep1.r1**	47.7%	54%	66.8M	3493674936
**PDX_FOXA1__heregulin_herceptin_fulwestrant.rep2.r1**	26.5%	48%	36.6M	2668079349
**PDX__INPUT_NaCl.rep1.r1**	24.7%	51%	76.8M	5783120963
**PDX__INPUT_NaCl.rep2.r1**	29.3%	45%	53.8M	3803717055
**PDX__INPUT_fulwerstrant.rep1.r1**	21.5%	50%	77.5M	6083847340
**PDX__INPUT_fulwerstrant.rep2.r1**	32.9%	47%	51.2M	3435578404

Legend: QC list of sequencing files. Column: 1 (name of the sequencing file), 2 (% of duplicates), 3 (% of GC), 4 (millions of sequencing reads) and 5 (millions of unique sequencing reads).

Supplementary Table 3

Motif analysis of MCF-7 and BT474 shared FOXA1 sites (59,904) compared to background regions (246,052)

Rank	Motif Name	Consensus	P-value	FOXA1 regions (%)	Background regions (%)
1	FOXA1(Forkhead)/MCF7-FOXA1-ChIP-Seq(GSE26831)/Homer	WAAGTAAACA	1e-13461	79.84%	25.35%
2	FOXA1(Forkhead)/LNCAP-FOXA1-ChIP-Seq(GSE27824)/Homer	WAAGTAAACA	1e-13224	84.25%	30.22%
3	Foxa2(Forkhead)/Liver-Foxa2-ChIP-Seq(GSE25694)/Homer	CYTGTTTACWYW	1e-12724	73.14%	20.82%
4	FOXM1(Forkhead)/MCF7-FOXM1-ChIP-Seq(GSE72977)/Homer	TRTTTACTTW	1e-12572	78.13%	25.40%
5	Fox:Ebox(Forkhead,bHLH)/Panc1-Foxa2-ChIP-Seq(GSE47459)/Homer	NNNVCTGWGYAAACASN	1e-11957	67.58%	17.81%
6	Foxa3(Forkhead)/Liver-Foxa3-ChIP-Seq(GSE77670)/Homer	BSNTGTTTACWYWGN	1e-11280	52.21%	9.14%
7	FoxL2(Forkhead)/Ovary-FoxL2-ChIP-Seq(GSE60858)/Homer	WWTRTAAACAVG	1e-7569	66.89%	25.93%
8	FOXK2(Forkhead)/U2OS-FOXK2-ChIP-Seq(E-MTAB-2204)/Homer	SCHTGTTTACAT	1e-6901	53.09%	16.65%
9	FoxD3(forkhead)/ZebrafishEmbryo-Foxd3.biotin-ChIP-seq(GSE106676)/Homer	TGTTTAYTTAGC	1e-6607	56.32%	19.64%
10	FOXK1(Forkhead)/HEK293-FOXK1-ChIP-Seq(GSE51673)/Homer	NVWTGTTTAC	1e-6531	68.97%	30.47%
11	Foxf1(Forkhead)/Lung-Foxf1-ChIP-Seq(GSE77951)/Homer	WWATRTAAACAN	1e-6485	66.85%	28.65%
12	Foxo3(Forkhead)/U2OS-Foxo3-ChIP-Seq(E-MTAB-2701)/Homer	DGTAAACA	1e-6059	57.76%	22.06%
13	PHA-4(Forkhead)/cElegans-Embryos-PHA4-ChIP-Seq(modEncode)/Homer	KTGTTTGC	1e-5786	93.05%	61.92%
14	Foxo1(Forkhead)/RAW-Foxo1-ChIP-Seq(Fan_et_al.)/Homer	CTGTTTAC	1e-5725	69.50%	33.29%
15	FOXP1(Forkhead)/H9-FOXP1-ChIP-Seq(GSE31006)/Homer	NYYTGTTTACHN	1e-5443	43.02%	12.89%
16	AP-2gamma(AP2)/MCF7-TFAP2C-ChIP-Seq(GSE21234)/Homer	SCCTSAGGSCAW	1e-2995	24.92%	7.02%
17	AP-2alpha(AP2)/Hela-AP2alpha-ChIP-Seq(GSE31477)/Homer	ATGCCCTGAGGC	1e-2751	20.41%	5.03%
18	VRN1(ABI3VP1)/col-VRN1-DAP-Seq(GSE60143)/Homer	TTTTTTTTTT	1e-2698	11.95%	1.37%
19	GAGA-repeat/SacCer-Promoters/Homer	CTYTCTYTCTCTCTC	1e-2669	77.87%	53.92%
20	RLR1?/SacCer-Promoters/Homer	WTTTTCYYTTTT	1e-2665	27.80%	9.51%
21	ZNF416(Zf)/HEK293-ZNF416.GFP-ChIP-Seq(GSE58341)/Homer	WDNCTGGGCA	1e-2657	27.56%	9.38%
22	SCL(bHLH)/HPC7-Scl-ChIP-Seq(GSE13511)/Homer	AVCAGCTG	1e-2563	70.69%	46.46%
23	Ptf1a(bHLH)/Panc1-Ptf1a-ChIP-Seq(GSE47459)/Homer	ACAGCTGTTN	1e-2175	48.39%	26.89%
24	E2A(bHLH)/proBcell-E2A-ChIP-Seq(GSE21978)/Homer	DNRCAGCTGY	1e-2138	28.64%	11.56%
25	hTCT(CPE)	BCKTTCTYTTY	1e-2136	53.11%	31.27%
26	GAGA-repeat/Arabidopsis-Promoters/Homer	CTCTCTCTCY	1e-2094	25.85%	9.81%
27	SeqBias: GA-repeat	GAGAGAGAGA	1e-2088	88.64%	70.43%
28	EBF1(EBF)/Near-E2A-ChIP-Seq(GSE21512)/Homer	GTCCCCWGGGGA	1e-2018	19.10%	5.82%
29	HEB(bHLH)/mES-Heb-ChIP-Seq(GSE53233)/Homer	VCAGCTGBNN	1e-1984	35.73%	17.30%
30	Ascl1(bHLH)/NeuralTubes-Ascl1-ChIP-Seq(GSE55840)/Homer	NNVVCAGCTGBN	1e-1962	29.53%	12.76%
31	NF1-halfsite(CTF)/LNCaP-NF1-ChIP-Seq(Unpublished)/Homer	YTGCCAAG	1e-1945	39.54%	20.46%
32	Bcl6(Zf)/Liver-Bcl6-ChIP-Seq(GSE31578)/Homer	NNNCTTTCCAGGAAA	1e-1912	33.51%	15.88%
33	GLIS3(Zf)/Thyroid-Glis3.GFP-ChIP-Seq(GSE103297)/Homer	CTCCCTGGGAGGCCN	1e-1865	24.99%	9.93%
34	NF1(CTF)/LNCAP-NF1-ChIP-Seq(Unpublished)/Homer	CYTGGCABNSTGCCAR	1e-1810	11.69%	2.36%
35	Erra(NR)/HepG2-Erra-ChIP-Seq(GSE31477)/Homer	CAAAGGTCAG	1e-1797	41.71%	22.92%
36	TF3A(C2H2)/col-TF3A-DAP-Seq(GSE60143)/Homer	NNDDGAGGAGGWNNN	1e-1764	27.46%	11.97%
37	Ap4(bHLH)/AML-Tfap4-ChIP-Seq(GSE45738)/Homer	NAHCAGCTGD	1e-1747	24.62%	10.05%
38	PU.1-IRF(ETS:IRF)/Bcell-PU.1-ChIP-Seq(GSE21512)/Homer	MGGAAGTGAAAC	1e-1711	31.43%	15.09%
39	ERG(ETS)/VCaP-ERG-ChIP-Seq(GSE14097)/Homer	ACAGGAAGTG	1e-1697	34.13%	17.23%
40	Tcf12(bHLH)/GM12878-Tcf12-ChIP-Seq(GSE32465)/Homer	VCAGCTGYTG	1e-1660	18.37%	6.27%
41	Tlx?(NR)/NPC-H3K4me1-ChIP-Seq(GSE16256)/Homer	CTGGCAGSCTGCCA	1e-1648	10.72%	2.17%
42	SeqBias: CA-repeat	CACACACACA	1e-1570	84.38%	67.72%
43	Jun-AP1(bZIP)/K562-cJun-ChIP-Seq(GSE31477)/Homer	GATGASTCATCN	1e-1536	11.34%	2.65%
44	Fosl2(bZIP)/3T3L1-Fosl2-ChIP-Seq(GSE56872)/Homer	NATGASTCABNN	1e-1519	14.73%	4.48%
45	SpliceAcceptor/Homer	TYYYYRCAGG	1e-1506	83.03%	66.44%
46	NFATC2(RHD)/Islets-NFATC2-ChIP-Seq(GSE158496)/Homer	WTTTTCCATTGS	1e-1505	50.57%	32.24%
47	RXR(NR),DR1/3T3L1-RXR-ChIP-Seq(GSE13511)/Homer	TAGGGCAAAGGTCA	1e-1494	22.80%	9.65%
48	Ascl2(bHLH)/ESC-Ascl2-ChIP-Seq(GSE97712)/Homer	SSRGCAGCTGCH	1e-1481	21.93%	9.10%
49	E2A(bHLH),near_PU.1/Bcell-PU.1-ChIP-Seq(GSE21512)/Homer	NVCACCTGBN	1e-1465	29.19%	14.40%
50	MyoG(bHLH)/C2C12-MyoG-ChIP-Seq(GSE36024)/Homer	AACAGCTG	1e-1435	20.49%	8.29%
51	Tcf21(bHLH)/ArterySmoothMuscle-Tcf21-ChIP-Seq(GSE61369)/Homer	NAACAGCTGG	1e-1430	19.79%	7.84%
52	ZNF148(Zf)/MDAMB231-ZNF148-ChIP-Seq(GSE147020)/Homer	CCCCTCCCCCAC	1e-1408	8.95%	1.74%
53	KLF14(Zf)/HEK293-KLF14.GFP-ChIP-Seq(GSE58341)/Homer	RGKGGGCGKGGC	1e-1402	24.05%	10.87%
54	Rbpj1(?)/Panc1-Rbpj1-ChIP-Seq(GSE47459)/Homer	HTTTCCCASG	1e-1372	31.89%	16.92%
55	Znf263(Zf)/K562-Znf263-ChIP-Seq(GSE31477)/Homer	CVGTSCTCCC	1e-1368	28.78%	14.52%
56	AT5G60130(ABI3VP1)/col-AT5G60130-DAP-Seq(GSE60143)/Homer	WTTYTAAGVAAA	1e-1349	61.00%	43.23%
57	Fra2(bZIP)/Striatum-Fra2-ChIP-Seq(GSE43429)/Homer	GGATGACTCATC	1e-1348	20.48%	8.58%
58	EBF2(EBF)/BrownAdipose-EBF2-ChIP-Seq(GSE97114)/Homer	NABTCCCWDGGGAVH	1e-1319	18.72%	7.49%
59	MyoD(bHLH)/Myotube-MyoD-ChIP-Seq(GSE21614)/Homer	RRCAGCTGYTSY	1e-1304	14.35%	4.79%
60	HIC1(Zf)/Treg-ZBTB29-ChIP-Seq(GSE99889)/Homer	TGCCAGCB	1e-1300	38.39%	22.62%
61	NGA4(ABI3VP1)/col-NGA4-DAP-Seq(GSE60143)/Homer	TKNTCAGGTG	1e-1291	51.85%	34.72%
62	GRHL2(CP2)/HBE-GRHL2-ChIP-Seq(GSE46194)/Homer	AAACYKGTTWDACMRGTTTB	1e-1263	17.56%	6.92%
63	Twist2(bHLH)/Myoblast-Twist2.Ty1-ChIP-Seq(GSE127998)/Homer	MCAGCTGBYH	1e-1247	35.40%	20.39%
64	JunB(bZIP)/DendriticCells-Junb-ChIP-Seq(GSE36099)/Homer	RATGASTCAT	1e-1244	23.16%	10.85%
65	REM19(REM)/colamp-REM19-DAP-Seq(GSE60143)/Homer	AAAAAAAA	1e-1244	29.77%	15.85%
66	BHLHA15(bHLH)/NIH3T3-BHLHB8.HA-ChIP-Seq(GSE119782)/Homer	NAMCAGCTGK	1e-1235	29.13%	15.39%
67	TCF4(bHLH)/SHSY5Y-TCF4-ChIP-Seq(GSE96915)/Homer	SMCATCTGKH	1e-1224	32.17%	17.86%
68	Maz(Zf)/HepG2-Maz-ChIP-Seq(GSE31477)/Homer	GGGGGGGG	1e-1222	16.24%	6.19%
69	AR-halfsite(NR)/LNCaP-AR-ChIP-Seq(GSE27824)/Homer	CCAGGAACAG	1e-1217	63.92%	47.09%
70	AP-1(bZIP)/ThioMac-PU.1-ChIP-Seq(GSE21512)/Homer	VTGACTCATC	1e-1210	27.80%	14.47%
71	EHF(ETS)/LoVo-EHF-ChIP-Seq(GSE49402)/Homer	AVCAGGAAGT	1e-1206	31.77%	17.63%
72	BPC1(BBRBPC)/colamp-BPC1-DAP-Seq(GSE60143)/Homer	GARGAGAGAGAA	1e-1201	16.83%	6.64%
73	Atoh7(bHLH)/Retina-Atoh7-CutnRun(GSE156756)/Homer	KRRCAGCTGGTS	1e-1199	15.82%	5.99%
74	PPARE(NR),DR1/3T3L1-Pparg-ChIP-Seq(GSE13511)/Homer	TGACCTTTGCCCCA	1e-1192	20.30%	9.01%
75	MYRF(MYRF)/CFPAC1-MYRF-ChIP-Seq(GSE145627)/Homer	AGTGCCTGGCAC	1e-1171	10.44%	2.90%
76	NeuroG2(bHLH)/Fibroblast-NeuroG2-ChIP-Seq(GSE75910)/Homer	ACCATCTGTT	1e-1171	33.33%	19.09%
77	HuR(?)/HEK293-HuR-CLIP-Seq(GSE87887)/Homer	BTTTGGTTTG	1e-1159	84.45%	70.44%
78	Unknown-ESC-element(?)/mES-Nanog-ChIP-Seq(GSE11724)/Homer	CACAGCAGGGGG	1e-1156	11.01%	3.24%
79	BATF(bZIP)/Th17-BATF-ChIP-Seq(GSE39756)/Homer	DATGASTCAT	1e-1154	26.52%	13.75%
80	Atoh1(bHLH)/Cerebellum-Atoh1-ChIP-Seq(GSE22111)/Homer	VNRVCAGCTGGY	1e-1142	22.23%	10.59%
81	IDD5(C2H2)/colamp-IDD5-DAP-Seq(GSE60143)/Homer	TTTTGTCTTTTTBTK	1e-1135	29.42%	16.11%
82	FRS9(ND)/col-FRS9-DAP-Seq(GSE60143)/Homer	RGAGAGAGAAAG	1e-1132	6.76%	1.20%
83	Atf3(bZIP)/GBM-ATF3-ChIP-Seq(GSE33912)/Homer	DATGASTCATHN	1e-1129	26.57%	13.91%
84	ELF3(ETS)/PDAC-ELF3-ChIP-Seq(GSE64557)/Homer	ANCAGGAAGT	1e-1115	20.19%	9.23%
85	ZEB1(Zf)/PDAC-ZEB1-ChIP-Seq(GSE64557)/Homer	VCAGGTRDRY	1e-1098	36.88%	22.48%
86	Etv2(ETS)/ES-ER71-ChIP-Seq(GSE59402)/Homer	NNAYTTCCTGHN	1e-1094	21.38%	10.17%
87	PPARa(NR),DR1/Liver-Ppara-ChIP-Seq(GSE47954)/Homer	VNAGGKCAAAGGTCA	1e-1090	23.68%	11.89%
88	NeuroD1(bHLH)/Islet-NeuroD1-ChIP-Seq(GSE30298)/Homer	GCCATCTGTT	1e-1086	17.64%	7.57%
89	Trl(Zf)/S2-GAGAfactor-ChIP-Seq(GSE40646)/Homer	RGAGAGAG	1e-1084	45.34%	30.05%
90	ZNF467(Zf)/HEK293-ZNF467.GFP-ChIP-Seq(GSE58341)/Homer	TGGGGAAGGGCM	1e-1083	13.50%	4.89%
91	EWS:ERG-fusion(ETS)/CADO_ES1-EWS:ERG-ChIP-Seq(SRA014231)/Homer	ATTTCCTGTN	1e-1076	20.39%	9.53%
92	Myf5(bHLH)/GM-Myf5-ChIP-Seq(GSE24852)/Homer	BAACAGCTGT	1e-1070	14.33%	5.45%
93	Bcl11a(Zf)/HSPC-BCL11A-ChIP-Seq(GSE104676)/Homer	TYTGACCASWRG	1e-1060	17.53%	7.59%
94	Tal1	CATCTG	1e-1060	32.83%	19.29%
95	KLF5(Zf)/LoVo-KLF5-ChIP-Seq(GSE49402)/Homer	DGGGYGKGGC	1e-1055	20.50%	9.70%
96	Snail1(Zf)/LS174T-SNAIL1.HA-ChIP-Seq(GSE127183)/Homer	TRCACCTGCY	1e-1051	19.04%	8.67%
97	Fra1(bZIP)/BT549-Fra1-ChIP-Seq(GSE46166)/Homer	NNATGASTCATH	1e-1042	23.98%	12.34%
98	CTCF(Zf)/CD4+-CTCF-ChIP-Seq(Barski_et_al.)/Homer	AYAGTGCCMYCTRGTGGCCA	1e-1035	4.55%	0.49%
99	KLF10(Zf)/HEK293-KLF10.GFP-ChIP-Seq(GSE58341)/Homer	GGGGGTGTGTCC	1e-1030	10.89%	3.48%
100	Sp5(Zf)/mES-Sp5.Flag-ChIP-Seq(GSE72989)/Homer	RGKGGGCGGAGC	1e-1010	14.27%	5.60%
101	Fos(bZIP)/TSC-Fos-ChIP-Seq(GSE110950)/Homer	NDATGASTCAYN	1e-1010	25.07%	13.32%
102	KLF1(Zf)/HUDEP2-KLF1-CutnRun(GSE136251)/Homer	VDGGGYGGGGCY	1e-1000	13.89%	5.39%
103	TEAD(TEA)/Fibroblast-PU.1-ChIP-Seq(Unpublished)/Homer	YCWGGAATGY	1e-993	23.24%	12.00%
104	PR(NR)/T47D-PR-ChIP-Seq(GSE31130)/Homer	VAGRACAKNCTGTBC	1e-980	48.36%	33.56%
105	Zic3(Zf)/mES-Zic3-ChIP-Seq(GSE37889)/Homer	GGCCYCCTGCTGDGH	1e-960	9.47%	2.85%
106	bZIP18(bZIP)/colamp-bZIP18-DAP-Seq(GSE60143)/Homer	KGMCAGCTND	1e-945	81.01%	67.84%
107	Olig2(bHLH)/Neuron-Olig2-ChIP-Seq(GSE30882)/Homer	RCCATMTGTT	1e-937	43.23%	29.13%
108	BORIS(Zf)/K562-CTCFL-ChIP-Seq(GSE32465)/Homer	CNNBRGCGCCCCCTGSTGGC	1e-934	4.63%	0.62%
109	ZNF322(Zf)/HEK293-ZNF322.GFP-ChIP-Seq(GSE58341)/Homer	GAGCCTGGTACTGWGCCTGR	1e-933	5.41%	0.92%
110	ZFX(Zf)/mES-Zfx-ChIP-Seq(GSE11431)/Homer	AGGCCTRG	1e-928	21.76%	11.19%
111	THRb(NR)/Liver-NR1A2-ChIP-Seq(GSE52613)/Homer	TRAGGTCA	1e-928	67.85%	53.36%
112	TEAD4(TEA)/Tropoblast-Tead4-ChIP-Seq(GSE37350)/Homer	CCWGGAATGY	1e-916	25.20%	13.91%
113	RARa(NR)/K562-RARa-ChIP-Seq(Encode)/Homer	TTGAMCTTTG	1e-908	58.76%	44.18%
114	IDD4(C2H2)/col-IDD4-DAP-Seq(GSE60143)/Homer	TTTGTCTTTWTB	1e-891	34.57%	21.79%
115	STAT4(Stat)/CD4-Stat4-ChIP-Seq(GSE22104)/Homer	NYTTCCWGGAAR	1e-891	26.81%	15.33%
116	REF6(Zf)/Arabidopsis-REF6-ChIP-Seq(GSE106942)/Homer	TVCTCTGTTT	1e-890	16.75%	7.71%
117	Tgif2(Homeobox)/mES-Tgif2-ChIP-Seq(GSE55404)/Homer	TGTCANYT	1e-875	68.65%	54.64%
118	TEAD2(TEA)/Py2T-Tead2-ChIP-Seq(GSE55709)/Homer	CCWGGAATGY	1e-854	16.78%	7.88%
119	Sox10(HMG)/SciaticNerve-Sox3-ChIP-Seq(GSE35132)/Homer	CCWTTGTYYB	1e-849	44.26%	30.72%
120	Bach2(bZIP)/OCILy7-Bach2-ChIP-Seq(GSE44420)/Homer	TGCTGAGTCA	1e-839	7.79%	2.21%
121	Tgif1(Homeobox)/mES-Tgif1-ChIP-Seq(GSE55404)/Homer	YTGWCADY	1e-836	66.60%	52.80%
122	Unknown6/Drosophila-Promoters/Homer	AATTTTAAAA	1e-826	34.26%	21.95%
123	ZEB2(Zf)/SNU398-ZEB2-ChIP-Seq(GSE103048)/Homer	GNMCAGGTGTGC	1e-820	20.10%	10.46%
124	Sox4(HMG)/proB-Sox4-ChIP-Seq(GSE50066)/Homer	YCTTTGTTCC	1e-818	25.61%	14.78%
125	EAR2(NR)/K562-NR2F6-ChIP-Seq(Encode)/Homer	NRBCARRGGTCA	1e-800	28.21%	17.00%
126	KLF6(Zf)/PDAC-KLF6-ChIP-Seq(GSE64557)/Homer	MKGGGYGTGGCC	1e-800	16.20%	7.70%
127	MRE(NR)/Neuro2A-NR3C2-ChIPnexus(GSE115417)/Homer	GGAACAGAVTGTCCT	1e-788	28.81%	17.58%
128	RBPJ:Ebox(?,bHLH)/Panc1-Rbpj1-ChIP-Seq(GSE47459)/Homer	GGGRAARRGRMCAGMTG	1e-785	6.66%	1.74%
129	COUP-TFII(NR)/K562-NR2F1-ChIP-Seq(Encode)/Homer	GKBCARAGGTCA	1e-780	31.54%	19.93%
130	Sp2(Zf)/HEK293-Sp2.eGFP-ChIP-Seq(Encode)/Homer	YGGCCCCGCCCC	1e-765	20.96%	11.40%
131	Zac1(Zf)/Neuro2A-Plagl1-ChIP-Seq(GSE75942)/Homer	HAWGRGGCCM	1e-754	41.90%	29.29%
132	Unknown2/Drosophila-Promoters/Homer	CATCMCTA	1e-743	26.98%	16.35%
133	Zfp281(Zf)/ES-Zfp281-ChIP-Seq(GSE81042)/Homer	CCCCTCCCCCAC	1e-734	2.94%	0.25%
134	Stat3+il21(Stat)/CD4-Stat3-ChIP-Seq(GSE19198)/Homer	SVYTTCCNGGAARB	1e-721	17.69%	9.15%
135	TEAD1(TEAD)/HepG2-TEAD1-ChIP-Seq(Encode)/Homer	CYRCATTCCA	1e-720	29.74%	18.80%
136	Slug(Zf)/Mesoderm-Snai2-ChIP-Seq(GSE61475)/Homer	SNGCACCTGCHS	1e-713	11.56%	4.85%
137	MafB(bZIP)/BMM-Mafb-ChIP-Seq(GSE75722)/Homer	WNTGCTGASTCAGCANWTTY	1e-711	12.03%	5.17%
138	bZIP52(bZIP)/colamp-bZIP52-DAP-Seq(GSE60143)/Homer	NDNHCAGCTGTCANN	1e-706	33.87%	22.47%
139	KLF3(Zf)/MEF-Klf3-ChIP-Seq(GSE44748)/Homer	NRGCCCCRCCCHBNN	1e-700	8.20%	2.79%
140	ETS1(ETS)/Jurkat-ETS1-ChIP-Seq(GSE17954)/Homer	ACAGGAAGTG	1e-697	22.39%	12.90%
141	SOX1(HMG)/NPC-SOX1-ChIP-Seq(GSE138215)/Homer	CCATTGTTCB	1e-684	54.34%	41.71%
142	Klf15(Zf)/Liver-Klf15-ChIP-Seq(GSE166083)/Homer	RGGGGGCGGGGC	1e-681	15.83%	7.96%
143	THRb(NR)/HepG2-THRb.Flag-ChIP-Seq(Encode)/Homer	GGTCACCTGAGGTCA	1e-677	10.84%	4.50%
144	Klf4(Zf)/mES-Klf4-ChIP-Seq(GSE11431)/Homer	GCCACACCCA	1e-669	6.96%	2.16%
145	ZNF91(Zf)/HEK-ZNF91.HA-ChIP-Seq(GSE162571)/Homer	RGCMGCCTYC	1e-661	12.74%	5.85%
146	PU.1(ETS)/ThioMac-PU.1-ChIP-Seq(GSE21512)/Homer	AGAGGAAGTG	1e-660	11.53%	5.03%
147	Smad3(MAD)/NPC-Smad3-ChIP-Seq(GSE36673)/Homer	TWGTCTGV	1e-650	54.14%	41.82%
148	TEAD3(TEA)/HepG2-TEAD3-ChIP-Seq(Encode)/Homer	TRCATTCCAG	1e-640	34.62%	23.64%
149	NF1:FOXA1(CTF,Forkhead)/LNCAP-FOXA1-ChIP-Seq(GSE27824)/Homer	WNTGTTTRYTTTGGCA	1e-639	4.30%	0.88%
150	Sox21(HMG)/ESC-SOX21-ChIP-Seq(GSE110505)/Homer	BCCWTTGTBYKV	1e-634	45.99%	34.11%
151	Hand2(bHLH)/Mesoderm-Hand2-ChIP-Seq(GSE61475)/Homer	TGACANARRCCAGRC	1e-630	10.55%	4.49%
152	ZBTB9(Zf)/HEK293T-ZBTB9-ChIP-Seq(GSE251687)/Homer	RGCCACGDRR	1e-630	35.67%	24.65%
153	ZBTB18(Zf)/HEK293-ZBTB18.GFP-ChIP-Seq(GSE58341)/Homer	AACATCTGGA	1e-622	10.78%	4.67%
154	Gfi1b(Zf)/HPC7-Gfi1b-ChIP-Seq(GSE22178)/Homer	MAATCACTGC	1e-618	18.10%	10.00%
155	Klf9(Zf)/GBM-Klf9-ChIP-Seq(GSE62211)/Homer	GCCACRCCCACY	1e-618	5.50%	1.49%
156	COUP-TFII(NR)/Artia-Nr2f2-ChIP-Seq(GSE46497)/Homer	AGRGGTCA	1e-613	35.21%	24.37%
157	Sox3(HMG)/NPC-Sox3-ChIP-Seq(GSE33059)/Homer	CCWTTGTY	1e-613	47.77%	36.00%
158	HLH-1(bHLH)/cElegans-Embryo-HLH1-ChIP-Seq(modEncode)/Homer	RACAGCTGTTBH	1e-612	19.82%	11.38%
159	RAV1(RAV)/colamp-RAV1-DAP-Seq(GSE60143)/Homer	TWWTTTCTGTTG	1e-612	29.56%	19.43%
160	Elf4(ETS)/BMDM-Elf4-ChIP-Seq(GSE88699)/Homer	ACTTCCKGKT	1e-604	22.63%	13.68%
161	RUNX-AML(Runt)/CD4+-PolII-ChIP-Seq(Barski_et_al.)/Homer	GCTGTGGTTW	1e-603	17.91%	9.93%
162	MafA(bZIP)/Islet-MafA-ChIP-Seq(GSE30298)/Homer	TGCTGACTCA	1e-600	18.21%	10.18%
163	ZNF189(Zf)/HEK293-ZNF189.GFP-ChIP-Seq(GSE58341)/Homer	TGGAACAGMA	1e-600	16.98%	9.24%
164	Tbx5(T-box)/HL1-Tbx5.biotin-ChIP-Seq(GSE21529)/Homer	AGGTGTCA	1e-597	59.95%	48.14%
165	Sox15(HMG)/CPA-Sox15-ChIP-Seq(GSE62909)/Homer	RAACAATGGN	1e-592	33.11%	22.68%
166	ZNF711(Zf)/SHSY5Y-ZNF711-ChIP-Seq(GSE20673)/Homer	AGGCCTAG	1e-592	28.06%	18.30%
167	ELF5(ETS)/T47D-ELF5-ChIP-Seq(GSE30407)/Homer	ACVAGGAAGT	1e-588	19.75%	11.46%
168	WT1(Zf)/Kidney-WT1-ChIP-Seq(GSE90016)/Homer	MCTCCCMCRCAB	1e-581	9.35%	3.88%
169	PABPC1(?)/MEL-PABC1-CLIP-Seq(GSE69755)/Homer	HAATAAAGNN	1e-580	64.15%	52.61%
170	polyAsite/CPSF/Homer	TTCAATAAAG	1e-571	66.06%	54.69%
171	SPDEF(ETS)/VCaP-SPDEF-ChIP-Seq(SRA014231)/Homer	ASWTCCTGBT	1e-560	24.52%	15.54%
172	Zic(Zf)/Cerebellum-ZIC1.2-ChIP-Seq(GSE60731)/Homer	CCTGCTGAGH	1e-556	15.52%	8.37%
173	Nr5a2(NR)/Pancreas-LRH1-ChIP-Seq(GSE34295)/Homer	BTCAAGGTCA	1e-541	15.04%	8.09%
174	Meis1(Homeobox)/MastCells-Meis1-ChIP-Seq(GSE48085)/Homer	VGCTGWCAVB	1e-540	35.31%	25.10%
175	ETV1(ETS)/GIST48-ETV1-ChIP-Seq(GSE22441)/Homer	AACCGGAAGT	1e-538	28.61%	19.19%
176	PRDM1(Zf)/Hela-PRDM1-ChIP-Seq(GSE31477)/Homer	ACTTTCACTTTC	1e-537	15.90%	8.76%
177	EKLF(Zf)/Erythrocyte-Klf1-ChIP-Seq(GSE20478)/Homer	NWGGGTGTGGCY	1e-537	4.55%	1.17%
178	HNF4a(NR),DR1/HepG2-HNF4a-ChIP-Seq(GSE25021)/Homer	CARRGKBCAAAGTYCA	1e-536	10.58%	4.88%
179	Mesp1(bHLH)/ESC-Mesp1-ChIP-Seq(GSE165102)/Homer	RCCATTTGBY	1e-535	16.18%	8.99%
180	THRa(NR)/C17.2-THRa-ChIP-Seq(GSE38347)/Homer	GGTCANYTGAGGWCA	1e-532	8.98%	3.81%
181	bZIP:IRF(bZIP,IRF)/Th17-BatF-ChIP-Seq(GSE39756)/Homer	NAGTTTCABTHTGACTNW	1e-523	14.94%	8.10%
182	GATA(Zf),IR3/iTreg-Gata3-ChIP-Seq(GSE20898)/Homer	NNNNNBAGATAWYATCTVHN	1e-523	9.90%	4.46%
183	Zic2(Zf)/ESC-Zic2-ChIP-Seq(SRP197560)/Homer	CHCAGCRGGRGG	1e-521	7.17%	2.69%
184	Sox9(HMG)/Limb-SOX9-ChIP-Seq(GSE73225)/Homer	AGGVNCCTTTGT	1e-521	22.51%	14.15%
185	STAT5(Stat)/mCD4+-Stat5-ChIP-Seq(GSE12346)/Homer	RTTTCTNAGAAA	1e-514	9.95%	4.53%
186	ERRg(NR)/Kidney-ESRRG-ChIP-Seq(GSE104905)/Homer	GTGACCTTGRVN	1e-511	18.63%	11.05%
187	Oct4(POU,Homeobox)/mES-Oct4-ChIP-Seq(GSE11431)/Homer	ATTTGCATAW	1e-511	20.64%	12.68%
188	SF1(NR)/H295R-Nr5a1-ChIP-Seq(GSE44220)/Homer	CAAGGHCANV	1e-507	9.63%	4.35%
189	Rfx6(HTH)/Min6b1-Rfx6.HA-ChIP-Seq(GSE62844)/Homer	TGTTKCCTAGCAACM	1e-505	21.34%	13.30%
190	FOXA1:AR(Forkhead,NR)/LNCAP-AR-ChIP-Seq(GSE27824)/Homer	AGTAAACAAAAAAGAACAND	1e-504	4.56%	1.25%
191	AT5G25475(ABI3VP1)/col-AT5G25475-DAP-Seq(GSE60143)/Homer	RNNRNCAAGCADNDB	1e-493	29.46%	20.30%
192	ZNF143|STAF(Zf)/CUTLL-ZNF143-ChIP-Seq(GSE29600)/Homer	ATTTCCCAGVAKSCY	1e-492	5.85%	1.99%
193	MafK(bZIP)/C2C12-MafK-ChIP-Seq(GSE36030)/Homer	GCTGASTCAGCA	1e-486	5.68%	1.91%
194	Oct2(POU,Homeobox)/Bcell-Oct2-ChIP-Seq(GSE21512)/Homer	ATATGCAAAT	1e-470	13.85%	7.58%
195	PRDM15(Zf)/ESC-Prdm15-ChIP-Seq(GSE73694)/Homer	YCCDNTCCAGGTTTT	1e-465	22.99%	14.97%
196	Pax8(Paired,Homeobox)/Thyroid-Pax8-ChIP-Seq(GSE26938)/Homer	GTCATGCHTGRCTGS	1e-465	5.98%	2.15%
197	Ets1-distal(ETS)/CD4+-PolII-ChIP-Seq(Barski_et_al.)/Homer	MACAGGAAGT	1e-459	7.54%	3.14%
198	AT5G02460(C2C2dof)/col-AT5G02460-DAP-Seq(GSE60143)/Homer	CHCCTTTT	1e-454	59.18%	48.88%
199	Nr5a2(NR)/mES-Nr5a2-ChIP-Seq(GSE19019)/Homer	BTCAAGGTCA	1e-454	10.85%	5.44%
200	NFAT(RHD)/Jurkat-NFATC1-ChIP-Seq(Jolma_et_al.)/Homer	ATTTTCCATT	1e-444	25.66%	17.42%
201	Esrrb(NR)/mES-Esrrb-ChIP-Seq(GSE11431)/Homer	KTGACCTTGA	1e-444	15.49%	9.00%
202	RUNX1(Runt)/Jurkat-RUNX1-ChIP-Seq(GSE29180)/Homer	AAACCACARM	1e-439	24.80%	16.72%
203	hINR(CPE)	SBCABW	1e-435	22.12%	14.46%
204	Stat3(Stat)/mES-Stat3-ChIP-Seq(GSE11431)/Homer	CTTCCGGGAA	1e-431	11.90%	6.33%
205	ZNF7(Zf)/HepG2-ZNF7.Flag-ChIP-Seq(Encode)/Homer	CTGCCWVCTTTTRTA	1e-430	17.71%	10.86%
206	RUNX2(Runt)/PCa-RUNX2-ChIP-Seq(GSE33889)/Homer	NWAACCACADNN	1e-429	20.54%	13.19%
207	DAG2(C2C2dof)/col-DAG2-DAP-Seq(GSE60143)/Homer	WWTTHACTTTTT	1e-428	57.82%	47.81%
208	IRF3(IRF)/BMDM-Irf3-ChIP-Seq(GSE67343)/Homer	AGTTTCAKTTTC	1e-412	10.25%	5.21%

Legend: The complete list of motifs for shared chromatin FOXA1 regions between MCF-7 and BT474 cells. The analysis with a p-value threshold of < 1e-100, identifies a total of 415 significant motifs). Among these, the Forkhead (FKH) motif ranks as one of the top motifs (p-value < 1e-13224), with approximately 79% of the shared chromatin FOXA1 regions containing a FKH motif. The ERE motif (p-value < 1e-301), accounting for 5.5% of these regions.

Supplementary Table 3: continuation

Motif analysis of MCF-7 and BT474 shared FOXA1 sites (59,904) compared to background regions (246,052)

Rank	Motif Name	Consensus	P-value	FOXA1 regions (%)	Background regions (%)
209	GRF9(GRF)/colamp-GRF9-DAP-Seq(GSE60143)/Homer	NWCTGACANNNNNNN	1e-412	23.37%	15.72%
210	STAT6(Stat)/Macrophage-Stat6-ChIP-Seq(GSE38377)/Homer	TTCCKNAGAA	1e-407	15.28%	9.07%
211	AZF1(C2H2)/colamp-AZF1-DAP-Seq(GSE60143)/Homer	DKSWCACT	1e-407	83.98%	76.07%
212	E-box/Drosophila-Promoters/Homer	AACAGCTGTTHN	1e-398	6.26%	2.54%
213	Oct11(POU,Homeobox)/NCIH1048-POU2F3-ChIP-seq(GSE115123)/Homer	GATTTGCATA	1e-398	14.72%	8.69%
214	NLP7(RWPRK)/col-NLP7-DAP-Seq(GSE60143)/Homer	TGRCCYTTCR	1e-396	40.10%	30.95%
215	BPC6(BBRBPC)/col-BPC6-DAP-Seq(GSE60143)/Homer	YTYTCTCTCTCTCTA	1e-394	1.67%	0.16%
216	STZ(C2H2)/colamp-STZ-DAP-Seq(GSE60143)/Homer	HNBTCACT	1e-386	89.87%	83.27%
217	STAT6(Stat)/CD4-Stat6-ChIP-Seq(GSE22104)/Homer	ABTTCYYRRGAA	1e-380	15.33%	9.29%
218	PRDM9(Zf)/Testis-DMC1-ChIP-Seq(GSE35498)/Homer	ADGGYAGYAGCATCT	1e-378	8.43%	4.07%
219	EWS:FLI1-fusion(ETS)/SK_N_MC-EWS:FLI1-ChIP-Seq(SRA014231)/Homer	VACAGGAAAT	1e-373	12.59%	7.18%
220	At3g45610(C2C2dof)/col-At3g45610-DAP-Seq(GSE60143)/Homer	TWACTTTTTS	1e-373	51.45%	42.14%
221	SOL1(CPP)/colamp-SOL1-DAP-Seq(GSE60143)/Homer	ATTTAAATHN	1e-373	48.73%	39.48%
222	OCT:OCT(POU,Homeobox)/NPC-OCT6-ChIP-Seq(GSE43916)/Homer	YATGCATATRCATRT	1e-372	4.68%	1.65%
223	STAT1(Stat)/HelaS3-STAT1-ChIP-Seq(GSE12782)/Homer	NATTTCCNGGAAAT	1e-372	9.38%	4.79%
224	At5g04390(C2H2)/col200-At5g04390-DAP-Seq(GSE60143)/Homer	AGTGANDN	1e-370	89.14%	82.54%
225	ASHR1(ND)/col-ASHR1-DAP-Seq(GSE60143)/Homer	NTGGTGAN	1e-368	27.49%	19.71%
226	SeqBias: TA-repeat	TATATATATA	1e-368	79.52%	71.45%
227	Nkx3.1(Homeobox)/LNCaP-Nkx3.1-ChIP-Seq(GSE28264)/Homer	AAGCACTTAA	1e-367	53.44%	44.18%
228	EBF(EBF)/proBcell-EBF-ChIP-Seq(GSE21978)/Homer	DGTCCCYRGGGA	1e-363	3.84%	1.19%
229	WIP5(C2H2)/colamp-WIP5-DAP-Seq(GSE60143)/Homer	TDTTCTCMAGGT	1e-360	27.33%	19.65%
230	AT2G28810(C2C2dof)/colamp-AT2G28810-DAP-Seq(GSE60143)/Homer	VAAAAAGTWA	1e-355	63.78%	54.79%
231	Bapx1(Homeobox)/VertebralCol-Bapx1-ChIP-Seq(GSE36672)/Homer	TTRAGTGSYK	1e-352	49.29%	40.28%
232	IRF8(IRF)/BMDM-IRF8-ChIP-Seq(GSE77884)/Homer	GRAASTGAAAST	1e-341	8.92%	4.61%
233	PRDM10(Zf)/HEK293-PRDM10.eGFP-ChIP-Seq(Encode)/Homer	TGGTACATTCCA	1e-341	12.37%	7.22%
234	Sp1(Zf)/Promoter/Homer	GGCCCCGCCCCC	1e-338	2.88%	0.74%
235	NFkB-p65(RHD)/GM12787-p65-ChIP-Seq(GSE19485)/Homer	WGGGGATTTCCC	1e-338	10.40%	5.73%
236	GT2(Trihelix)/colamp-GT2-DAP-Seq(GSE60143)/Homer	AMGGTAAAWWWN	1e-337	47.05%	38.28%
237	NF-E2(bZIP)/K562-NFE2-ChIP-Seq(GSE31477)/Homer	GATGACTCAGCA	1e-336	2.50%	0.56%
238	ERb(NR),IR3/Ovary-ERb-ChIP-Seq(GSE203391)/Homer	RGGTCAGGGTGACCT	1e-335	5.69%	2.40%
239	WRKY8(WRKY)/colamp-WRKY8-DAP-Seq(GSE60143)/Homer	CGTTGACTTT	1e-333	6.29%	2.80%
240	ZNF341(Zf)/EBV-ZNF341-ChIP-Seq(GSE113194)/Homer	GGAACAGCCG	1e-333	12.15%	7.10%
241	CDF3(C2C2dof)/colamp-CDF3-DAP-Seq(GSE60143)/Homer	AAAAGTRM	1e-327	53.50%	44.75%
242	AT5G63260(C3H)/col-AT5G63260-DAP-Seq(GSE60143)/Homer	RAAAAAGTRA	1e-324	66.51%	58.04%
243	Zfp809(Zf)/ES-Zfp809-ChIP-Seq(GSE70799)/Homer	GGGGCTYGKCTGGGA	1e-320	3.02%	0.86%
244	PIF4(bHLH)/Seedling-PIF4-ChIP-Seq(GSE35315)/Homer	NNBCACGTGN	1e-315	24.78%	17.84%
245	Egr1(Zf)/K562-Egr1-ChIP-Seq(GSE32465)/Homer	TGCGTGGGYG	1e-311	9.23%	5.00%
246	PU.1:IRF8(ETS:IRF)/pDC-Irf8-ChIP-Seq(GSE66899)/Homer	GGAAGTGAAAST	0,00E+00	5.16%	2.15%
247	SGR5(C2H2)/colamp-SGR5-DAP-Seq(GSE60143)/Homer	TTTGTCTTTTTT	0,00E+00	22.37%	15.78%
248	Bach1(bZIP)/K562-Bach1-ChIP-Seq(GSE31477)/Homer	AWWNTGCTGAGTCAT	0,00E+00	2.68%	0.70%
249	Sox2(HMG)/mES-Sox2-ChIP-Seq(GSE11431)/Homer	BCCATTGTTC	1,00E-303	25.88%	18.95%
250	dof24(C2C2dof)/col-dof24-DAP-Seq(GSE60143)/Homer	TWMCTTTTTG	1,00E-302	57.08%	48.68%
251	ERE(NR),IR3/MCF7-ERa-ChIP-Seq(Unpublished)/Homer	VAGGTCACNSTGACC	1,00E-301	5.47%	2.39%
252	GTL1(Trihelix)/colamp-GTL1-DAP-Seq(GSE60143)/Homer	WWTTTACCKY	1,00E-300	44.70%	36.50%
253	NPAS2(bHLH)/Liver-NPAS2-ChIP-Seq(GSE39860)/Homer	KCCACGTGAC	1,00E-297	21.92%	15.51%
254	TCX2(CPP)/colamp-TCX2-DAP-Seq(GSE60143)/Homer	NNWWTTYRAAHN	1,00E-296	49.18%	40.91%
255	COG1(C2C2dof)/col-COG1-DAP-Seq(GSE60143)/Homer	DAAAAAGTGA	1,00E-295	48.04%	39.81%
256	WRKY28(WRKY)/col-WRKY28-DAP-Seq(GSE60143)/Homer	BGTTGACTWH	1,00E-295	38.38%	30.52%
257	Tbx20(T-box)/Heart-Tbx20-ChIP-Seq(GSE29636)/Homer	GGTGYTGACAGS	1,00E-294	5.76%	2.60%
258	Zscan4c(Zf)/ESC-Zscan4c-ChIP-seq(GSE140619)/Homer	ATRTGTGCAC	1,00E-289	9.91%	5.65%
259	NFAT:AP1(RHD,bZIP)/Jurkat-NFATC1-ChIP-Seq(Jolma_et_al.)/Homer	SARTGGAAAAWRTGAGTCAB	1,00E-289	5.52%	2.47%
260	FXR(NR),ER2/Liver-FXR-ChIP-Seq(GSE133700)/Homer	NTGACCYMKRGGTCA	1,00E-283	9.07%	5.05%
261	FUS3(ABI3VP1)/col-FUS3-DAP-Seq(GSE60143)/Homer	DNNWTNTGCATGKNN	1,00E-282	23.73%	17.26%
262	Reverb(NR),DR2/RAW-Reverba.biotin-ChIP-Seq(GSE45914)/Homer	GTRGGTCASTGGGTCA	1,00E-281	2.90%	0.88%
263	Nrf2(bZIP)/Lymphoblast-Nrf2-ChIP-Seq(GSE37589)/Homer	HTGCTGAGTCAT	1,00E-279	2.08%	0.47%
264	Twist(bHLH)/HMLE-TWIST1-ChIP-Seq(Chang_et_al)/Homer	VCAKCTGGNNNCCAGMTGBN	1,00E-274	2.47%	0.67%
265	Nkx2.2(Homeobox)/NPC-Nkx2.2-ChIP-Seq(GSE61673)/Homer	BTBRAGTGSN	1,00E-273	40.88%	33.19%
266	GATA(Zf),IR4/iTreg-Gata3-ChIP-Seq(GSE20898)/Homer	NAGATWNBNATCTNN	1,00E-271	5.85%	2.77%
267	At1g14580(C2H2)/colamp-At1g14580-DAP-Seq(GSE60143)/Homer	CASAAAAMGACAAAA	1,00E-270	7.93%	4.27%
268	At4g38000(C2C2dof)/col-At4g38000-DAP-Seq(GSE60143)/Homer	WWWTWACTTTTT	1,00E-267	46.79%	38.98%
269	At1g64620(C2C2dof)/colamp-At1g64620-DAP-Seq(GSE60143)/Homer	CACTTTTT	1,00E-263	46.15%	38.42%
270	BMAL1(bHLH)/Liver-Bmal1-ChIP-Seq(GSE39860)/Homer	GNCACGTG	1,00E-262	35.72%	28.45%
271	At2g41835(C2H2)/col-At2g41835-DAP-Seq(GSE60143)/Homer	TTTGAAAA	1,00E-262	19.14%	13.45%
272	ARE(NR)/LNCAP-AR-ChIP-Seq(GSE27824)/Homer	RGRACASNSTGTYCYB	1,00E-261	5.03%	2.26%
273	GABPA(ETS)/Jurkat-GABPa-ChIP-Seq(GSE17954)/Homer	RACCGGAAGT	1,00E-260	18.52%	12.93%
274	NFE2L2(bZIP)/HepG2-NFE2L2-ChIP-Seq(Encode)/Homer	AWWWTGCTGAGTCAT	1,00E-254	2.67%	0.82%
275	AT2G31460(REMB3)/col-AT2G31460-DAP-Seq(GSE60143)/Homer	WNWARWDGAAATGAT	1,00E-253	11.46%	7.10%
276	MNT(bHLH)/HepG2-MNT-ChIP-Seq(Encode)/Homer	DGCACACGTG	1,00E-253	21.24%	15.38%
277	Adof1(C2C2dof)/col-Adof1-DAP-Seq(GSE60143)/Homer	NRWAAAGYDV	1,00E-251	71.84%	64.66%
278	OBP4(C2C2dof)/col-OBP4-DAP-Seq(GSE60143)/Homer	WTTHACTTTTTB	1,00E-251	50.59%	42.95%
279	AT3G12130(C3H)/colamp-AT3G12130-DAP-Seq(GSE60143)/Homer	TMACTTTTTV	1,00E-248	65.04%	57.59%
280	AT2G38300(G2like)/col-AT2G38300-DAP-Seq(GSE60143)/Homer	ADRGAATGTT	1,00E-246	46.94%	39.43%
281	OCT4-SOX2-TCF-NANOG(POU,Homeobox,HMG)/mES-Oct4-ChIP-Seq(GSE11431)/Homer	ATTTGCATAACAATG	1,00E-243	8.31%	4.72%
282	Smad4(MAD)/ESC-SMAD4-ChIP-Seq(GSE29422)/Homer	VBSYGTCTGG	1,00E-241	28.83%	22.35%
283	Nkx2.1(Homeobox)/LungAC-Nkx2.1-ChIP-Seq(GSE43252)/Homer	RSCACTYRAG	1,00E-240	56.89%	49.40%
284	WRKY27(WRKY)/colamp-WRKY27-DAP-Seq(GSE60143)/Homer	NHGTTGACYTWD	1,00E-240	29.65%	23.10%
285	OBP3(C2C2dof)/col-OBP3-DAP-Seq(GSE60143)/Homer	NYWACTTTTT	1,00E-238	75.01%	68.24%
286	CHR(?)/Hela-CellCycle-Expression/Homer	SRGTTTCAAA	1,00E-235	21.24%	15.57%
287	IRF1(IRF)/PBMC-IRF1-ChIP-Seq(GSE43036)/Homer	GAAAGTGAAAGT	1,00E-233	4.31%	1.89%
288	RBFox2(?)/Heart-RBFox2-CLIP-Seq(GSE57926)/Homer	TGCATGCA	1,00E-232	43.89%	36.69%
289	AT3G52440(C2C2dof)/colamp-AT3G52440-DAP-Seq(GSE60143)/Homer	DTHACTTTTT	1,00E-231	66.07%	58.92%
290	At5g62940(C2C2dof)/col-At5g62940-DAP-Seq(GSE60143)/Homer	WHWHHACTTTTT	1,00E-227	78.82%	72.50%
291	Pitx1:Ebox(Homeobox,bHLH)/Hindlimb-Pitx1-ChIP-Seq(GSE41591)/Homer	YTAATTRAWWCCAGATGT	1,00E-226	7.71%	4.36%
292	c-Jun-CRE(bZIP)/K562-cJun-ChIP-Seq(GSE31477)/Homer	ATGACGTCATCY	1,00E-225	7.47%	4.19%
293	Lola-I(Zf)/Embryo-LolaI-ChIP-Seq(GSE200870)/Homer	VAAAGCTY	1,00E-221	14.45%	9.84%
294	Oct6(POU,Homeobox)/NPC-Pou3f1-ChIP-Seq(GSE35496)/Homer	WATGCAAATGAG	1,00E-221	15.63%	10.85%
295	HSF3(HSF)/colamp-HSF3-DAP-Seq(GSE60143)/Homer	NTTCTAGAAKCTTCT	1,00E-220	25.60%	19.66%
296	Gli3(Zf)/E11.5-Gli3-ChIP-Chip(GSE151646)/Homer	RGACCACCCASR	1,00E-219	5.66%	2.88%
297	AT5G47660(Trihelix)/colamp-AT5G47660-DAP-Seq(GSE60143)/Homer	AWTTTTACCG	1,00E-219	52.52%	45.37%
298	AT2G20110(CPP)/colamp-AT2G20110-DAP-Seq(GSE60143)/Homer	ATTYAAATHY	1,00E-217	47.37%	40.32%
299	SpiB(ETS)/OCILY3-SPIB-ChIP-Seq(GSE56857)/Homer	AAAGRGGAAGTG	1,00E-217	5.85%	3.04%
300	bHLH80(bHLH)/col-bHLH80-DAP-Seq(GSE60143)/Homer	NNNNDCAASTTGHNN	1,00E-216	20.48%	15.12%
301	ZNF415(Zf)/HEK293-ZNF415.GFP-ChIP-Seq(GSE58341)/Homer	GRTGMTRGAGCC	1,00E-213	8.98%	5.43%
302	VIP1(bZIP)/col-VIP1-DAP-Seq(GSE60143)/Homer	NTTGACAGCTGTCAN	1,00E-212	4.42%	2.05%
303	Mef2c(MADS)/GM12878-Mef2c-ChIP-Seq(GSE32465)/Homer	DCYAAAAATAGM	1,00E-212	17.97%	12.96%
304	WRKY29(WRKY)/colamp-WRKY29-DAP-Seq(GSE60143)/Homer	MGTTGACTTT	1,00E-211	32.51%	26.17%
305	WRKY22(WRKY)/colamp-WRKY22-DAP-Seq(GSE60143)/Homer	WWAAAGTCAACK	1,00E-210	20.84%	15.50%
306	ZSCAN22(Zf)/HEK293-ZSCAN22.GFP-ChIP-Seq(GSE58341)/Homer	SMCAGTCWGAKGGAGGAGGC	1,00E-210	1.20%	0.19%
307	MafF(bZIP)/HepG2-MafF-ChIP-Seq(GSE31477)/Homer	HWWGTCAGCAWWTTT	1,00E-209	10.91%	7.02%
308	JGL(C2H2)/col-JGL-DAP-Seq(GSE60143)/Homer	ACYTTCAGTT	1,00E-207	44.60%	37.77%
309	Smad2(MAD)/ES-SMAD2-ChIP-Seq(GSE29422)/Homer	CTGTCTGG	1,00E-205	28.39%	22.42%
310	BBX31(Orphan)/col-BBX31-DAP-Seq(GSE60143)/Homer	NAAAAAGTDA	1,00E-203	52.10%	45.22%
311	ERF15(AP2EREBP)/colamp-ERF15-DAP-Seq(GSE60143)/Homer	WDHAGCMGCCAT	1,00E-202	27.80%	21.91%
312	AT5G66940(C2C2dof)/col-AT5G66940-DAP-Seq(GSE60143)/Homer	NNHACTTTWT	1,00E-202	60.85%	54.05%
313	NFkB2-p52(RHD)/U266-NFKB2-ChIP-seq(GSE230466)/Homer	VGGGRATTYCCC	1,00E-201	6.47%	3.59%
314	AT1G47655(C2C2dof)/colamp-AT1G47655-DAP-Seq(GSE60143)/Homer	YHACTTTTTS	1,00E-201	79.60%	73.76%
315	dof45(C2C2dof)/col-dof45-DAP-Seq(GSE60143)/Homer	NVAWAAAGTN	1,00E-198	70.47%	64.05%
316	RUNX(Runt)/HPC7-Runx1-ChIP-Seq(GSE22178)/Homer	SAAACCACAG	1,00E-196	16.84%	12.14%
317	p63(p53)/Keratinocyte-p63-ChIP-Seq(GSE17611)/Homer	NNDRCATGYCYNRRCATGYH	1,00E-196	7.58%	4.46%
318	FXR(NR),IR1/Liver-FXR-ChIP-Seq(Chong_et_al.)/Homer	AGGTCANTGACCTB	1,00E-187	7.38%	4.36%
319	LXRE(NR),DR4/RAW-LXRb.biotin-ChIP-Seq(GSE21512)/Homer	RGGTTACTANAGGTCA	1,00E-185	2.27%	0.78%
320	KLF17(Zf)/2cell-Klf17-CutnTag(GSE211845)/Homer	GCYCCGCCCCYH	1,00E-185	6.24%	3.50%
321	Nkx2.5(Homeobox)/HL1-Nkx2.5.biotin-ChIP-Seq(GSE21529)/Homer	RRSCACTYAA	1,00E-185	47.22%	40.71%
322	PIF5ox(bHLH)/Arabidopsis-PIF5ox-ChIP-Seq(GSE35062)/Homer	BCACGTGVDN	1,00E-185	20.05%	15.12%
323	OBP1(C2C2dof)/col-OBP1-DAP-Seq(GSE60143)/Homer	NHHACTTTWT	1,00E-183	74.01%	68.04%
324	At3g60580(C2H2)/col-At3g60580-DAP-Seq(GSE60143)/Homer	WTTYTACT	1,00E-183	89.78%	85.40%
325	Sox6(HMG)/Myotubes-Sox6-ChIP-Seq(GSE32627)/Homer	CCATTGTTNY	1,00E-183	44.07%	37.66%
326	ZNF519(Zf)/HEK293-ZNF519.GFP-ChIP-Seq(GSE58341)/Homer	GAGSCCGAGC	1,00E-176	2.66%	1.04%
327	AT1G76880(Trihelix)/col-AT1G76880-DAP-Seq(GSE60143)/Homer	ACGGTAAAAW	1,00E-174	18.35%	13.73%
328	ARF2(ARF)/col-ARF2-DAP-Seq(GSE60143)/Homer	TTGTCGGMWN	1,00E-173	51.66%	45.29%
329	PGR(NR)/EndoStromal-PGR-ChIP-Seq(GSE69539)/Homer	AAGAACATWHTGTTC	1,00E-173	7.45%	4.52%
330	TR4(NR),DR1/Hela-TR4-ChIP-Seq(GSE24685)/Homer	GAGGTCAAAGGTCA	1,00E-173	2.18%	0.76%
331	dof43(C2C2dof)/colamp-dof43-DAP-Seq(GSE60143)/Homer	NAAAAAGTDA	1,00E-172	48.89%	42.58%
332	GATA3(Zf),DR4/iTreg-Gata3-ChIP-Seq(GSE20898)/Homer	AGATGKDGAGATAAG	1,00E-171	5.06%	2.72%
333	At5g52660(MYBrelated)/colamp-At5g52660-DAP-Seq(GSE60143)/Homer	HAAAAATATCTW	1,00E-169	21.39%	16.52%
334	ETS:E-box(ETS,bHLH)/HPC7-Scl-ChIP-Seq(GSE22178)/Homer	AGGAARCAGCTG	1,00E-168	1.92%	0.63%
335	Initiator/Drosophila-Promoters/Homer	NTCAGTYG	1,00E-166	51.66%	45.44%
336	SEP3(MADS)/Arabidoposis-Flower-Sep3-ChIP-Seq/Homer	CCAAAAAGGG	1,00E-164	34.63%	28.90%
337	bZIP69(bZIP)/col-bZIP69-DAP-Seq(GSE60143)/Homer	GACAGCTGKCAW	1,00E-164	2.46%	0.96%
338	HSFA6B(HSF)/colamp-HSFA6B-DAP-Seq(GSE60143)/Homer	NTTCTAGAANHTTCT	1,00E-163	15.28%	11.17%
339	AT2G40260(G2like)/colamp-AT2G40260-DAP-Seq(GSE60143)/Homer	WAAAYATTCTTT	1,00E-162	52.40%	46.24%
340	Rfx1(HTH)/NPC-H3K4me1-ChIP-Seq(GSE16256)/Homer	KGTTGCCATGGCAA	1,00E-161	3.80%	1.86%
341	LEF1(HMG)/H1-LEF1-ChIP-Seq(GSE64758)/Homer	CCTTTGATST	1,00E-160	19.39%	14.84%
342	ZNF165(Zf)/WHIM12-ZNF165-ChIP-Seq(GSE65937)/Homer	AAGGKGRCGCAGGCA	1,00E-160	1.72%	0.54%
343	AT5G23930(mTERF)/col-AT5G23930-DAP-Seq(GSE60143)/Homer	GGCGGCTG	1,00E-159	18.32%	13.89%
344	WRKY55(WRKY)/col-WRKY55-DAP-Seq(GSE60143)/Homer	NCGTTGACTT	1,00E-159	32.37%	26.84%
345	GATA3(Zf),DR8/iTreg-Gata3-ChIP-Seq(GSE20898)/Homer	AGATSTNDNNDSAGATAASN	1,00E-154	3.67%	1.80%
346	Pbx3(Homeobox)/GM12878-PBX3-ChIP-Seq(GSE32465)/Homer	SCTGTCAMTCAN	1,00E-150	5.05%	2.83%
347	CEBP:AP1(bZIP)/ThioMac-CEBPb-ChIP-Seq(GSE21512)/Homer	DRTGTTGCAA	1,00E-150	23.83%	19.02%
348	Hoxa10(Homeobox)/ChickenMSG-Hoxa10.Flag-ChIP-Seq(GSE86088)/Homer	GGYAATGAAA	1,00E-148	19.76%	15.33%
349	WRKY42(WRKY)/colamp-WRKY42-DAP-Seq(GSE60143)/Homer	NAAAGTCAACGN	1,00E-148	12.36%	8.80%
350	BIM2(bHLH)/col-BIM2-DAP-Seq(GSE60143)/Homer	NNNNCACGTGNN	1,00E-148	19.11%	14.76%
351	Tbox:Smad(T-box,MAD)/ESCd5-Smad2_3-ChIP-Seq(GSE29422)/Homer	AGGTGHCAGACA	1,00E-147	4.05%	2.11%
352	AT2G28920(ND)/col-AT2G28920-DAP-Seq(GSE60143)/Homer	WAGATATTTWTW	1,00E-146	13.72%	10.00%
353	Nur77(NR)/K562-NR4A1-ChIP-Seq(GSE31363)/Homer	TGACCTTTNCNT	1,00E-145	6.30%	3.82%
354	WRKY71(WRKY)/col-WRKY71-DAP-Seq(GSE60143)/Homer	CKTTGACYWW	1,00E-145	23.33%	18.64%
355	Tbx21(T-box)/GM12878-TBX21-ChIP-Seq(Encode)/Homer	AGGTGTGAAA	1,00E-144	25.09%	20.27%
356	WRKY14(WRKY)/colamp-WRKY14-DAP-Seq(GSE60143)/Homer	NCGTTGACTTTN	1,00E-144	17.51%	13.37%
357	HRE(HSF)/Striatum-HSF1-ChIP-Seq(GSE38000)/Homer	TTCTAGAABNTTCTA	1,00E-144	4.97%	2.81%
358	HSFC1(HSF)/col-HSFC1-DAP-Seq(GSE60143)/Homer	HTTCTAGAADCTTCT	1,00E-143	5.65%	3.33%
359	ZNF768(Zf)/Rajj-ZNF768-ChIP-Seq(GSE111879)/Homer	RHHCAGAGAGGB	1,00E-142	0.89%	0.16%
360	Brn1(POU,Homeobox)/NPC-Brn1-ChIP-Seq(GSE35496)/Homer	TATGCWAATBAV	1,00E-139	11.97%	8.57%
361	WRKY6(WRKY)/colamp-WRKY6-DAP-Seq(GSE60143)/Homer	NCGTTGACTWWD	1,00E-137	25.89%	21.13%
362	WRKY31(WRKY)/colamp-WRKY31-DAP-Seq(GSE60143)/Homer	NCGTTGACTWWK	1,00E-136	21.60%	17.18%
363	Hoxd10(Homeobox)/ChickenMSG-Hoxd10.Flag-ChIP-Seq(GSE86088)/Homer	GGCMATGAAA	1,00E-136	36.16%	30.86%
364	WUS1(Homeobox)/colamp-WUS1-DAP-Seq(GSE60143)/Homer	CAWTCATTCA	1,00E-136	14.81%	11.08%
365	LCL1(MYBrelated)/colamp-LCL1-DAP-Seq(GSE60143)/Homer	NAAAATATCTWHWWN	1,00E-136	7.30%	4.69%
366	WRKY21(WRKY)/colamp-WRKY21-DAP-Seq(GSE60143)/Homer	DCGTTGACTTTT	1,00E-135	4.77%	2.71%
367	bHLH122(bHLH)/col100-bHLH122-DAP-Seq(GSE60143)/Homer	NDDCAASTTGHHNWW	1,00E-134	18.41%	14.32%
368	WRKY26(WRKY)/colamp-WRKY26-DAP-Seq(GSE60143)/Homer	CGTTGACTWDKN	1,00E-134	16.32%	12.45%
369	Sox17(HMG)/Endoderm-Sox17-ChIP-Seq(GSE61475)/Homer	CCATTGTTYB	1,00E-133	21.69%	17.32%
370	Six4(Homeobox)/MCF7-SIX4-ChIP-Seq(Encode)/Homer	TGWAAYCTGABACCB	1,00E-132	2.23%	0.93%
371	Hoxd12(Homeobox)/ChickenMSG-Hoxd12.Flag-ChIP-Seq(GSE86088)/Homer	HDGYAATGAAAN	1,00E-132	54.72%	49.18%
372	Foxh1(Forkhead)/hESC-FOXH1-ChIP-Seq(GSE29422)/Homer	NNTGTGGATTSS	1,00E-130	21.96%	17.61%
373	WRKY24(WRKY)/colamp-WRKY24-DAP-Seq(GSE60143)/Homer	CGTTGACTWW	1,00E-129	26.27%	21.62%
374	SpliceDonor/U1snRNP/Homer	MAGGTAAGTN	1,00E-128	83.75%	79.47%
375	CELF2(RRM)/JSL1-CELF2-CLIP-Seq(GSE71264)/Homer	RGTGTCAG	1,00E-128	6.93%	4.46%
376	AT3G09735(S1Falike)/col-AT3G09735-DAP-Seq(GSE60143)/Homer	TTCTAGAANMTTCTA	1,00E-127	11.72%	8.49%
377	WRKY65(WRKY)/colamp-WRKY65-DAP-Seq(GSE60143)/Homer	AWWWAGTCAACG	1,00E-126	17.17%	13.32%
378	WRKY45(WRKY)/col-WRKY45-DAP-Seq(GSE60143)/Homer	HNNNKTTGACTWWNH	1,00E-125	16.16%	12.42%
379	WRKY75(WRKY)/col-WRKY75-DAP-Seq(GSE60143)/Homer	CGTTGACTWW	1,00E-125	28.08%	23.39%
380	At3g09600(MYBrelated)/colamp-At3g09600-DAP-Seq(GSE60143)/Homer	AAAATATCTT	1,00E-124	21.01%	16.85%
381	WRKY40(WRKY)/colamp-WRKY40-DAP-Seq(GSE60143)/Homer	AHWAGTCAAC	1,00E-122	18.42%	14.50%
382	Zelda(Zf)/Embryo-zld-ChIP-Seq(GSE65441)/Homer	KBCTACCTGW	1,00E-122	11.63%	8.48%
383	Atf2(bZIP)/3T3L1-Atf2-ChIP-Seq(GSE56872)/Homer	NRRTGACGTCAT	1,00E-121	7.48%	4.97%
384	Tcf4(HMG)/Hct116-Tcf4-ChIP-Seq(SRA012054)/Homer	ASATCAAAGGVA	1,00E-121	12.35%	9.11%
385	Rfx5(HTH)/GM12878-Rfx5-ChIP-Seq(GSE31477)/Homer	SCCTAGCAACAG	1,00E-120	6.89%	4.50%
386	Oct4:Sox17(POU,Homeobox,HMG)/F9-Sox17-ChIP-Seq(GSE44553)/Homer	CCATTGTATGCAAAT	1,00E-120	5.87%	3.68%
387	WRKY15(WRKY)/col-WRKY15-DAP-Seq(GSE60143)/Homer	VGTTGACTWW	1,00E-119	29.25%	24.61%
388	SCRT1(Zf)/HEK293-SCRT1.eGFP-ChIP-Seq(Encode)/Homer	GCAACAGGTG	1,00E-118	7.71%	5.19%
389	WRKY43(WRKY)/colamp-WRKY43-DAP-Seq(GSE60143)/Homer	NCGTTGACTTTT	1,00E-117	12.79%	9.54%
390	HRE(HSF)/HepG2-HSF1-ChIP-Seq(GSE31477)/Homer	BSTTCTRGAABVTTCYAGAA	1,00E-116	2.76%	1.36%
391	Eomes(T-box)/H9-Eomes-ChIP-Seq(GSE26097)/Homer	ATTAACACCT	1,00E-115	50.69%	45.51%
392	WRKY50(WRKY)/col-WRKY50-DAP-Seq(GSE60143)/Homer	NNTTGACTWNNGNNN	1,00E-115	29.35%	24.78%
393	X-box(HTH)/NPC-H3K4me1-ChIP-Seq(GSE16256)/Homer	GGTTGCCATGGCAA	1,00E-113	2.35%	1.08%
394	c-Myc(bHLH)/mES-cMyc-ChIP-Seq(GSE11431)/Homer	VVCCACGTGG	1,00E-113	8.12%	5.58%
395	ANAC005(NAC)/col-ANAC005-DAP-Seq(GSE60143)/Homer	WVCTTVTWNHABAAG	1,00E-112	12.71%	9.55%
396	ISRE(IRF)/ThioMac-LPS-Expression(GSE23622)/Homer	AGTTTCASTTTC	1,00E-112	1.91%	0.80%
397	Gata6(Zf)/HUG1N-GATA6-ChIP-Seq(GSE51936)/Homer	YCTTATCTBN	1,00E-112	35.86%	31.07%
398	Pit1(Homeobox)/GCrat-Pit1-ChIP-Seq(GSE58009)/Homer	ATGMATATDC	1,00E-110	37.65%	32.82%
399	Gata2(Zf)/K562-GATA2-ChIP-Seq(GSE18829)/Homer	BBCTTATCTS	1,00E-110	25.91%	21.63%
400	ETV4(ETS)/HepG2-ETV4-ChIP-Seq(ENCODE)/Homer	ACCGGAAGTG	1,00E-109	20.62%	16.72%
401	bHLH130(bHLH)/col-bHLH130-DAP-Seq(GSE60143)/Homer	GCAACTTG	1,00E-109	15.62%	12.17%
402	Six1(Homeobox)/Myoblast-Six1-ChIP-Chip(GSE20150)/Homer	GKVTCADRTTWC	1,00E-109	8.63%	6.05%
403	ZNF692(Zf)/HEK293-ZNF692.GFP-ChIP-Seq(GSE58341)/Homer	GTGGGCCCCA	1,00E-109	2.00%	0.87%
404	Dorsal(RHD)/Embryo-dl-ChIP-Seq(GSE65441)/Homer	GGGAAAAMCCCG	1,00E-109	4.55%	2.72%
405	VDR(NR),DR3/GM10855-VDR+vitD-ChIP-Seq(GSE22484)/Homer	ARAGGTCANWGAGTTCANNN	1,00E-108	4.10%	2.38%
406	PAX5(Paired,Homeobox)/GM12878-PAX5-ChIP-Seq(GSE32465)/Homer	GCAGCCAAGCRTGACH	1,00E-108	6.79%	4.53%
407	Max(bHLH)/K562-Max-ChIP-Seq(GSE31477)/Homer	RCCACGTGGYYN	1,00E-106	12.14%	9.12%
408	SFP1/SacCer-Promoters/Homer	DDAAAAATTTTY	1,00E-106	4.55%	2.74%
409	NTM1(NAC)/col-NTM1-DAP-Seq(GSE60143)/Homer	ACTTRTARAASAAGT	1,00E-106	11.88%	8.89%
410	Gli2(Zf)/GM2-Gli2-ChIP-Chip(GSE112702)/Homer	YSTGGGTGGTCT	1,00E-106	3.50%	1.94%
411	RAR:RXR(NR),DR0/ES-RAR-ChIP-Seq(GSE56893)/Homer	AGGTCAAGGTCA	1,00E-105	2.35%	1.12%
412	CEBP:CEBP(bZIP)/MEF-Chop-ChIP-Seq(GSE35681)/Homer	NTNATGCAAYMNNHTGMAAY	1,00E-101	5.08%	3.21%
413	STOP1(C2H2)/colamp-STOP1-DAP-Seq(GSE60143)/Homer	DTATCTGGKGRAGGT	1,00E-101	8.92%	6.40%
414	Unknown4/Arabidopsis-Promoters/Homer	CKTCKTCTTY	1,00E-100	19.36%	15.72%
415	PBX1(Homeobox)/MCF7-PBX1-ChIP-Seq(GSE28007)/Homer	GSCTGTCACTCA	1,00E-100	1.55%	0.62%

Supplementary Table 4

Motif analysis of MCF-7 specific FOXA1 sites (28,805) compared to background regions (123,121).

Rank	Motif Name	Consensus	P-value	FOXA1 regions (%)	Background regions (%)	Rank	Motif Name	Consensus	P-value	FOXA1 regions (%)	Background regions (%)
1	Foxa2(Forkhead)/Liver-Foxa2-ChIP-Seq(GSE25694)/Homer	CYTGTTTACWYW	1e-6483	67.21%	15.10%	113	p53(p53)/mES-cMyc-ChIP-Seq(GSE11431)/Homer	ACATGCCCGGGCAT	1,00E-233	1.45%	0.05%
2	FOXA1(Forkhead)/LNCAP-FOXA1-ChIP-Seq(GSE27824)/Homer	WAAGTAAACA	1e-6171	78.14%	24.78%	114	Smad3(MAD)/NPC-Smad3-ChIP-Seq(GSE36673)/Homer	TWGTCTGV	1,00E-229	37.51%	27.68%
3	FOXA1(Forkhead)/MCF7-FOXA1-ChIP-Seq(GSE26831)/Homer	WAAGTAAACA	1e-5999	72.84%	20.87%	115	Ascl2(bHLH)/ESC-Ascl2-ChIP-Seq(GSE97712)/Homer	SSRGCAGCTGCH	1,00E-227	9.91%	4.68%
4	Foxa3(Forkhead)/Liver-Foxa3-ChIP-Seq(GSE77670)/Homer	BSNTGTTTACWYWGN	1e-5762	48.76%	6.58%	116	Klf9(Zf)/GBM-Klf9-ChIP-Seq(GSE62211)/Homer	GCCACRCCCACY	1,00E-225	3.25%	0.68%
5	FOXM1(Forkhead)/MCF7-FOXM1-ChIP-Seq(GSE72977)/Homer	TRTTTACTTW	1e-5505	69.90%	20.32%	117	TEAD3(TEA)/HepG2-TEAD3-ChIP-Seq(Encode)/Homer	TRCATTCCAG	1,00E-223	23.56%	15.44%
6	Fox:Ebox(Forkhead,bHLH)/Panc1-Foxa2-ChIP-Seq(GSE47459)/Homer	NNNVCTGWGYAAACASN	1e-4852	55.42%	12.49%	118	ZEB1(Zf)/PDAC-ZEB1-ChIP-Seq(GSE64557)/Homer	VCAGGTRDRY	1,00E-223	20.61%	12.98%
7	FOXK2(Forkhead)/U2OS-FOXK2-ChIP-Seq(E-MTAB-2204)/Homer	SCHTGTTTACAT	1e-4257	50.21%	11.25%	119	Trl(Zf)/S2-GAGAfactor-ChIP-Seq(GSE40646)/Homer	RGAGAGAG	1,00E-217	28.26%	19.60%
8	FoxL2(Forkhead)/Ovary-FoxL2-ChIP-Seq(GSE60858)/Homer	WWTRTAAACAVG	1e-3988	62.48%	20.58%	120	At1g69690(TCP)/colamp-At1g69690-DAP-Seq(GSE60143)/Homer	NHGTGGGGCCCACHW	1,00E-216	3.71%	0.93%
9	FOXK1(Forkhead)/HEK293-FOXK1-ChIP-Seq(GSE51673)/Homer	NVWTGTTTAC	1e-3578	63.71%	23.44%	121	MyoD(bHLH)/Myotube-MyoD-ChIP-Seq(GSE21614)/Homer	RRCAGCTGYTSY	1,00E-214	6.34%	2.42%
10	Foxf1(Forkhead)/Lung-Foxf1-ChIP-Seq(GSE77951)/Homer	WWATRTAAACAN	1e-3176	60.81%	23.06%	122	THRb(NR)/HepG2-THRb.Flag-ChIP-Seq(Encode)/Homer	GGTCACCTGAGGTCA	1,00E-213	6.08%	2.27%
11	PHA-4(Forkhead)/cElegans-Embryos-PHA4-ChIP-Seq(modEncode)/Homer	KTGTTTGC	1e-3035	87.88%	52.23%	123	ZFX(Zf)/mES-Zfx-ChIP-Seq(GSE11431)/Homer	AGGCCTRG	1,00E-210	11.19%	5.76%
12	Foxo3(Forkhead)/U2OS-Foxo3-ChIP-Seq(E-MTAB-2701)/Homer	DGTAAACA	1e-2996	51.33%	16.76%	124	Esrrb(NR)/mES-Esrrb-ChIP-Seq(GSE11431)/Homer	KTGACCTTGA	1,00E-210	10.48%	5.25%
13	Foxo1(Forkhead)/RAW-Foxo1-ChIP-Seq(Fan_et_al.)/Homer	CTGTTTAC	1e-2752	59.55%	24.29%	125	EBF2(EBF)/BrownAdipose-EBF2-ChIP-Seq(GSE97114)/Homer	NABTCCCWDGGGAVH	1,00E-210	8.29%	3.72%
14	FOXP1(Forkhead)/H9-FOXP1-ChIP-Seq(GSE31006)/Homer	NYYTGTTTACHN	1e-2690	38.11%	9.55%	126	WRKY28(WRKY)/col-WRKY28-DAP-Seq(GSE60143)/Homer	BGTTGACTWH	1,00E-209	30.00%	21.29%
15	FoxD3(forkhead)/ZebrafishEmbryo-Foxd3.biotin-ChIP-seq(GSE106676)/Homer	TGTTTAYTTAGC	1e-2296	45.43%	15.95%	127	Sox9(HMG)/Limb-SOX9-ChIP-Seq(GSE73225)/Homer	AGGVNCCTTTGT	1,00E-204	15.55%	9.16%
16	SCL(bHLH)/HPC7-Scl-ChIP-Seq(GSE13511)/Homer	AVCAGCTG	1e-893	51.66%	31.27%	128	Bcl11a(Zf)/HSPC-BCL11A-ChIP-Seq(GSE104676)/Homer	TYTGACCASWRG	1,00E-203	9.02%	4.28%
17	SeqBias: GA-repeat	GAGAGAGAGA	1e-856	78.18%	58.93%	129	Atoh1(bHLH)/Cerebellum-Atoh1-ChIP-Seq(GSE22111)/Homer	VNRVCAGCTGGY	1,00E-203	11.39%	5.99%
18	ZNF416(Zf)/HEK293-ZNF416.GFP-ChIP-Seq(GSE58341)/Homer	WDNCTGGGCA	1e-713	15.39%	4.92%	130	Sox21(HMG)/ESC-SOX21-ChIP-Seq(GSE110505)/Homer	BCCWTTGTBYKV	1,00E-201	34.50%	25.50%
19	KLF1(Zf)/HUDEP2-KLF1-CutnRun(GSE136251)/Homer	VDGGGYGGGGCY	1e-687	10.52%	2.42%	131	MYRF(MYRF)/CFPAC1-MYRF-ChIP-Seq(GSE145627)/Homer	AGTGCCTGGCAC	1,00E-200	4.34%	1.33%
20	Fosl2(bZIP)/3T3L1-Fosl2-ChIP-Seq(GSE56872)/Homer	NATGASTCABNN	1e-672	10.98%	2.71%	132	ERRg(NR)/Kidney-ESRRG-ChIP-Seq(GSE104905)/Homer	GTGACCTTGRVN	1,00E-200	11.77%	6.30%
21	GAGA-repeat/SacCer-Promoters/Homer	CTYTCTYTCTCTCTC	1e-640	63.81%	46.15%	133	MyoG(bHLH)/C2C12-MyoG-ChIP-Seq(GSE36024)/Homer	AACAGCTG	1,00E-199	9.17%	4.42%
22	KLF5(Zf)/LoVo-KLF5-ChIP-Seq(GSE49402)/Homer	DGGGYGKGGC	1e-638	14.43%	4.76%	134	Atoh7(bHLH)/Retina-Atoh7-CutnRun(GSE156756)/Homer	KRRCAGCTGGTS	1,00E-199	7.50%	3.28%
23	Jun-AP1(bZIP)/K562-cJun-ChIP-Seq(GSE31477)/Homer	GATGASTCATCN	1e-634	8.38%	1.62%	135	NeuroD1(bHLH)/Islet-NeuroD1-ChIP-Seq(GSE30298)/Homer	GCCATCTGTT	1,00E-199	9.04%	4.34%
24	ZNF148(Zf)/MDAMB231-ZNF148-ChIP-Seq(GSE147020)/Homer	CCCCTCCCCCAC	1e-631	6.36%	0.82%	136	EHF(ETS)/LoVo-EHF-ChIP-Seq(GSE49402)/Homer	AVCAGGAAGT	1,00E-199	18.66%	11.74%
25	Fra2(bZIP)/Striatum-Fra2-ChIP-Seq(GSE43429)/Homer	GGATGACTCATC	1e-623	15.46%	5.46%	137	Meis1(Homeobox)/MastCells-Meis1-ChIP-Seq(GSE48085)/Homer	VGCTGWCAVB	1,00E-199	22.71%	15.14%
26	KLF14(Zf)/HEK293-KLF14.GFP-ChIP-Seq(GSE58341)/Homer	RGKGGGCGKGGC	1e-615	15.32%	5.43%	138	Oct4(POU,Homeobox)/mES-Oct4-ChIP-Seq(GSE11431)/Homer	ATTTGCATAW	1,00E-196	15.67%	9.37%
27	KLF3(Zf)/MEF-Klf3-ChIP-Seq(GSE44748)/Homer	NRGCCCCRCCCHBNN	1e-591	7.26%	1.26%	139	STAT4(Stat)/CD4-Stat4-ChIP-Seq(GSE22104)/Homer	NYTTCCWGGAAR	1,00E-192	16.55%	10.12%
28	JunB(bZIP)/DendriticCells-Junb-ChIP-Seq(GSE36099)/Homer	RATGASTCAT	1e-576	17.51%	7.07%	140	GRF9(GRF)/colamp-GRF9-DAP-Seq(GSE60143)/Homer	NWCTGACANNNNNNN	1,00E-191	16.05%	9.74%
29	CTCF(Zf)/CD4+-CTCF-ChIP-Seq(Barski_et_al.)/Homer	AYAGTGCCMYCTRGTGGCCA	1e-573	4.10%	0.23%	141	RUNX-AML(Runt)/CD4+-PolII-ChIP-Seq(Barski_et_al.)/Homer	GCTGTGGTTW	1,00E-191	11.84%	6.46%
30	Sp5(Zf)/mES-Sp5.Flag-ChIP-Seq(GSE72989)/Homer	RGKGGGCGGAGC	1e-565	10.03%	2.66%	142	TCP7(TCP)/col-TCP7-DAP-Seq(GSE60143)/Homer	GTGGGSCCCACHHNN	1,00E-182	5.72%	2.26%
31	Zfp281(Zf)/ES-Zfp281-ChIP-Seq(GSE81042)/Homer	CCCCTCCCCCAC	1e-556	3.57%	0.13%	143	ZNF7(Zf)/HepG2-ZNF7.Flag-ChIP-Seq(Encode)/Homer	CTGCCWVCTTTTRTA	1,00E-179	12.67%	7.24%
32	GLIS3(Zf)/Thyroid-Glis3.GFP-ChIP-Seq(GSE103297)/Homer	CTCCCTGGGAGGCCN	1e-554	13.33%	4.57%	144	Snail1(Zf)/LS174T-SNAIL1.HA-ChIP-Seq(GSE127183)/Homer	TRCACCTGCY	1,00E-179	9.17%	4.62%
33	AP-1(bZIP)/ThioMac-PU.1-ChIP-Seq(GSE21512)/Homer	VTGACTCATC	1e-551	20.49%	9.30%	145	MRE(NR)/Neuro2A-NR3C2-ChIPnexus(GSE115417)/Homer	GGAACAGAVTGTCCT	1,00E-179	16.55%	10.33%
34	Atf3(bZIP)/GBM-ATF3-ChIP-Seq(GSE33912)/Homer	DATGASTCATHN	1e-530	19.89%	9.05%	146	Tbox:Smad(T-box,MAD)/ESCd5-Smad2_3-ChIP-Seq(GSE29422)/Homer	AGGTGHCAGACA	1,00E-176	3.64%	1.07%
35	BATF(bZIP)/Th17-BATF-ChIP-Seq(GSE39756)/Homer	DATGASTCAT	1e-522	19.92%	9.14%	147	BPC1(BBRBPC)/colamp-BPC1-DAP-Seq(GSE60143)/Homer	GARGAGAGAGAA	1,00E-176	9.73%	5.07%
36	SpliceAcceptor/Homer	TYYYYRCAGG	1e-511	65.09%	49.37%	148	ERE(NR),IR3/MCF7-ERa-ChIP-Seq(Unpublished)/Homer	VAGGTCACNSTGACC	1,00E-174	4.04%	1.31%
37	Erra(NR)/HepG2-Erra-ChIP-Seq(GSE31477)/Homer	CAAAGGTCAG	1e-500	25.98%	13.86%	149	THRa(NR)/C17.2-THRa-ChIP-Seq(GSE38347)/Homer	GGTCANYTGAGGWCA	1,00E-169	5.33%	2.10%
38	Fra1(bZIP)/BT549-Fra1-ChIP-Seq(GSE46166)/Homer	NNATGASTCATH	1e-497	18.33%	8.23%	150	Sox15(HMG)/CPA-Sox15-ChIP-Seq(GSE62909)/Homer	RAACAATGGN	1,00E-167	24.12%	16.93%
39	NFATC2(RHD)/Islets-NFATC2-ChIP-Seq(GSE158496)/Homer	WTTTTCCATTGS	1e-497	37.73%	23.57%	151	WRKY27(WRKY)/colamp-WRKY27-DAP-Seq(GSE60143)/Homer	NHGTTGACYTWD	1,00E-167	23.28%	16.21%
40	SeqBias: CA-repeat	CACACACACA	1e-496	70.80%	55.66%	152	WRKY29(WRKY)/colamp-WRKY29-DAP-Seq(GSE60143)/Homer	MGTTGACTTT	1,00E-166	25.20%	17.89%
41	hTCT(CPE)	BCKTTCTYTTY	1e-491	38.43%	24.27%	153	RBPJ:Ebox(?,bHLH)/Panc1-Rbpj1-ChIP-Seq(GSE47459)/Homer	GGGRAARRGRMCAGMTG	1,00E-166	3.49%	1.04%
42	Ptf1a(bHLH)/Panc1-Ptf1a-ChIP-Seq(GSE47459)/Homer	ACAGCTGTTN	1e-490	28.10%	15.64%	154	Mesp1(bHLH)/ESC-Mesp1-ChIP-Seq(GSE165102)/Homer	RCCATTTGBY	1,00E-164	10.32%	5.64%
43	BORIS(Zf)/K562-CTCFL-ChIP-Seq(GSE32465)/Homer	CNNBRGCGCCCCCTGSTGGC	1e-484	3.51%	0.21%	155	EWS:ERG-fusion(ETS)/CADO_ES1-EWS:ERG-ChIP-Seq(SRA014231)/Homer	ATTTCCTGTN	1,00E-164	11.33%	6.41%
44	Fos(bZIP)/TSC-Fos-ChIP-Seq(GSE110950)/Homer	NDATGASTCAYN	1e-483	19.03%	8.83%	156	HNF4a(NR),DR1/HepG2-HNF4a-ChIP-Seq(GSE25021)/Homer	CARRGKBCAAAGTYCA	1,00E-164	6.62%	2.99%
45	Sox4(HMG)/proB-Sox4-ChIP-Seq(GSE50066)/Homer	YCTTTGTTCC	1e-462	20.63%	10.18%	157	STAT5(Stat)/mCD4+-Stat5-ChIP-Seq(GSE12346)/Homer	RTTTCTNAGAAA	1,00E-161	6.32%	2.81%
46	ZNF467(Zf)/HEK293-ZNF467.GFP-ChIP-Seq(GSE58341)/Homer	TGGGGAAGGGCM	1e-456	8.60%	2.39%	158	Tlx?(NR)/NPC-H3K4me1-ChIP-Seq(GSE16256)/Homer	CTGGCAGSCTGCCA	1,00E-158	3.52%	1.10%
47	KLF6(Zf)/PDAC-KLF6-ChIP-Seq(GSE64557)/Homer	MKGGGYGTGGCC	1e-454	10.89%	3.69%	159	Oct2(POU,Homeobox)/Bcell-Oct2-ChIP-Seq(GSE21512)/Homer	ATATGCAAAT	1,00E-157	10.02%	5.50%
48	Maz(Zf)/HepG2-Maz-ChIP-Seq(GSE31477)/Homer	GGGGGGGG	1e-453	9.56%	2.93%	160	FRS9(ND)/col-FRS9-DAP-Seq(GSE60143)/Homer	RGAGAGAGAAAG	1,00E-157	3.07%	0.86%
49	COUP-TFII(NR)/Artia-Nr2f2-ChIP-Seq(GSE46497)/Homer	AGRGGTCA	1e-445	26.09%	14.53%	161	Etv2(ETS)/ES-ER71-ChIP-Seq(GSE59402)/Homer	NNAYTTCCTGHN	1,00E-154	10.97%	6.26%
50	Sp2(Zf)/HEK293-Sp2.eGFP-ChIP-Seq(Encode)/Homer	YGGCCCCGCCCC	1e-439	13.71%	5.54%	162	NFAT(RHD)/Jurkat-NFATC1-ChIP-Seq(Jolma_et_al.)/Homer	ATTTTCCATT	1,00E-154	18.10%	12.02%
51	Bcl6(Zf)/Liver-Bcl6-ChIP-Seq(GSE31578)/Homer	NNNCTTTCCAGGAAA	1e-439	20.37%	10.20%	163	Rfx6(HTH)/Min6b1-Rfx6.HA-ChIP-Seq(GSE62844)/Homer	TGTTKCCTAGCAACM	1,00E-153	12.27%	7.28%
52	VRN1(ABI3VP1)/col-VRN1-DAP-Seq(GSE60143)/Homer	TTTTTTTTTT	1e-435	6.94%	1.64%	164	WRKY8(WRKY)/colamp-WRKY8-DAP-Seq(GSE60143)/Homer	CGTTGACTTT	1,00E-152	4.98%	2.01%
53	Klf15(Zf)/Liver-Klf15-ChIP-Seq(GSE166083)/Homer	RGGGGGCGGGGC	1e-433	10.98%	3.87%	165	MafA(bZIP)/Islet-MafA-ChIP-Seq(GSE30298)/Homer	TGCTGACTCA	1,00E-151	10.13%	5.66%
54	COUP-TFII(NR)/K562-NR2F1-ChIP-Seq(Encode)/Homer	GKBCARAGGTCA	1e-428	22.25%	11.73%	166	bZIP52(bZIP)/colamp-bZIP52-DAP-Seq(GSE60143)/Homer	NDNHCAGCTGTCANN	1,00E-149	20.99%	14.57%
55	EAR2(NR)/K562-NR2F6-ChIP-Seq(Encode)/Homer	NRBCARRGGTCA	1e-423	19.67%	9.84%	167	STZ(C2H2)/colamp-STZ-DAP-Seq(GSE60143)/Homer	HNBTCACT	1,00E-149	79.24%	71.82%
56	Sox10(HMG)/SciaticNerve-Sox3-ChIP-Seq(GSE35132)/Homer	CCWTTGTYYB	1e-420	34.54%	21.85%	168	AZF1(C2H2)/colamp-AZF1-DAP-Seq(GSE60143)/Homer	DKSWCACT	1,00E-148	69.64%	61.51%
57	RLR1?/SacCer-Promoters/Homer	WTTTTCYYTTTT	1e-420	19.67%	9.87%	169	Zic(Zf)/Cerebellum-ZIC1.2-ChIP-Seq(GSE60731)/Homer	CCTGCTGAGH	1,00E-147	8.43%	4.43%
58	EBF1(EBF)/Near-E2A-ChIP-Seq(GSE21512)/Homer	GTCCCCWGGGGA	1e-418	8.89%	2.73%	170	Egr1(Zf)/K562-Egr1-ChIP-Seq(GSE32465)/Homer	TGCGTGGGYG	1,00E-146	5.55%	2.43%
59	HEB(bHLH)/mES-Heb-ChIP-Seq(GSE53233)/Homer	VCAGCTGBNN	1e-406	18.70%	9.30%	171	NLP7(RWPRK)/col-NLP7-DAP-Seq(GSE60143)/Homer	TGRCCYTTCR	1,00E-146	26.71%	19.67%
60	HuR(?)/HEK293-HuR-CLIP-Seq(GSE87887)/Homer	BTTTGGTTTG	1e-404	77.64%	64.76%	172	GATA(Zf),IR3/iTreg-Gata3-ChIP-Seq(GSE20898)/Homer	NNNNNBAGATAWYATCTVHN	1,00E-144	6.45%	3.05%
61	GAGA-repeat/Arabidopsis-Promoters/Homer	CTCTCTCTCY	1e-398	13.92%	5.99%	173	Egr2(Zf)/Thymocytes-Egr2-ChIP-Seq(GSE34254)/Homer	NGCGTGGGCGGR	1,00E-144	2.14%	0.46%
62	Ascl1(bHLH)/NeuralTubes-Ascl1-ChIP-Seq(GSE55840)/Homer	NNVVCAGCTGBN	1e-393	14.71%	6.56%	174	RUNX2(Runt)/PCa-RUNX2-ChIP-Seq(GSE33889)/Homer	NWAACCACADNN	1,00E-143	13.81%	8.65%
63	AT5G60130(ABI3VP1)/col-AT5G60130-DAP-Seq(GSE60143)/Homer	WTTYTAAGVAAA	1e-380	51.25%	37.74%	175	Myf5(bHLH)/GM-Myf5-ChIP-Seq(GSE24852)/Homer	BAACAGCTGT	1,00E-143	6.29%	2.95%
64	E2A(bHLH)/proBcell-E2A-ChIP-Seq(GSE21978)/Homer	DNRCAGCTGY	1e-378	13.81%	6.07%	176	ZBTB18(Zf)/HEK293-ZBTB18.GFP-ChIP-Seq(GSE58341)/Homer	AACATCTGGA	1,00E-143	5.98%	2.74%
65	Tgif2(Homeobox)/mES-Tgif2-ChIP-Seq(GSE55404)/Homer	TGTCANYT	1e-376	54.00%	40.48%	177	Unknown2/Drosophila-Promoters/Homer	CATCMCTA	1,00E-143	15.25%	9.85%
66	Tgif1(Homeobox)/mES-Tgif1-ChIP-Seq(GSE55404)/Homer	YTGWCADY	1e-371	52.62%	39.23%	178	Tbx5(T-box)/HL1-Tbx5.biotin-ChIP-Seq(GSE21529)/Homer	AGGTGTCA	1,00E-142	40.44%	32.46%
67	TEAD(TEA)/Fibroblast-PU.1-ChIP-Seq(Unpublished)/Homer	YCWGGAATGY	1e-354	15.91%	7.76%	179	Stat3+il21(Stat)/CD4-Stat3-ChIP-Seq(GSE19198)/Homer	SVYTTCCNGGAARB	1,00E-141	9.42%	5.24%
68	MafB(bZIP)/BMM-Mafb-ChIP-Seq(GSE75722)/Homer	WNTGCTGASTCAGCANWTTY	1e-353	9.13%	3.24%	180	WRKY22(WRKY)/colamp-WRKY22-DAP-Seq(GSE60143)/Homer	WWAAAGTCAACK	1,00E-141	16.06%	10.55%
69	PU.1-IRF(ETS:IRF)/Bcell-PU.1-ChIP-Seq(GSE21512)/Homer	MGGAAGTGAAAC	1e-352	19.71%	10.60%	181	Zac1(Zf)/Neuro2A-Plagl1-ChIP-Seq(GSE75942)/Homer	HAWGRGGCCM	1,00E-141	22.92%	16.43%
70	Unknown-ESC-element(?)/mES-Nanog-ChIP-Seq(GSE11724)/Homer	CACAGCAGGGGG	1e-351	6.32%	1.67%	182	ZNF322(Zf)/HEK293-ZNF322.GFP-ChIP-Seq(GSE58341)/Homer	GAGCCTGGTACTGWGCCTGR	1,00E-138	2.01%	0.42%
71	THRb(NR)/Liver-NR1A2-ChIP-Seq(GSE52613)/Homer	TRAGGTCA	1e-344	50.53%	37.69%	183	At5g04390(C2H2)/col200-At5g04390-DAP-Seq(GSE60143)/Homer	AGTGANDN	1,00E-138	78.01%	70.79%
72	AR-halfsite(NR)/LNCaP-AR-ChIP-Seq(GSE27824)/Homer	CCAGGAACAG	1e-342	44.25%	31.78%	184	ZEB2(Zf)/SNU398-ZEB2-ChIP-Seq(GSE103048)/Homer	GNMCAGGTGTGC	1,00E-137	9.80%	5.59%
73	RXR(NR),DR1/3T3L1-RXR-ChIP-Seq(GSE13511)/Homer	TAGGGCAAAGGTCA	1e-334	12.80%	5.74%	185	SF1(NR)/H295R-Nr5a1-ChIP-Seq(GSE44220)/Homer	CAAGGHCANV	1,00E-136	5.43%	2.43%
74	ZNF692(Zf)/HEK293-ZNF692.GFP-ChIP-Seq(GSE58341)/Homer	GTGGGCCCCA	1e-328	3.18%	0.37%	186	MafK(bZIP)/C2C12-MafK-ChIP-Seq(GSE36030)/Homer	GCTGASTCAGCA	1,00E-135	3.24%	1.07%
75	Rbpj1(?)/Panc1-Rbpj1-ChIP-Seq(GSE47459)/Homer	HTTTCCCASG	1e-325	18.68%	10.12%	187	REF6(Zf)/Arabidopsis-REF6-ChIP-Seq(GSE106942)/Homer	TVCTCTGTTT	1,00E-135	9.38%	5.29%
76	At2g45680(TCP)/colamp-At2g45680-DAP-Seq(GSE60143)/Homer	GTGGGNCCCACNDND	1e-322	2.60%	0.21%	188	Zic2(Zf)/ESC-Zic2-ChIP-Seq(SRP197560)/Homer	CHCAGCRGGRGG	1,00E-132	3.52%	1.25%
77	Bach2(bZIP)/OCILy7-Bach2-ChIP-Seq(GSE44420)/Homer	TGCTGAGTCA	1e-320	5.12%	1.20%	189	IDD5(C2H2)/colamp-IDD5-DAP-Seq(GSE60143)/Homer	TTTTGTCTTTTTBTK	1,00E-128	20.91%	14.94%
78	Sox3(HMG)/NPC-Sox3-ChIP-Seq(GSE33059)/Homer	CCWTTGTY	1e-315	38.08%	26.59%	190	NF-E2(bZIP)/K562-NFE2-ChIP-Seq(GSE31477)/Homer	GATGACTCAGCA	1,00E-128	1.64%	0.29%
79	RARa(NR)/K562-RARa-ChIP-Seq(Encode)/Homer	TTGAMCTTTG	1e-314	43.29%	31.38%	191	STAT6(Stat)/Macrophage-Stat6-ChIP-Seq(GSE38377)/Homer	TTCCKNAGAA	1,00E-127	10.03%	5.90%
80	WT1(Zf)/Kidney-WT1-ChIP-Seq(GSE90016)/Homer	MCTCCCMCRCAB	1e-313	6.54%	1.97%	192	ELF3(ETS)/PDAC-ELF3-ChIP-Seq(GSE64557)/Homer	ANCAGGAAGT	1,00E-127	10.38%	6.16%
81	Klf4(Zf)/mES-Klf4-ChIP-Seq(GSE11431)/Homer	GCCACACCCA	1e-311	4.53%	0.96%	193	REM19(REM)/colamp-REM19-DAP-Seq(GSE60143)/Homer	AAAAAAAA	1,00E-126	21.70%	15.67%
82	ERG(ETS)/VCaP-ERG-ChIP-Seq(GSE14097)/Homer	ACAGGAAGTG	1e-310	19.45%	10.88%	194	SPDEF(ETS)/VCaP-SPDEF-ChIP-Seq(SRA014231)/Homer	ASWTCCTGBT	1,00E-126	13.83%	8.96%
83	bZIP18(bZIP)/colamp-bZIP18-DAP-Seq(GSE60143)/Homer	KGMCAGCTND	1,00E-307	62.82%	50.64%	195	ZNF711(Zf)/SHSY5Y-ZNF711-ChIP-Seq(GSE20673)/Homer	AGGCCTAG	1,00E-123	14.31%	9.41%
84	Twist2(bHLH)/Myoblast-Twist2.Ty1-ChIP-Seq(GSE127998)/Homer	MCAGCTGBYH	1,00E-302	20.89%	12.11%	196	Oct11(POU,Homeobox)/NCIH1048-POU2F3-ChIP-seq(GSE115123)/Homer	GATTTGCATA	1,00E-122	10.43%	6.28%
85	Tal1	CATCTG	1,00E-300	20.81%	12.07%	197	SOL1(CPP)/colamp-SOL1-DAP-Seq(GSE60143)/Homer	ATTTAAATHN	1,00E-121	40.02%	32.67%
86	KLF10(Zf)/HEK293-KLF10.GFP-ChIP-Seq(GSE58341)/Homer	GGGGGTGTGTCC	1,00E-299	5.82%	1.64%	198	Nr5a2(NR)/Pancreas-LRH1-ChIP-Seq(GSE34295)/Homer	BTCAAGGTCA	1,00E-119	8.15%	4.56%
87	Ap4(bHLH)/AML-Tfap4-ChIP-Seq(GSE45738)/Homer	NAHCAGCTGD	1,00E-298	12.22%	5.65%	199	RAV1(RAV)/colamp-RAV1-DAP-Seq(GSE60143)/Homer	TWWTTTCTGTTG	1,00E-118	21.74%	15.89%
88	Tcf12(bHLH)/GM12878-Tcf12-ChIP-Seq(GSE32465)/Homer	VCAGCTGYTG	1,00E-293	8.40%	3.17%	200	bZIP:IRF(bZIP,IRF)/Th17-BatF-ChIP-Seq(GSE39756)/Homer	NAGTTTCABTHTGACTNW	1,00E-117	10.40%	6.33%
89	PPARa(NR),DR1/Liver-Ppara-ChIP-Seq(GSE47954)/Homer	VNAGGKCAAAGGTCA	1,00E-292	14.76%	7.53%	201	WRKY14(WRKY)/colamp-WRKY14-DAP-Seq(GSE60143)/Homer	NCGTTGACTTTN	1,00E-116	13.66%	9.01%
90	TEAD4(TEA)/Tropoblast-Tead4-ChIP-Seq(GSE37350)/Homer	CCWGGAATGY	1,00E-287	15.55%	8.16%	202	AP-2gamma(AP2)/MCF7-TFAP2C-ChIP-Seq(GSE21234)/Homer	SCCTSAGGSCAW	1,00E-115	6.31%	3.25%
91	PPARE(NR),DR1/3T3L1-Pparg-ChIP-Seq(GSE13511)/Homer	TGACCTTTGCCCCA	1,00E-283	11.78%	5.48%	203	OCT:OCT(POU,Homeobox)/NPC-OCT6-ChIP-Seq(GSE43916)/Homer	YATGCATATRCATRT	1,00E-113	3.34%	1.26%
92	TCF4(bHLH)/SHSY5Y-TCF4-ChIP-Seq(GSE96915)/Homer	SMCATCTGKH	1,00E-279	18.58%	10.57%	204	ZBTB9(Zf)/HEK293T-ZBTB9-ChIP-Seq(GSE251687)/Homer	RGCCACGDRR	1,00E-112	19.60%	14.15%
93	EKLF(Zf)/Erythrocyte-Klf1-ChIP-Seq(GSE20478)/Homer	NWGGGTGTGGCY	1,00E-279	3.30%	0.53%	205	Bach1(bZIP)/K562-Bach1-ChIP-Seq(GSE31477)/Homer	AWWNTGCTGAGTCAT	1,00E-112	1.81%	0.42%
94	TEAD2(TEA)/Py2T-Tead2-ChIP-Seq(GSE55709)/Homer	CCWGGAATGY	1,00E-275	10.25%	4.50%	206	WRKY31(WRKY)/colamp-WRKY31-DAP-Seq(GSE60143)/Homer	NCGTTGACTWWK	1,00E-112	16.49%	11.47%
95	TEAD1(TEAD)/HepG2-TEAD1-ChIP-Seq(Encode)/Homer	CYRCATTCCA	1,00E-273	20.09%	11.84%	207	PRDM9(Zf)/Testis-DMC1-ChIP-Seq(GSE35498)/Homer	ADGGYAGYAGCATCT	1,00E-110	4.98%	2.35%
96	HIC1(Zf)/Treg-ZBTB29-ChIP-Seq(GSE99889)/Homer	TGCCAGCB	1,00E-271	20.60%	12.29%	208	PRDM1(Zf)/Hela-PRDM1-ChIP-Seq(GSE31477)/Homer	ACTTTCACTTTC	1,00E-110	10.26%	6.34%
97	BHLHA15(bHLH)/NIH3T3-BHLHB8.HA-ChIP-Seq(GSE119782)/Homer	NAMCAGCTGK	1,00E-267	16.46%	9.07%	209	IDD4(C2H2)/col-IDD4-DAP-Seq(GSE60143)/Homer	TTTGTCTTTWTB	1,00E-108	25.07%	19.10%
98	TF3A(C2H2)/col-TF3A-DAP-Seq(GSE60143)/Homer	NNDDGAGGAGGWNNN	1,00E-255	14.75%	7.91%	210	WRKY71(WRKY)/col-WRKY71-DAP-Seq(GSE60143)/Homer	CKTTGACYWW	1,00E-108	17.75%	12.63%
99	SOX1(HMG)/NPC-SOX1-ChIP-Seq(GSE138215)/Homer	CCATTGTTCB	1,00E-255	42.90%	32.16%	211	WRKY50(WRKY)/col-WRKY50-DAP-Seq(GSE60143)/Homer	NNTTGACTWNNGNNN	1,00E-108	22.44%	16.76%
100	E2A(bHLH),near_PU.1/Bcell-PU.1-ChIP-Seq(GSE21512)/Homer	NVCACCTGBN	1,00E-253	14.73%	7.92%	212	IRF3(IRF)/BMDM-Irf3-ChIP-Seq(GSE67343)/Homer	AGTTTCAKTTTC	1,00E-108	7.23%	4.01%
101	Znf263(Zf)/K562-Znf263-ChIP-Seq(GSE31477)/Homer	CVGTSCTCCC	1,00E-252	14.70%	7.91%	213	Gfi1b(Zf)/HPC7-Gfi1b-ChIP-Seq(GSE22178)/Homer	MAATCACTGC	1,00E-107	10.19%	6.32%
102	NF1-halfsite(CTF)/LNCaP-NF1-ChIP-Seq(Unpublished)/Homer	YTGCCAAG	1,00E-249	19.60%	11.77%	214	WRKY6(WRKY)/colamp-WRKY6-DAP-Seq(GSE60143)/Homer	NCGTTGACTWWD	1,00E-107	19.62%	14.28%
103	GRHL2(CP2)/HBE-GRHL2-ChIP-Seq(GSE46194)/Homer	AAACYKGTTWDACMRGTTTB	1,00E-246	9.39%	4.16%	215	WRKY55(WRKY)/col-WRKY55-DAP-Seq(GSE60143)/Homer	NCGTTGACTT	1,00E-107	24.16%	18.31%
104	PR(NR)/T47D-PR-ChIP-Seq(GSE31130)/Homer	VAGRACAKNCTGTBC	1,00E-246	32.17%	22.53%	216	Stat3(Stat)/mES-Stat3-ChIP-Seq(GSE11431)/Homer	CTTCCGGGAA	1,00E-107	6.27%	3.31%
105	ERb(NR),IR3/Ovary-ERb-ChIP-Seq(GSE203391)/Homer	RGGTCAGGGTGACCT	1,00E-244	4.39%	1.15%	217	STAT1(Stat)/HelaS3-STAT1-ChIP-Seq(GSE12782)/Homer	NATTTCCNGGAAAT	1,00E-106	5.82%	2.99%
106	NeuroG2(bHLH)/Fibroblast-NeuroG2-ChIP-Seq(GSE75910)/Homer	ACCATCTGTT	1,00E-243	19.40%	11.70%	218	TR4(NR),DR1/Hela-TR4-ChIP-Seq(GSE24685)/Homer	GAGGTCAAAGGTCA	1,00E-104	1.80%	0.45%
107	Unknown6/Drosophila-Promoters/Homer	AATTTTAAAA	1,00E-238	28.34%	19.29%	219	WRKY15(WRKY)/col-WRKY15-DAP-Seq(GSE60143)/Homer	VGTTGACTWW	1,00E-104	22.30%	16.73%
108	Zic3(Zf)/mES-Zic3-ChIP-Seq(GSE37889)/Homer	GGCCYCCTGCTGDGH	1,00E-238	4.70%	1.34%	220	WRKY26(WRKY)/colamp-WRKY26-DAP-Seq(GSE60143)/Homer	CGTTGACTWDKN	1,00E-102	12.52%	8.30%
109	NGA4(ABI3VP1)/col-NGA4-DAP-Seq(GSE60143)/Homer	TKNTCAGGTG	1,00E-238	30.74%	21.40%	221	Hand2(bHLH)/Mesoderm-Hand2-ChIP-Seq(GSE61475)/Homer	TGACANARRCCAGRC	1,00E-102	5.52%	2.82%
110	KLF17(Zf)/2cell-Klf17-CutnTag(GSE211845)/Homer	GCYCCGCCCCYH	1,00E-237	5.09%	1.56%	222	WRKY42(WRKY)/colamp-WRKY42-DAP-Seq(GSE60143)/Homer	NAAAGTCAACGN	1,00E-102	8.77%	5.27%
111	Olig2(bHLH)/Neuron-Olig2-ChIP-Seq(GSE30882)/Homer	RCCATMTGTT	1,00E-235	28.06%	19.10%	223	TCX2(CPP)/colamp-TCX2-DAP-Seq(GSE60143)/Homer	NNWWTTYRAAHN	1,00E-101	40.16%	33.41%
112	Tcf21(bHLH)/ArterySmoothMuscle-Tcf21-ChIP-Seq(GSE61369)/Homer	NAACAGCTGG	1,00E-235	9.53%	4.35%	224	AP-2alpha(AP2)/Hela-AP2alpha-ChIP-Seq(GSE31477)/Homer	ATGCCCTGAGGC	1,00E-101	4.71%	2.26%

Legend: The completed list of motifs for unique chromatin FOXA1 regions of MCF-7 cells. The analysis reveals 209 significant motifs (p-value < 1e-100). The FKH motif is the top-ranked motif (p-value < 1e-5999), representing approximately 78% of the unique chromatin FOXA1 regions, while the ERE motif (p-value < 1e-174), representing 4% of these regions.

Supplementary Table 5

Motif analysis of BT474 specific FOXA1 sites (57,753) compared to 257,456 background regions

Rank	Motif Name	Consensus	P-value	FOXA1 regions (%)	Background regions (%)
1	FOXA1(Forkhead)/LNCAP-FOXA1-ChIP-Seq(GSE27824)/Homer	WAAGTAAACA	1e-9208	66.75%	21.37%
2	FOXA1(Forkhead)/MCF7-FOXA1-ChIP-Seq(GSE26831)/Homer	WAAGTAAACA	1e-9136	61.57%	17.58%
3	FOXM1(Forkhead)/MCF7-FOXM1-ChIP-Seq(GSE72977)/Homer	TRTTTACTTW	1e-8449	60.16%	17.89%
4	Foxa2(Forkhead)/Liver-Foxa2-ChIP-Seq(GSE25694)/Homer	CYTGTTTACWYW	1e-7796	53.13%	14.31%
5	Fox:Ebox(Forkhead,bHLH)/Panc1-Foxa2-ChIP-Seq(GSE47459)/Homer	NNNVCTGWGYAAACASN	1e-7560	49.79%	12.61%
6	Foxa3(Forkhead)/Liver-Foxa3-ChIP-Seq(GSE77670)/Homer	BSNTGTTTACWYWGN	1e-5956	33.14%	6.02%
7	PHA-4(Forkhead)/cElegans-Embryos-PHA4-ChIP-Seq(modEncode)/Homer	KTGTTTGC	1e-5015	82.85%	49.44%
8	FoxL2(Forkhead)/Ovary-FoxL2-ChIP-Seq(GSE60858)/Homer	WWTRTAAACAVG	1e-4720	48.53%	17.85%
9	Foxf1(Forkhead)/Lung-Foxf1-ChIP-Seq(GSE77951)/Homer	WWATRTAAACAN	1e-4421	50.00%	19.77%
10	FOXK1(Forkhead)/HEK293-FOXK1-ChIP-Seq(GSE51673)/Homer	NVWTGTTTAC	1e-4188	51.37%	21.52%
11	FoxD3(forkhead)/ZebrafishEmbryo-Foxd3.biotin-ChIP-seq(GSE106676)/Homer	TGTTTAYTTAGC	1e-4065	40.20%	13.69%
12	AP-2gamma(AP2)/MCF7-TFAP2C-ChIP-Seq(GSE21234)/Homer	SCCTSAGGSCAW	1e-4045	27.82%	6.33%
13	FOXK2(Forkhead)/U2OS-FOXK2-ChIP-Seq(E-MTAB-2204)/Homer	SCHTGTTTACAT	1e-3935	36.03%	11.29%
14	Foxo3(Forkhead)/U2OS-Foxo3-ChIP-Seq(E-MTAB-2701)/Homer	DGTAAACA	1e-3769	41.44%	15.32%
15	Foxo1(Forkhead)/RAW-Foxo1-ChIP-Seq(Fan_et_al.)/Homer	CTGTTTAC	1e-3755	53.84%	24.92%
16	AP-2alpha(AP2)/Hela-AP2alpha-ChIP-Seq(GSE31477)/Homer	ATGCCCTGAGGC	1e-3669	22.97%	4.53%
17	FOXP1(Forkhead)/H9-FOXP1-ChIP-Seq(GSE31006)/Homer	NYYTGTTTACHN	1e-3169	28.93%	8.69%
18	NF1(CTF)/LNCAP-NF1-ChIP-Seq(Unpublished)/Homer	CYTGGCABNSTGCCAR	1e-2332	12.66%	1.92%
19	SCL(bHLH)/HPC7-Scl-ChIP-Seq(GSE13511)/Homer	AVCAGCTG	1e-2306	61.68%	38.03%
20	NF1-halfsite(CTF)/LNCaP-NF1-ChIP-Seq(Unpublished)/Homer	YTGCCAAG	1e-1995	34.82%	16.22%
21	Ptf1a(bHLH)/Panc1-Ptf1a-ChIP-Seq(GSE47459)/Homer	ACAGCTGTTN	1e-1970	41.24%	21.37%
22	E2A(bHLH)/proBcell-E2A-ChIP-Seq(GSE21978)/Homer	DNRCAGCTGY	1e-1889	24.45%	9.24%
23	EBF1(EBF)/Near-E2A-ChIP-Seq(GSE21512)/Homer	GTCCCCWGGGGA	1e-1859	17.34%	4.99%
24	HEB(bHLH)/mES-Heb-ChIP-Seq(GSE53233)/Homer	VCAGCTGBNN	1e-1816	30.47%	13.67%
25	GAGA-repeat/SacCer-Promoters/Homer	CTYTCTYTCTCTCTC	1e-1793	64.18%	43.28%
26	Tlx?(NR)/NPC-H3K4me1-ChIP-Seq(GSE16256)/Homer	CTGGCAGSCTGCCA	1e-1753	10.56%	1.83%
27	ZNF416(Zf)/HEK293-ZNF416.GFP-ChIP-Seq(GSE58341)/Homer	WDNCTGGGCA	1e-1736	20.91%	7.42%
28	SeqBias: GA-repeat	GAGAGAGAGA	1e-1731	79.99%	60.96%
29	Ascl1(bHLH)/NeuralTubes-Ascl1-ChIP-Seq(GSE55840)/Homer	NNVVCAGCTGBN	1e-1680	24.72%	10.10%
30	SpliceAcceptor/Homer	TYYYYRCAGG	1e-1552	75.64%	57.03%
31	ERG(ETS)/VCaP-ERG-ChIP-Seq(GSE14097)/Homer	ACAGGAAGTG	1e-1521	27.71%	12.80%
32	Tcf12(bHLH)/GM12878-Tcf12-ChIP-Seq(GSE32465)/Homer	VCAGCTGYTG	1e-1451	15.45%	4.90%
33	Ap4(bHLH)/AML-Tfap4-ChIP-Seq(GSE45738)/Homer	NAHCAGCTGD	1e-1430	19.91%	7.72%
34	Ascl2(bHLH)/ESC-Ascl2-ChIP-Seq(GSE97712)/Homer	SSRGCAGCTGCH	1e-1390	18.88%	7.18%
35	VRN1(ABI3VP1)/col-VRN1-DAP-Seq(GSE60143)/Homer	TTTTTTTTTT	1e-1289	6.74%	0.92%
36	SeqBias: CA-repeat	CACACACACA	1e-1268	74.95%	58.16%
37	Tcf21(bHLH)/ArterySmoothMuscle-Tcf21-ChIP-Seq(GSE61369)/Homer	NAACAGCTGG	1e-1258	16.16%	5.86%
38	MyoG(bHLH)/C2C12-MyoG-ChIP-Seq(GSE36024)/Homer	AACAGCTG	1e-1241	17.10%	6.51%
39	GAGA-repeat/Arabidopsis-Promoters/Homer	CTCTCTCTCY	1e-1226	18.57%	7.52%
40	E2A(bHLH),near_PU.1/Bcell-PU.1-ChIP-Seq(GSE21512)/Homer	NVCACCTGBN	1e-1213	23.80%	11.22%
41	Bcl6(Zf)/Liver-Bcl6-ChIP-Seq(GSE31578)/Homer	NNNCTTTCCAGGAAA	1e-1201	23.64%	11.15%
42	EBF2(EBF)/BrownAdipose-EBF2-ChIP-Seq(GSE97114)/Homer	NABTCCCWDGGGAVH	1e-1190	15.77%	5.83%
43	hTCT(CPE)	BCKTTCTYTTY	1e-1150	39.40%	24.04%
44	Twist2(bHLH)/Myoblast-Twist2.Ty1-ChIP-Seq(GSE127998)/Homer	MCAGCTGBYH	1e-1131	28.77%	15.36%
45	AR-halfsite(NR)/LNCaP-AR-ChIP-Seq(GSE27824)/Homer	CCAGGAACAG	1e-1093	54.68%	38.42%
46	BHLHA15(bHLH)/NIH3T3-BHLHB8.HA-ChIP-Seq(GSE119782)/Homer	NAMCAGCTGK	1e-1092	23.29%	11.37%
47	TCF4(bHLH)/SHSY5Y-TCF4-ChIP-Seq(GSE96915)/Homer	SMCATCTGKH	1e-1086	25.69%	13.21%
48	MyoD(bHLH)/Myotube-MyoD-ChIP-Seq(GSE21614)/Homer	RRCAGCTGYTSY	1e-1071	11.77%	3.76%
49	EHF(ETS)/LoVo-EHF-ChIP-Seq(GSE49402)/Homer	AVCAGGAAGT	1e-1055	24.97%	12.81%
50	RLR1?/SacCer-Promoters/Homer	WTTTTCYYTTTT	1e-1054	16.24%	6.58%
51	TF3A(C2H2)/col-TF3A-DAP-Seq(GSE60143)/Homer	NNDDGAGGAGGWNNN	1e-1046	19.95%	9.15%
52	NGA4(ABI3VP1)/col-NGA4-DAP-Seq(GSE60143)/Homer	TKNTCAGGTG	1e-1031	41.78%	26.89%
53	HIC1(Zf)/Treg-ZBTB29-ChIP-Seq(GSE99889)/Homer	TGCCAGCB	1e-1007	32.28%	18.87%
54	Etv2(ETS)/ES-ER71-ChIP-Seq(GSE59402)/Homer	NNAYTTCCTGHN	1e-1002	17.04%	7.31%
55	Atoh7(bHLH)/Retina-Atoh7-CutnRun(GSE156756)/Homer	KRRCAGCTGGTS	1e-971	12.49%	4.47%
56	ELF3(ETS)/PDAC-ELF3-ChIP-Seq(GSE64557)/Homer	ANCAGGAAGT	1e-962	15.58%	6.47%
57	PU.1-IRF(ETS:IRF)/Bcell-PU.1-ChIP-Seq(GSE21512)/Homer	MGGAAGTGAAAC	1e-962	21.93%	10.97%
58	Atoh1(bHLH)/Cerebellum-Atoh1-ChIP-Seq(GSE22111)/Homer	VNRVCAGCTGGY	1e-961	17.63%	7.87%
59	NeuroG2(bHLH)/Fibroblast-NeuroG2-ChIP-Seq(GSE75910)/Homer	ACCATCTGTT	1e-960	26.10%	14.17%
60	MYRF(MYRF)/CFPAC1-MYRF-ChIP-Seq(GSE145627)/Homer	AGTGCCTGGCAC	1e-933	8.69%	2.40%
61	Erra(NR)/HepG2-Erra-ChIP-Seq(GSE31477)/Homer	CAAAGGTCAG	1e-922	30.28%	17.74%
62	Myf5(bHLH)/GM-Myf5-ChIP-Seq(GSE24852)/Homer	BAACAGCTGT	1e-920	11.80%	4.20%
63	Znf263(Zf)/K562-Znf263-ChIP-Seq(GSE31477)/Homer	CVGTSCTCCC	1e-908	22.94%	11.99%
64	HuR(?)/HEK293-HuR-CLIP-Seq(GSE87887)/Homer	BTTTGGTTTG	1e-903	71.65%	57.31%
65	NFATC2(RHD)/Islets-NFATC2-ChIP-Seq(GSE158496)/Homer	WTTTTCCATTGS	1e-887	37.21%	23.84%
66	ZEB1(Zf)/PDAC-ZEB1-ChIP-Seq(GSE64557)/Homer	VCAGGTRDRY	1e-886	29.36%	17.20%
67	Snail1(Zf)/LS174T-SNAIL1.HA-ChIP-Seq(GSE127183)/Homer	TRCACCTGCY	1e-880	15.47%	6.71%
68	GLIS3(Zf)/Thyroid-Glis3.GFP-ChIP-Seq(GSE103297)/Homer	CTCCCTGGGAGGCCN	1e-848	18.61%	9.06%
69	NeuroD1(bHLH)/Islet-NeuroD1-ChIP-Seq(GSE30298)/Homer	GCCATCTGTT	1e-841	13.65%	5.64%
70	bZIP18(bZIP)/colamp-bZIP18-DAP-Seq(GSE60143)/Homer	KGMCAGCTND	1e-827	72.10%	58.44%
71	EWS:ERG-fusion(ETS)/CADO_ES1-EWS:ERG-ChIP-Seq(SRA014231)/Homer	ATTTCCTGTN	1e-818	14.95%	6.60%
72	ZNF148(Zf)/MDAMB231-ZNF148-ChIP-Seq(GSE147020)/Homer	CCCCTCCCCCAC	1e-797	7.23%	1.94%
73	Olig2(bHLH)/Neuron-Olig2-ChIP-Seq(GSE30882)/Homer	RCCATMTGTT	1e-789	33.95%	21.70%
74	AT5G60130(ABI3VP1)/col-AT5G60130-DAP-Seq(GSE60143)/Homer	WTTYTAAGVAAA	1e-773	44.96%	31.69%
75	Rbpj1(?)/Panc1-Rbpj1-ChIP-Seq(GSE47459)/Homer	HTTTCCCASG	1e-760	22.44%	12.40%
76	KLF10(Zf)/HEK293-KLF10.GFP-ChIP-Seq(GSE58341)/Homer	GGGGGTGTGTCC	1e-739	8.89%	3.00%
77	Unknown-ESC-element(?)/mES-Nanog-ChIP-Seq(GSE11724)/Homer	CACAGCAGGGGG	1e-713	8.12%	2.62%
78	RXR(NR),DR1/3T3L1-RXR-ChIP-Seq(GSE13511)/Homer	TAGGGCAAAGGTCA	1e-711	15.62%	7.53%
79	ZFX(Zf)/mES-Zfx-ChIP-Seq(GSE11431)/Homer	AGGCCTRG	1e-711	17.84%	9.16%
80	PABPC1(?)/MEL-PABC1-CLIP-Seq(GSE69755)/Homer	HAATAAAGNN	1e-704	52.15%	39.12%
81	PR(NR)/T47D-PR-ChIP-Seq(GSE31130)/Homer	VAGRACAKNCTGTBC	1e-703	36.86%	24.89%
82	ZNF322(Zf)/HEK293-ZNF322.GFP-ChIP-Seq(GSE58341)/Homer	GAGCCTGGTACTGWGCCTGR	1e-697	4.25%	0.72%
83	polyAsite/CPSF/Homer	TTCAATAAAG	1e-685	54.05%	41.13%
84	GRHL2(CP2)/HBE-GRHL2-ChIP-Seq(GSE46194)/Homer	AAACYKGTTWDACMRGTTTB	1e-683	11.42%	4.75%
85	Tal1	CATCTG	1e-678	24.14%	14.24%
86	Maz(Zf)/HepG2-Maz-ChIP-Seq(GSE31477)/Homer	GGGGGGGG	1e-660	13.92%	6.55%
87	ETS1(ETS)/Jurkat-ETS1-ChIP-Seq(GSE17954)/Homer	ACAGGAAGTG	1e-659	17.96%	9.51%
88	Bcl11a(Zf)/HSPC-BCL11A-ChIP-Seq(GSE104676)/Homer	TYTGACCASWRG	1e-631	12.28%	5.54%
89	FRS9(ND)/col-FRS9-DAP-Seq(GSE60143)/Homer	RGAGAGAGAAAG	1e-627	4.63%	1.00%
90	KLF14(Zf)/HEK293-KLF14.GFP-ChIP-Seq(GSE58341)/Homer	RGKGGGCGKGGC	1e-625	19.08%	10.56%
91	Elf4(ETS)/BMDM-Elf4-ChIP-Seq(GSE88699)/Homer	ACTTCCKGKT	1e-621	18.34%	10.01%
92	Trl(Zf)/S2-GAGAfactor-ChIP-Seq(GSE40646)/Homer	RGAGAGAG	1e-604	35.71%	24.68%
93	ZEB2(Zf)/SNU398-ZEB2-ChIP-Seq(GSE103048)/Homer	GNMCAGGTGTGC	1e-603	15.50%	7.97%
94	Slug(Zf)/Mesoderm-Snai2-ChIP-Seq(GSE61475)/Homer	SNGCACCTGCHS	1e-592	9.51%	3.85%
95	BPC1(BBRBPC)/colamp-BPC1-DAP-Seq(GSE60143)/Homer	GARGAGAGAGAA	1e-591	11.33%	5.06%
96	Tgif2(Homeobox)/mES-Tgif2-ChIP-Seq(GSE55404)/Homer	TGTCANYT	1e-586	55.51%	43.54%
97	Sp5(Zf)/mES-Sp5.Flag-ChIP-Seq(GSE72989)/Homer	RGKGGGCGGAGC	1e-573	11.72%	5.41%
98	ZNF467(Zf)/HEK293-ZNF467.GFP-ChIP-Seq(GSE58341)/Homer	TGGGGAAGGGCM	1e-571	10.36%	4.49%
99	STAT4(Stat)/CD4-Stat4-ChIP-Seq(GSE22104)/Homer	NYTTCCWGGAAR	1e-570	18.93%	10.78%
100	NF1:FOXA1(CTF,Forkhead)/LNCAP-FOXA1-ChIP-Seq(GSE27824)/Homer	WNTGTTTRYTTTGGCA	1e-564	3.49%	0.60%
101	ELF5(ETS)/T47D-ELF5-ChIP-Seq(GSE30407)/Homer	ACVAGGAAGT	1e-558	15.29%	8.06%
102	Zic3(Zf)/mES-Zic3-ChIP-Seq(GSE37889)/Homer	GGCCYCCTGCTGDGH	1e-554	7.35%	2.63%
103	bZIP52(bZIP)/colamp-bZIP52-DAP-Seq(GSE60143)/Homer	NDNHCAGCTGTCANN	1e-552	25.42%	16.18%
104	PPARE(NR),DR1/3T3L1-Pparg-ChIP-Seq(GSE13511)/Homer	TGACCTTTGCCCCA	1e-545	13.55%	6.84%
105	Tgif1(Homeobox)/mES-Tgif1-ChIP-Seq(GSE55404)/Homer	YTGWCADY	1e-536	52.85%	41.44%
106	HLH-1(bHLH)/cElegans-Embryo-HLH1-ChIP-Seq(modEncode)/Homer	RACAGCTGTTBH	1e-523	15.12%	8.12%
107	IDD5(C2H2)/colamp-IDD5-DAP-Seq(GSE60143)/Homer	TTTTGTCTTTTTBTK	1e-523	19.16%	11.26%
108	SPDEF(ETS)/VCaP-SPDEF-ChIP-Seq(SRA014231)/Homer	ASWTCCTGBT	1e-515	19.33%	11.45%
109	ETV1(ETS)/GIST48-ETV1-ChIP-Seq(GSE22441)/Homer	AACCGGAAGT	1e-512	23.19%	14.61%
110	MRE(NR)/Neuro2A-NR3C2-ChIPnexus(GSE115417)/Homer	GGAACAGAVTGTCCT	1e-510	21.24%	13.02%
111	Sox10(HMG)/SciaticNerve-Sox3-ChIP-Seq(GSE35132)/Homer	CCWTTGTYYB	1e-505	32.66%	22.84%
112	ZNF91(Zf)/HEK-ZNF91.HA-ChIP-Seq(GSE162571)/Homer	RGCMGCCTYC	1e-496	10.75%	5.08%
113	IDD4(C2H2)/col-IDD4-DAP-Seq(GSE60143)/Homer	TTTGTCTTTWTB	1e-490	23.71%	15.21%
114	Stat3+il21(Stat)/CD4-Stat3-ChIP-Seq(GSE19198)/Homer	SVYTTCCNGGAARB	1e-489	12.59%	6.42%
115	ZBTB18(Zf)/HEK293-ZBTB18.GFP-ChIP-Seq(GSE58341)/Homer	AACATCTGGA	1e-483	8.21%	3.41%
116	KLF1(Zf)/HUDEP2-KLF1-CutnRun(GSE136251)/Homer	VDGGGYGGGGCY	1e-482	10.70%	5.10%
117	Zac1(Zf)/Neuro2A-Plagl1-ChIP-Seq(GSE75942)/Homer	HAWGRGGCCM	1e-471	34.82%	25.09%
118	THRb(NR)/Liver-NR1A2-ChIP-Seq(GSE52613)/Homer	TRAGGTCA	1e-461	54.09%	43.48%
119	RBPJ:Ebox(?,bHLH)/Panc1-Rbpj1-ChIP-Seq(GSE47459)/Homer	GGGRAARRGRMCAGMTG	1e-460	4.83%	1.45%
120	REF6(Zf)/Arabidopsis-REF6-ChIP-Seq(GSE106942)/Homer	TVCTCTGTTT	1e-456	11.19%	5.57%
121	PU.1(ETS)/ThioMac-PU.1-ChIP-Seq(GSE21512)/Homer	AGAGGAAGTG	1e-455	8.43%	3.67%
122	BORIS(Zf)/K562-CTCFL-ChIP-Seq(GSE32465)/Homer	CNNBRGCGCCCCCTGSTGGC	1e-450	3.18%	0.65%
123	Rfx6(HTH)/Min6b1-Rfx6.HA-ChIP-Seq(GSE62844)/Homer	TGTTKCCTAGCAACM	1e-443	16.63%	9.78%
124	PPARa(NR),DR1/Liver-Ppara-ChIP-Seq(GSE47954)/Homer	VNAGGKCAAAGGTCA	1e-441	15.33%	8.77%
125	Smad3(MAD)/NPC-Smad3-ChIP-Seq(GSE36673)/Homer	TWGTCTGV	1e-441	42.97%	32.95%
126	ZNF711(Zf)/SHSY5Y-ZNF711-ChIP-Seq(GSE20673)/Homer	AGGCCTAG	1e-439	23.61%	15.54%
127	Sox4(HMG)/proB-Sox4-ChIP-Seq(GSE50066)/Homer	YCTTTGTTCC	1e-437	17.84%	10.78%
128	Jun-AP1(bZIP)/K562-cJun-ChIP-Seq(GSE31477)/Homer	GATGASTCATCN	1e-435	5.43%	1.85%
129	CTCF(Zf)/CD4+-CTCF-ChIP-Seq(Barski_et_al.)/Homer	AYAGTGCCMYCTRGTGGCCA	1e-432	2.60%	0.43%
130	Unknown2/Drosophila-Promoters/Homer	CATCMCTA	1e-429	19.67%	12.33%
131	KLF5(Zf)/LoVo-KLF5-ChIP-Seq(GSE49402)/Homer	DGGGYGKGGC	1e-428	15.33%	8.85%
132	Zfp281(Zf)/ES-Zfp281-ChIP-Seq(GSE81042)/Homer	CCCCTCCCCCAC	1e-425	2.28%	0.32%
133	Tbx5(T-box)/HL1-Tbx5.biotin-ChIP-Seq(GSE21529)/Homer	AGGTGTCA	1e-423	48.70%	38.63%
134	REM19(REM)/colamp-REM19-DAP-Seq(GSE60143)/Homer	AAAAAAAA	1e-418	18.06%	11.10%
135	TEAD(TEA)/Fibroblast-PU.1-ChIP-Seq(Unpublished)/Homer	YCWGGAATGY	1e-416	14.40%	8.21%
136	EWS:FLI1-fusion(ETS)/SK_N_MC-EWS:FLI1-ChIP-Seq(SRA014231)/Homer	VACAGGAAAT	1e-414	10.21%	5.08%
137	TEAD4(TEA)/Tropoblast-Tead4-ChIP-Seq(GSE37350)/Homer	CCWGGAATGY	1e-412	16.48%	9.88%
138	Fosl2(bZIP)/3T3L1-Fosl2-ChIP-Seq(GSE56872)/Homer	NATGASTCABNN	1e-409	7.30%	3.10%
139	Sp1(Zf)/Promoter/Homer	GGCCCCGCCCCC	1e-408	3.51%	0.88%
140	SOX1(HMG)/NPC-SOX1-ChIP-Seq(GSE138215)/Homer	CCATTGTTCB	1e-391	40.82%	31.48%
141	TEAD2(TEA)/Py2T-Tead2-ChIP-Seq(GSE55709)/Homer	CCWGGAATGY	1e-377	10.53%	5.52%
142	ZNF143ISTAF(Zf)/CUTLL-ZNF143-ChIP-Seq(GSE29600)/Homer	ATTTCCCAGVAKSCY	1e-376	4.55%	1.51%
143	Sp2(Zf)/HEK293-Sp2.eGFP-ChIP-Seq(Encode)/Homer	YGGCCCCGCCCC	1e-376	17.10%	10.63%
144	ZBTB9(Zf)/HEK293T-ZBTB9-ChIP-Seq(GSE251687)/Homer	RGCCACGDRR	1e-375	27.63%	19.61%
145	RAV1(RAV)/colamp-RAV1-DAP-Seq(GSE60143)/Homer	TWWTTTCTGTTG	1e-371	20.25%	13.28%
146	Ets1-distal(ETS)/CD4+-PolII-ChIP-Seq(Barski_et_al.)/Homer	MACAGGAAGT	1e-370	5.77%	2.27%
147	OCT:OCT(POU,Homeobox)/NPC-OCT6-ChIP-Seq(GSE43916)/Homer	YATGCATATRCATRT	1e-368	3.64%	1.03%
148	Sox3(HMG)/NPC-Sox3-ChIP-Seq(GSE33059)/Homer	CCWTTGTY	1e-364	35.34%	26.69%
149	SeqBias: TA-repeat	TATATATATA	1e-361	66.50%	57.32%
150	FOXA1:AR(Forkhead,NR)/LNCAP-AR-ChIP-Seq(GSE27824)/Homer	AGTAAACAAAAAAGAACAND	1e-355	3.26%	0.86%
151	MafB(bZIP)/BMM-Mafb-ChIP-Seq(GSE75722)/Homer	WNTGCTGASTCAGCANWTTY	1e-352	7.57%	3.53%
152	Hand2(bHLH)/Mesoderm-Hand2-ChIP-Seq(GSE61475)/Homer	TGACANARRCCAGRC	1e-349	7.18%	3.27%
153	Sox21(HMG)/ESC-SOX21-ChIP-Seq(GSE110505)/Homer	BCCWTTGTBYKV	1e-347	33.93%	25.58%
154	Fra2(bZIP)/Striatum-Fra2-ChIP-Seq(GSE43429)/Homer	GGATGACTCATC	1e-347	10.60%	5.74%
155	Klf9(Zf)/GBM-Klf9-ChIP-Seq(GSE62211)/Homer	GCCACRCCCACY	1e-343	4.39%	1.52%
156	KLF6(Zf)/PDAC-KLF6-ChIP-Seq(GSE64557)/Homer	MKGGGYGTGGCC	1e-339	12.53%	7.26%
157	GABPA(ETS)/Jurkat-GABPa-ChIP-Seq(GSE17954)/Homer	RACCGGAAGT	1e-337	15.38%	9.53%
158	RUNX-AML(Runt)/CD4+-PolII-ChIP-Seq(Barski_et_al.)/Homer	GCTGTGGTTW	1e-336	12.69%	7.40%
159	Gfi1b(Zf)/HPC7-Gfi1b-ChIP-Seq(GSE22178)/Homer	MAATCACTGC	1e-333	12.40%	7.19%
160	KLF3(Zf)/MEF-Klf3-ChIP-Seq(GSE44748)/Homer	NRGCCCCRCCCHBNN	1e-332	6.03%	2.57%
161	Oct4(POU,Homeobox)/mES-Oct4-ChIP-Seq(GSE11431)/Homer	ATTTGCATAW	1e-331	13.88%	8.37%
162	GATA(Zf),IR3/iTreg-Gata3-ChIP-Seq(GSE20898)/Homer	NNNNNBAGATAWYATCTVHN	1e-331	6.44%	2.85%
163	Klf15(Zf)/Liver-Klf15-ChIP-Seq(GSE166083)/Homer	RGGGGGCGGGGC	1e-325	12.72%	7.50%
164	AP-1(bZIP)/ThioMac-PU.1-ChIP-Seq(GSE21512)/Homer	VTGACTCATC	1e-325	15.54%	9.75%
165	ZNF189(Zf)/HEK293-ZNF189.GFP-ChIP-Seq(GSE58341)/Homer	TGGAACAGMA	1e-322	12.16%	7.08%
166	Mesp1(bHLH)/ESC-Mesp1-ChIP-Seq(GSE165102)/Homer	RCCATTTGBY	1e-314	11.22%	6.41%
167	E-box/Drosophila-Promoters/Homer	AACAGCTGTTHN	1e-313	4.74%	1.82%
168	Nr5a2(NR)/Pancreas-LRH1-ChIP-Seq(GSE34295)/Homer	BTCAAGGTCA	1e-312	10.59%	5.94%
169	Sox9(HMG)/Limb-SOX9-ChIP-Seq(GSE73225)/Homer	AGGVNCCTTTGT	1e-311	15.76%	10.04%
170	Meis1(Homeobox)/MastCells-Meis1-ChIP-Seq(GSE48085)/Homer	VGCTGWCAVB	1e-311	26.27%	19.06%
171	Zic(Zf)/Cerebellum-ZIC1.2-ChIP-Seq(GSE60731)/Homer	CCTGCTGAGH	0,00E+00	11.41%	6.59%
172	WT1(Zf)/Kidney-WT1-ChIP-Seq(GSE90016)/Homer	MCTCCCMCRCAB	0,00E+00	7.08%	3.39%
173	Unknown6/Drosophila-Promoters/Homer	AATTTTAAAA	1,00E-307	21.19%	14.66%
174	Stat3(Stat)/mES-Stat3-ChIP-Seq(GSE11431)/Homer	CTTCCGGGAA	1,00E-304	8.62%	4.52%
175	Oct2(POU,Homeobox)/Bcell-Oct2-ChIP-Seq(GSE21512)/Homer	ATATGCAAAT	1,00E-302	9.17%	4.93%
176	Sox15(HMG)/CPA-Sox15-ChIP-Seq(GSE62909)/Homer	RAACAATGGN	1,00E-302	23.04%	16.32%
177	MafA(bZIP)/Islet-MafA-ChIP-Seq(GSE30298)/Homer	TGCTGACTCA	1,00E-298	12.62%	7.62%
178	Zscan4c(Zf)/ESC-Zscan4c-ChIP-seq(GSE140619)/Homer	ATRTGTGCAC	1,00E-298	7.85%	3.98%
179	THRb(NR)/HepG2-THRb.Flag-ChIP-Seq(Encode)/Homer	GGTCACCTGAGGTCA	1,00E-296	7.40%	3.68%
180	RARa(NR)/K562-RARa-ChIP-Seq(Encode)/Homer	TTGAMCTTTG	1,00E-292	42.40%	34.21%
181	PIF4(bHLH)/Seedling-PIF4-ChIP-Seq(GSE35315)/Homer	NNBCACGTGN	1,00E-291	19.26%	13.15%
182	AT5G25475(ABI3VP1)/col-AT5G25475-DAP-Seq(GSE60143)/Homer	RNNRNCAAGCADNDB	1,00E-290	21.63%	15.20%
183	WIP5(C2H2)/colamp-WIP5-DAP-Seq(GSE60143)/Homer	TDTTCTCMAGGT	1,00E-290	20.55%	14.27%
184	JunB(bZIP)/DendriticCells-Junb-ChIP-Seq(GSE36099)/Homer	RATGASTCAT	1,00E-283	12.16%	7.36%
185	Nr5a2(NR)/mES-Nr5a2-ChIP-Seq(GSE19019)/Homer	BTCAAGGTCA	1,00E-282	7.66%	3.93%
186	NPAS2(bHLH)/Liver-NPAS2-ChIP-Seq(GSE39860)/Homer	KCCACGTGAC	1,00E-279	17.17%	11.49%
187	Bach2(bZIP)/OCILy7-Bach2-ChIP-Seq(GSE44420)/Homer	TGCTGAGTCA	1,00E-278	4.04%	1.51%
188	PRDM9(Zf)/Testis-DMC1-ChIP-Seq(GSE35498)/Homer	ADGGYAGYAGCATCT	1,00E-277	6.20%	2.93%
189	TEAD1(TEAD)/HepG2-TEAD1-ChIP-Seq(Encode)/Homer	CYRCATTCCA	1,00E-276	19.17%	13.22%
190	BATF(bZIP)/Th17-BATF-ChIP-Seq(GSE39756)/Homer	DATGASTCAT	1,00E-273	14.52%	9.35%
191	hINR(CPE)	SBCABW	1,00E-271	17.03%	11.45%
192	RUNX1(Runt)/Jurkat-RUNX1-ChIP-Seq(GSE29180)/Homer	AAACCACARM	1,00E-271	18.00%	12.27%
193	Atf3(bZIP)/GBM-ATF3-ChIP-Seq(GSE33912)/Homer	DATGASTCATHN	1,00E-270	14.52%	9.36%
194	Oct11(POU,Homeobox)/NCIH1048-POU2F3-ChIP-seq(GSE115123)/Homer	GATTTGCATA	1,00E-269	9.81%	5.62%
195	STZ(C2H2)/colamp-STZ-DAP-Seq(GSE60143)/Homer	HNBTCACT	1,00E-269	80.20%	73.28%
196	HNF4a(NR),DR1/HepG2-HNF4a-ChIP-Seq(GSE25021)/Homer	CARRGKBCAAAGTYCA	1,00E-266	6.76%	3.38%
197	SF1(NR)/H295R-Nr5a1-ChIP-Seq(GSE44220)/Homer	CAAGGHCANV	1,00E-265	6.51%	3.21%
198	ZNF7(Zf)/HepG2-ZNF7.Flag-ChIP-Seq(Encode)/Homer	CTGCCWVCTTTTRTA	1,00E-261	12.27%	7.61%
199	Zic2(Zf)/ESC-Zic2-ChIP-Seq(SRP197560)/Homer	CHCAGCRGGRGG	1,00E-260	5.45%	2.49%
200	BMAL1(bHLH)/Liver-Bmal1-ChIP-Seq(GSE39860)/Homer	GNCACGTG	1,00E-260	28.46%	21.63%
201	EBF(EBF)/proBcell-EBF-ChIP-Seq(GSE21978)/Homer	DGTCCCYRGGGA	1,00E-259	2.98%	0.95%
202	PRDM15(Zf)/ESC-Prdm15-ChIP-Seq(GSE73694)/Homer	YCCDNTCCAGGTTTT	1,00E-258	16.48%	11.12%
203	AZF1(C2H2)/colamp-AZF1-DAP-Seq(GSE60143)/Homer	DKSWCACT	1,00E-257	73.36%	66.03%
204	TEAD3(TEA)/HepG2-TEAD3-ChIP-Seq(Encode)/Homer	TRCATTCCAG	1,00E-254	23.16%	16.93%
205	BPC6(BBRBPC)/col-BPC6-DAP-Seq(GSE60143)/Homer	YTYTCTCTCTCTCTA	1,00E-254	1.16%	0.12%
206	Pax8(Paired,Homeobox)/Thyroid-Pax8-ChIP-Seq(GSE26938)/Homer	GTCATGCHTGRCTGS	1,00E-252	4.31%	1.78%
207	STAT5(Stat)/mCD4+-Stat5-ChIP-Seq(GSE12346)/Homer	RTTTCTNAGAAA	1,00E-250	6.20%	3.06%
208	MNT(bHLH)/HepG2-MNT-ChIP-Seq(Encode)/Homer	DGCACACGTG	1,00E-249	16.67%	11.35%
209	Nkx3.1(Homeobox)/LNCaP-Nkx3.1-ChIP-Seq(GSE28264)/Homer	AAGCACTTAA	1,00E-243	41.06%	33.64%
210	ZNF341(Zf)/EBV-ZNF341-ChIP-Seq(GSE113194)/Homer	GGAACAGCCG	1,00E-235	9.45%	5.58%
211	At5g04390(C2H2)/col200-At5g04390-DAP-Seq(GSE60143)/Homer	AGTGANDN	1,00E-232	79.09%	72.59%
212	RUNX2(Runt)/PCa-RUNX2-ChIP-Seq(GSE33889)/Homer	NWAACCACADNN	1,00E-226	14.41%	9.67%
213	PRDM1(Zf)/Hela-PRDM1-ChIP-Seq(GSE31477)/Homer	ACTTTCACTTTC	1,00E-226	10.06%	6.12%
214	THRa(NR)/C17.2-THRa-ChIP-Seq(GSE38347)/Homer	GGTCANYTGAGGWCA	1,00E-226	5.87%	2.95%
215	Klf4(Zf)/mES-Klf4-ChIP-Seq(GSE11431)/Homer	GCCACACCCA	1,00E-223	4.42%	1.96%
216	Fra1(bZIP)/BT549-Fra1-ChIP-Seq(GSE46166)/Homer	NNATGASTCATH	1,00E-222	12.66%	8.25%
217	bZIP:IRF(bZIP,IRF)/Th17-BatF-ChIP-Seq(GSE39756)/Homer	NAGTTTCABTHTGACTNW	1,00E-221	8.96%	5.29%
218	Fos(bZIP)/TSC-Fos-ChIP-Seq(GSE110950)/Homer	NDATGASTCAYN	1,00E-218	13.43%	8.93%
219	STAT1(Stat)/HelaS3-STAT1-ChIP-Seq(GSE12782)/Homer	NATTTCCNGGAAAT	1,00E-213	6.07%	3.17%
220	FUS3(ABI3VP1)/col-FUS3-DAP-Seq(GSE60143)/Homer	DNNWTNTGCATGKNN	1,00E-210	17.63%	12.59%
221	ERRg(NR)/Kidney-ESRRG-ChIP-Seq(GSE104905)/Homer	GTGACCTTGRVN	1,00E-201	12.20%	8.06%
222	GRF9(GRF)/colamp-GRF9-DAP-Seq(GSE60143)/Homer	NWCTGACANNNNNNN	1,00E-199	16.05%	11.34%
223	MafK(bZIP)/C2C12-MafK-ChIP-Seq(GSE36030)/Homer	GCTGASTCAGCA	1,00E-196	3.40%	1.41%
224	FXR(NR),ER2/Liver-FXR-ChIP-Seq(GSE133700)/Homer	NTGACCYMKRGGTCA	1,00E-194	6.90%	3.90%
225	Twist(bHLH)/HMLE-TWIST1-ChIP-Seq(Chang_et_al)/Homer	VCAKCTGGNNNCCAGMTGBN	1,00E-193	1.90%	0.53%
226	GATA(Zf),IR4/iTreg-Gata3-ChIP-Seq(GSE20898)/Homer	NAGATWNBNATCTNN	1,00E-191	3.93%	1.77%
227	VIP1(bZIP)/col-VIP1-DAP-Seq(GSE60143)/Homer	NTTGACAGCTGTCAN	1,00E-188	3.30%	1.38%
228	NFAT(RHD)/Jurkat-NFATC1-ChIP-Seq(Jolma_et_al.)/Homer	ATTTTCCATT	1,00E-187	16.83%	12.14%
229	Pitx1:Ebox(Homeobox,bHLH)/Hindlimb-Pitx1-ChIP-Seq(GSE41591)/Homer	YTAATTRAWWCCAGATGT	1,00E-187	5.33%	2.78%
230	GT2(Trihelix)/colamp-GT2-DAP-Seq(GSE60143)/Homer	AMGGTAAAWWWN	1,00E-186	33.27%	27.12%
231	EAR2(NR)/K562-NR2F6-ChIP-Seq(Encode)/Homer	NRBCARRGGTCA	1,00E-185	17.63%	12.87%
232	STAT6(Stat)/Macrophage-Stat6-ChIP-Seq(GSE38377)/Homer	TTCCKNAGAA	1,00E-181	9.38%	5.94%
233	IRF3(IRF)/BMDM-Irf3-ChIP-Seq(GSE67343)/Homer	AGTTTCAKTTTC	1,00E-180	6.21%	3.46%
234	Hoxd10(Homeobox)/ChickenMSG-Hoxd10.Flag-ChIP-Seq(GSE86088)/Homer	GGCMATGAAA	1,00E-180	27.54%	21.88%
235	Bapx1(Homeobox)/VertebralCol-Bapx1-ChIP-Seq(GSE36672)/Homer	TTRAGTGSYK	1,00E-179	36.97%	30.74%
236	HOXD13(Homeobox)/Chicken-Hoxd13-ChIP-Seq(GSE38910)/Homer	NCYAATAAAA	1,00E-177	31.46%	25.57%
237	Zfp809(Zf)/ES-Zfp809-ChIP-Seq(GSE70799)/Homer	GGGGCTYGKCTGGGA	1,00E-176	2.34%	0.82%
238	KLF17(Zf)/2cell-Klf17-CutnTag(GSE211845)/Homer	GCYCCGCCCCYH	1,00E-175	6.10%	3.41%
239	COUP-TFII(NR)/K562-NR2F1-ChIP-Seq(Encode)/Homer	GKBCARAGGTCA	1,00E-175	20.07%	15.15%
240	RBFox2(?)/Heart-RBFox2-CLIP-Seq(GSE57926)/Homer	TGCATGCA	1,00E-175	34.63%	28.59%
241	Sox2(HMG)/mES-Sox2-ChIP-Seq(GSE11431)/Homer	BCCATTGTTC	1,00E-173	18.21%	13.53%
242	GTL1(Trihelix)/colamp-GTL1-DAP-Seq(GSE60143)/Homer	WWTTTACCKY	1,00E-168	31.74%	25.98%
243	STAT6(Stat)/CD4-Stat6-ChIP-Seq(GSE22104)/Homer	ABTTCYYRRGAA	1,00E-167	9.42%	6.09%
244	SGR5(C2H2)/colamp-SGR5-DAP-Seq(GSE60143)/Homer	TTTGTCTTTTTT	1,00E-166	15.24%	11.01%
245	PIF5ox(bHLH)/Arabidopsis-PIF5ox-ChIP-Seq(GSE35062)/Homer	BCACGTGVDN	1,00E-165	15.35%	11.11%
246	Tbx20(T-box)/Heart-Tbx20-ChIP-Seq(GSE29636)/Homer	GGTGYTGACAGS	1,00E-165	4.07%	1.99%
247	ETV4(ETS)/HepG2-ETV4-ChIP-Seq(ENCODE)/Homer	ACCGGAAGTG	1,00E-165	17.43%	12.95%
248	IRF8(IRF)/BMDM-IRF8-ChIP-Seq(GSE77884)/Homer	GRAASTGAAAST	1,00E-163	5.53%	3.06%
249	PU.1:IRF8(ETS:IRF)/pDC-Irf8-ChIP-Seq(GSE66899)/Homer	GGAAGTGAAAST	1,00E-162	3.28%	1.47%
250	GATA3(Zf),DR4/iTreg-Gata3-ChIP-Seq(GSE20898)/Homer	AGATGKDGAGATAAG	1,00E-160	3.72%	1.77%
251	DAG2(C2C2dof)/col-DAG2-DAP-Seq(GSE60143)/Homer	WWTTHACTTTTT	1,00E-160	41.62%	35.56%
252	WRKY8(WRKY)/colamp-WRKY8-DAP-Seq(GSE60143)/Homer	CGTTGACTTT	1,00E-159	3.82%	1.85%
253	NFkB-p65(RHD)/GM12787-p65-ChIP-Seq(GSE19485)/Homer	WGGGGATTTCCC	1,00E-158	6.79%	4.06%
254	PRDM10(Zf)/HEK293-PRDM10.eGFP-ChIP-Seq(Encode)/Homer	TGGTACATTCCA	1,00E-156	8.39%	5.35%
255	Ronin(THAP)/ES-Thap11-ChIP-Seq(GSE51522)/Homer	RACTACAACTCCCAGVAKGC	1,00E-155	0.65%	0.06%
256	EKLF(Zf)/Erythrocyte-Klf1-ChIP-Seq(GSE20478)/Homer	NWGGGTGTGGCY	1,00E-154	2.47%	0.98%
257	ERF15(AP2EREBP)/colamp-ERF15-DAP-Seq(GSE60143)/Homer	WDHAGCMGCCAT	1,00E-154	23.45%	18.51%
258	SOL1(CPP)/colamp-SOL1-DAP-Seq(GSE60143)/Homer	ATTTAAATHN	1,00E-153	33.60%	27.98%
259	NLP7(RWPRK)/col-NLP7-DAP-Seq(GSE60143)/Homer	TGRCCYTTCR	1,00E-151	28.87%	23.57%
260	Rfx1(HTH)/NPC-H3K4me1-ChIP-Seq(GSE16256)/Homer	KGTTGCCATGGCAA	1,00E-147	3.08%	1.40%
261	SpiB(ETS)/OCILY3-SPIB-ChIP-Seq(GSE56857)/Homer	AAAGRGGAAGTG	1,00E-143	4.19%	2.20%
262	OCT4-SOX2-TCF-NANOG(POU,Homeobox,HMG)/mES-Oct4-ChIP-Seq(GSE11431)/Homer	ATTTGCATAACAATG	1,00E-141	5.18%	2.94%
263	Cdx2(Homeobox)/mES-Cdx2-ChIP-Seq(GSE14586)/Homer	GYMATAAAAH	1,00E-141	22.40%	17.75%
264	Hoxa9(Homeobox)/ChickenMSG-Hoxa9.Flag-ChIP-Seq(GSE86088)/Homer	RGCAATNAAA	1,00E-141	55.62%	49.77%
265	Oct6(POU,Homeobox)/NPC-Pou3f1-ChIP-Seq(GSE35496)/Homer	WATGCAAATGAG	1,00E-141	10.34%	7.10%
266	Esrrb(NR)/mES-Esrrb-ChIP-Seq(GSE11431)/Homer	KTGACCTTGA	1,00E-137	9.61%	6.54%
267	BIM2(bHLH)/col-BIM2-DAP-Seq(GSE60143)/Homer	NNNNCACGTGNN	1,00E-136	14.38%	10.62%
268	At3g45610(C2C2dof)/col-At3g45610-DAP-Seq(GSE60143)/Homer	TWACTTTTTS	1,00E-136	36.60%	31.17%
269	Hoxa13(Homeobox)/ChickenMSG-Hoxa13.Flag-ChIP-Seq(GSE86088)/Homer	CYHATAAAAN	1,00E-133	55.84%	50.16%
270	bZIP69(bZIP)/col-bZIP69-DAP-Seq(GSE60143)/Homer	GACAGCTGKCAW	1,00E-132	1.88%	0.69%
271	Egr1(Zf)/K562-Egr1-ChIP-Seq(GSE32465)/Homer	TGCGTGGGYG	1,00E-130	7.16%	4.58%
272	AT5G63260(C3H)/col-AT5G63260-DAP-Seq(GSE60143)/Homer	RAAAAAGTRA	1,00E-130	50.67%	45.06%
273	ASHR1(ND)/col-ASHR1-DAP-Seq(GSE60143)/Homer	NTGGTGAN	1,00E-127	18.62%	14.53%
274	NRF1(NRF)/MCF7-NRF1-ChIP-Seq(Unpublished)/Homer	CTGCGCATGCGC	1,00E-127	1.44%	0.45%
275	Smad4(MAD)/ESC-SMAD4-ChIP-Seq(GSE29422)/Homer	VBSYGTCTGG	1,00E-126	22.01%	17.63%
276	Hoxd12(Homeobox)/ChickenMSG-Hoxd12.Flag-ChIP-Seq(GSE86088)/Homer	HDGYAATGAAAN	1,00E-126	42.01%	36.61%
277	Unknown(Homeobox)/Limb-p300-ChIP-Seq/Homer	SSCMATWAAA	1,00E-125	19.54%	15.40%
278	AT2G28810(C2C2dof)/colamp-AT2G28810-DAP-Seq(GSE60143)/Homer	VAAAAAGTWA	1,00E-124	47.94%	42.48%
279	ZNF415(Zf)/HEK293-ZNF415.GFP-ChIP-Seq(GSE58341)/Homer	GRTGMTRGAGCC	1,00E-123	6.69%	4.26%
280	ETS:E-box(ETS,bHLH)/HPC7-Scl-ChIP-Seq(GSE22178)/Homer	AGGAARCAGCTG	1,00E-123	1.47%	0.48%
281	At1g14580(C2H2)/colamp-At1g14580-DAP-Seq(GSE60143)/Homer	CASAAAAMGACAAAA	1,00E-123	4.91%	2.87%
282	p63(p53)/Keratinocyte-p63-ChIP-Seq(GSE17611)/Homer	NNDRCATGYCYNRRCATGYH	1,00E-123	5.42%	3.26%
283	Foxh1(Forkhead)/hESC-FOXH1-ChIP-Seq(GSE29422)/Homer	NNTGTGGATTSS	1,00E-122	15.93%	12.18%
284	At5g52660(MYBrelated)/colamp-At5g52660-DAP-Seq(GSE60143)/Homer	HAAAAATATCTW	1,00E-122	14.23%	10.68%
285	NPAS(bHLH)/Liver-NPAS-ChIP-Seq(GSE39860)/Homer	NVCACGTG	1,00E-122	25.47%	20.90%
286	c-Jun-CRE(bZIP)/K562-cJun-ChIP-Seq(GSE31477)/Homer	ATGACGTCATCY	1,00E-120	4.82%	2.81%
287	MafF(bZIP)/HepG2-MafF-ChIP-Seq(GSE31477)/Homer	HWWGTCAGCAWWTTT	1,00E-119	7.01%	4.57%
288	Max(bHLH)/K562-Max-ChIP-Seq(GSE31477)/Homer	RCCACGTGGYYN	1,00E-118	9.65%	6.77%
289	AT5G02460(C2C2dof)/col-AT5G02460-DAP-Seq(GSE60143)/Homer	CHCCTTTT	1,00E-117	42.63%	37.41%
290	Lola-I(Zf)/Embryo-LolaI-ChIP-Seq(GSE200870)/Homer	VAAAGCTY	1,00E-117	9.77%	6.88%
291	Rfx5(HTH)/GM12878-Rfx5-ChIP-Seq(GSE31477)/Homer	SCCTAGCAACAG	1,00E-117	5.27%	3.19%
292	Pit1(Homeobox)/GCrat-Pit1-ChIP-Seq(GSE58009)/Homer	ATGMATATDC	1,00E-116	27.65%	23.05%
293	AT2G28920(ND)/col-AT2G28920-DAP-Seq(GSE60143)/Homer	WAGATATTTWTW	1,00E-115	9.19%	6.41%
294	ARE(NR)/LNCAP-AR-ChIP-Seq(GSE27824)/Homer	RGRACASNSTGTYCYB	1,00E-115	3.24%	1.68%
295	PGR(NR)/EndoStromal-PGR-ChIP-Seq(GSE69539)/Homer	AAGAACATWHTGTTC	1,00E-114	5.01%	3.01%
296	TCX2(CPP)/colamp-TCX2-DAP-Seq(GSE60143)/Homer	NNWWTTYRAAHN	1,00E-114	33.98%	29.11%
297	AT5G47660(Trihelix)/colamp-AT5G47660-DAP-Seq(GSE60143)/Homer	AWTTTTACCG	1,00E-111	38.06%	33.10%
298	COUP-TFII(NR)/Artia-Nr2f2-ChIP-Seq(GSE46497)/Homer	AGRGGTCA	1,00E-110	23.00%	18.83%
299	AT5G23930(mTERF)/col-AT5G23930-DAP-Seq(GSE60143)/Homer	GGCGGCTG	1,00E-110	16.03%	12.46%
300	Sox6(HMG)/Myotubes-Sox6-ChIP-Seq(GSE32627)/Homer	CCATTGTTNY	1,00E-110	32.70%	27.98%
301	Brn1(POU,Homeobox)/NPC-Brn1-ChIP-Seq(GSE35496)/Homer	TATGCWAATBAV	1,00E-109	8.02%	5.49%
302	OBP3(C2C2dof)/col-OBP3-DAP-Seq(GSE60143)/Homer	NYWACTTTTT	1,00E-109	60.11%	55.01%
303	ZNF519(Zf)/HEK293-ZNF519.GFP-ChIP-Seq(GSE58341)/Homer	GAGSCCGAGC	1,00E-109	2.10%	0.92%
304	ZSCAN22(Zf)/HEK293-ZSCAN22.GFP-ChIP-Seq(GSE58341)/Homer	SMCAGTCWGAKGGAGGAGGC	1,00E-109	0.87%	0.20%
305	AT2G31460(REMB3)/col-AT2G31460-DAP-Seq(GSE60143)/Homer	WNWARWDGAAATGAT	1,00E-109	6.97%	4.63%
306	RUNX(Runt)/HPC7-Runx1-ChIP-Seq(GSE22178)/Homer	SAAACCACAG	1,00E-109	12.03%	8.93%
307	bHLH80(bHLH)/col-bHLH80-DAP-Seq(GSE60143)/Homer	NNNNDCAASTTGHNN	1,00E-108	14.45%	11.08%
308	GATA3(Zf),DR8/iTreg-Gata3-ChIP-Seq(GSE20898)/Homer	AGATSTNDNNDSAGATAASN	1,00E-107	2.57%	1.24%
309	WUS1(Homeobox)/colamp-WUS1-DAP-Seq(GSE60143)/Homer	CAWTCATTCA	1,00E-106	10.15%	7.33%
310	WRKY28(WRKY)/col-WRKY28-DAP-Seq(GSE60143)/Homer	BGTTGACTWH	1,00E-105	26.19%	21.90%
311	Hoxc13(Homeobox)/EB-Hoxc13.HA-ChIP-Seq(GSE142377)/Homer	TTTTATKRGN	1,00E-105	39.27%	34.42%
312	GFY-Staf(?,Zf)/Promoter/Homer	RACTACAATTCCCAGAAKGC	1,00E-102	0.94%	0.25%
313	NFAT:AP1(RHD,bZIP)/Jurkat-NFATC1-ChIP-Seq(Jolma_et_al.)/Homer	SARTGGAAAAWRTGAGTCAB	1,00E-102	3.13%	1.66%
314	X-box(HTH)/NPC-H3K4me1-ChIP-Seq(GSE16256)/Homer	GGTTGCCATGGCAA	1,00E-102	1.82%	0.76%
315	ETS:RUNX(ETS,Runt)/Jurkat-RUNX1-ChIP-Seq(GSE17954)/Homer	RCAGGATGTGGT	1,00E-102	1.31%	0.45%
316	CDF3(C2C2dof)/colamp-CDF3-DAP-Seq(GSE60143)/Homer	AAAAGTRM	1,00E-100	38.42%	33.71%

Legend: The complete list of motifs for unique chromatin FOXA1 regions for BT474 cells. Motif analysis reveals 316 significant motifs (p-value < 1e-100). The FKH motif ranks within the top motifs (p-value < 1e-9136), accounting for 61% of the unique chromatin FOXA1 regions. Notably, the ERE motif is not identified in these regions with a significant p-value threshold of 1e-100.

Supplementary Table 6

List of oligos used for Realtime PCR

cDNA oligos	Primer sequence	Purification
CCNE2 FW	GATGGTGCTTGCAGTGAAGA	DESALT
CCNE2 REV	GGAGAAAGAGATTTAGCCAGGA	DESALT
MELK FW	GATGAGGATTGCGTGACAGA	DESALT
MELK REV	AGGTAGGTGGCTGTGAGGTG	DESALT
NDC80 FW	TCCTCATACATGGCCTCACA	DESALT
NDC80 REV	AGGCTGCCCATCATCAAATA	DESALT
MYC FW	AGCGACTCTGAGGAGGAACA	DESALT
MYC REV	CTCTGACCTTTTGCCAGGAG	DESALT
CCND1 FW	TGGAGGTCTGCGAGGAACAGAA	DESALT
CCND1 REV	TGCAGGCGGCTCTTTTTCA	DESALT
TBP FW	GCCAGCTTCGGAGAGTTCTG	DESALT
TBP REV	GCACGAAGTGCAATGGTCTTT	DESALT
UBC FW	ATTTGGGTCGCGGTTCTTG	DESALT
UBC REV	TGCCTTGACATTCTCGATGGT	DESALT
XBP1 FW	GCGCCTCACGCACCTG	DESALT
XBP1 REV	GCTGCTACTCTGTTTTTCAGTTTCC	DESALT
**ChIP oligos**	**Primer sequence**	**Purification**
CCND1 FW	CTCAGGGATGGCTTTTGGG	DESALT
CCND1 REV	AAACTCCCCTGTAGTCCGTG	DESALT
NDC80 FW	GGTGTTTTGATGACCGCTGT	DESALT
NDC80 REV	TTGGCGCCATCACTTTCTTC	DESALT
MELK FW	CTTCATACTTGTTGCTAGGTGGT	DESALT
MELK REV	TGCTATGAGAAACAGGTGGC	DESALT
CCNE2 FW	CCACCATGCCCGGCTAAAA	DESALT
CCNE2 REV	CAGGTTCCCACTGTGTTACT	DESALT
XBP1 FW	GTGGGTTGGAAAAGAGGTGG	DESALT
XBP1 REV	CACCTCCAGACTTCTCCCAA	DESALT
CCND2 FW	CACCCCTCTTTGCACTGTTC	DESALT
CCND2 REV	AAGTCATTCAGGCCCCTCTC	DESALT

Figure Supplementary 1

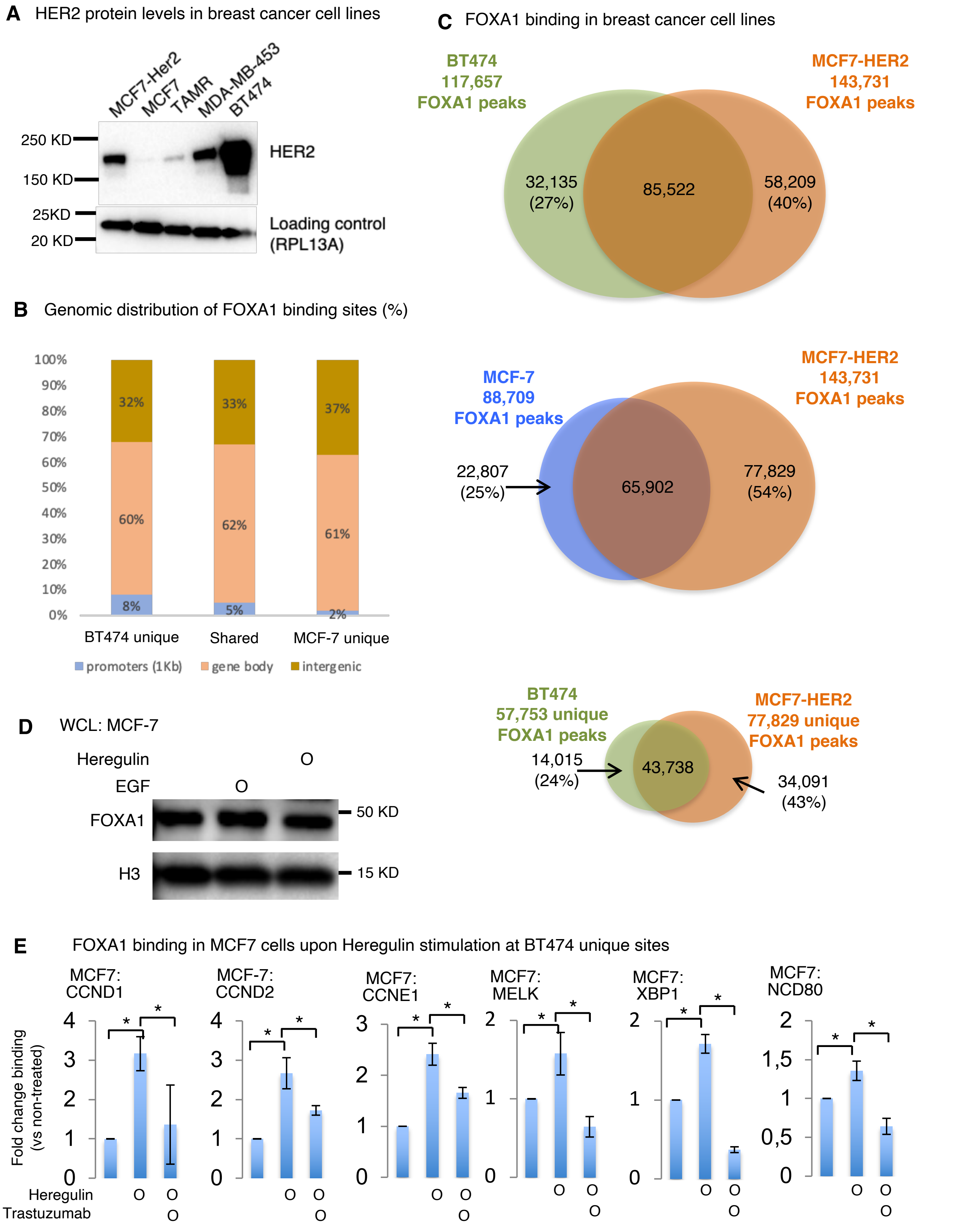

*: Heregulin vs non-treated or Heregulin vs Heregulin+Transtuzumab (p<0.01)

Legend Figure Supplementary 1: (**A**) Western blot of FOXA1 and RPL13A (as a loading control) of protein extracts of breast cancer cells displaying different FOXA1 protein levels: MCF7-HER2, MCF-7, Tamoxifen resistant MCF-7 (TAM-R), MDA-MB-453 and BT474. (**B**) Genomic distributions at each of the FOXA1 sites identified at MCF-7 or BT474 cells using as a reference MCF-7 unique peaks, MCF-7 and BT474 shared peaks and BT474 unique peaks. The genomic distribution has been determined by using Homer. (**C**) Venn Diagram showing the overlap in FOXA1 chromatin interactions (ChIP-sequencing) between BT474 and MCF7-HER2 cells (top panel) and between MCF-7 and MCF7-HER2 cells (intermediate panel) and unique FOXA1 sites of BT474 cells (57,753 peaks) with MCF7-HER2 (77,829 peaks) (lower panel). (**D**) Western blot with immunoblot for FOXA1 or H3 (as loading control) of total protein extracts of MCF-7 cells control treated (vehicle), treated with EGF or heregulin for 1h. (E) ChIP and QPCR of MCF-7 cells control treated, treated with heregulin (1h) or with heregulin with trastuzumab (1h).

Figure Supplementary 2

A Transcription Factors (TF) identified at FOXA1 chromatin regions based on motif analysis

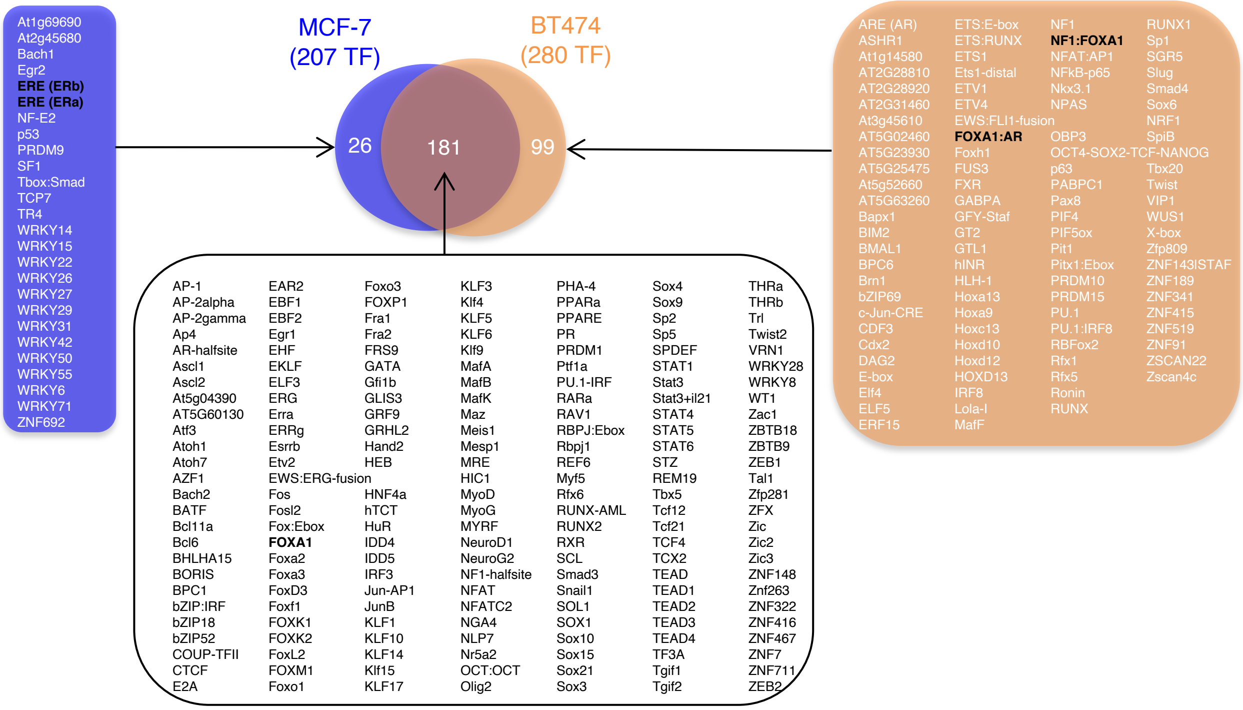

B Motif analysis at FOXA1 chromatin regions

FOXA1 peaks	FOXA1 (FKH) motif	ERE motif	AP2γ motif	PBX1 motif
MCF7-specific	78% (24%) p-value = 1e-6171	4% (1%) p-value = 1e-174	6% (3%) p-value 1e-115	Undetectable motif with p value lower than 1e-100
MCF7-BT474 shared	79% (25%) p-value = 1e-13461	5,5% (2%) p-value = 1e-301	25% (7%) p-value 1e-2995	Undetectable motif with p value lower than 1e-100
BT474-specific	61% (17%) p-value = 1e-8703	Undetectable motif with p value lower than 1e-100	27% (6%) p-value 1e-4045	Undetectable motif with p value lower than 1e-100

Legend Figure Supplementary 2. Motif analysis within FOXA1 differentially regions regulated by HER2. (A) Comparative motif analysis between the FOXA1 specific chromatin interactions at MCF-7 and at BT474 cells. The analysis revealed that a total of 207 motifs corresponding to transcription factors (TF) were identified at FOXA1 chromatin regions of MCF-7 cells and a total of 280 TF were identified at FOXA1 chromatin regions of BT474 cells. A comparative analysis (Venn Diagram) was performed with the TF identified and a substantial overlap of TF was identified. Several forkhead TF, including FOXA1, were commonly identified at both lists, suggesting that FOXA1 is likely able to bind many of these chromatin regions regardless of HER2 status. We identified 26 transcription factors at the list of TF of FOXA1 unique sites at MCF-7 cells. Among the TF, we identified ER alpha and ER beta, confirming our previous motif analysis. We also observed that at the list of TF of FOXA1 unique sites of BT474, FOXA1 sites were enriched with dimer motifs recognized by forkhead motifs with nuclear receptor AR or with transcription factor NF1. (B) FOXA (FKH motif), ER (ERE motif), PBX1 and AP2γ frequency motifs were determined by using HOMER.

Figure Supplementary 3

A Inhibition of HER2/HER3 signaling increased FOXA1 acetylation in MCF-7 HER2 high cell line

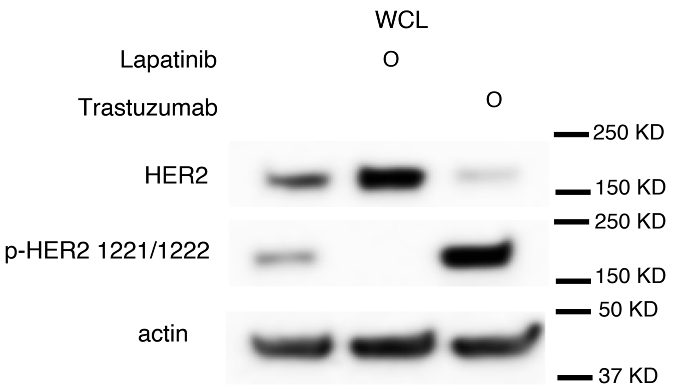

B Inhibition of HER2/HER3 signaling increased FOXA1 acetylation in BT474 cell line

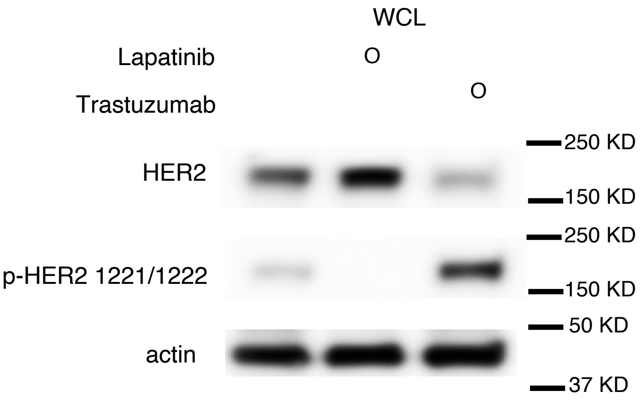

C Biochemical structure of FOXA1 K240T mutant with DNA

FOXA1 Tre-240

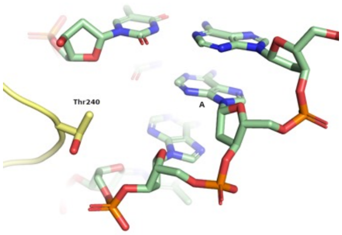

D Expression of estrogen genes in MCF-7 cells treated with Fulvestrant and Heregulin

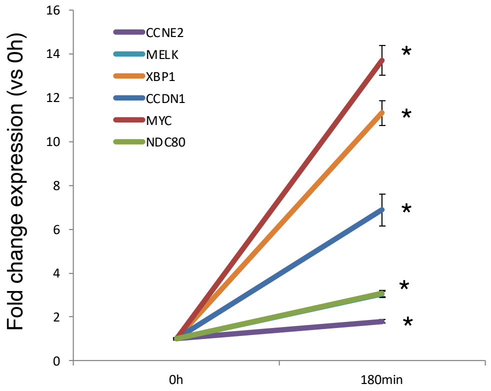

*: 180min vs 0min (p<0.01)

Legend Figure Supplementary 3. Related to figure 3. MCF7-HER2 (**A**) and BT474 (**B**) cell lines were control treated (vehicle) and compared to cells treated with anti-HER2 drugs Trastuzumab or Lapatinib. In both cell lines the HER2 and phosphor-HER2 protein levels were determined by western blot from whole cell lysates (WCL). Actin was used as a loading control and non-phosphorylated or phosphorylated (Tyr 1221/1222) of HER2 were determined by western blot. (**C**) Forkhead domain involved in DNA binding (PDB ID 7VOX) of mutated FOXA1 K240T. (**D**) Real-time PCR of genes induced by heregulin in fulvestrant treated MCF-7 cells relative mRNA level is normalized against TPB, then to the average of 3h of treatment with heregulin is normalized vs 0h of treatment. The data is represented as the mean of independent replicates ± s.d. T-test (two tails) (n = 3, *, $p<0.05$).

Figure Supplementary 4

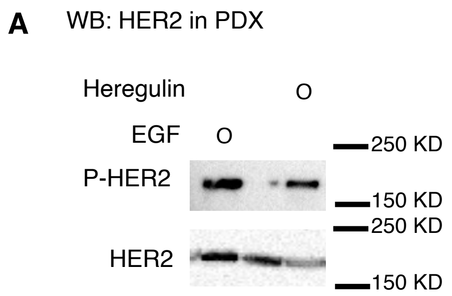

Legend Figure Supplementary 4. Related to figure 4. (**A**) Animals were control treated (vehicle) or treated with heregulin or EGF and protein from PDX tumors was isolated HER2 and phosphor-HER2 protein levels were determined by western blot from whole tumor lysates (WCL). Actin was used as a loading control and non-phosphorylated or phosphorylated (Tyr 1221/1222) of HER2 were determined by western blot.

Figure Supplementary 5

A ER levels in breast cancer cells

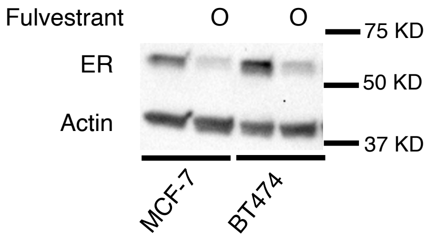

B Cell growth: breast cancer cells

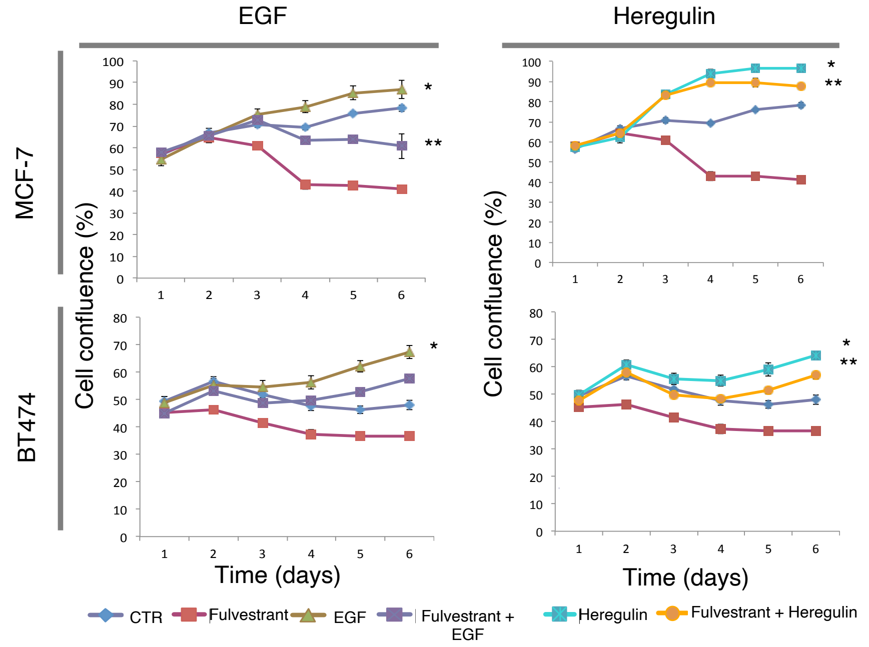

*: Control vs growth factor (p<0.001)
**: Control vs Fulvestrant + growth factor (p<0.001)

C P-Protein levels in MCF-7 cells

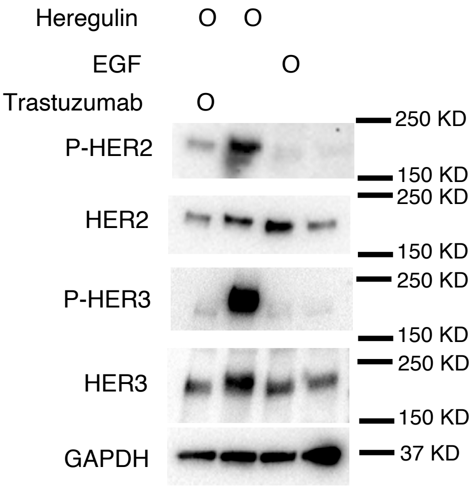

D Cell growth induced by growth factors upon siFOXA1

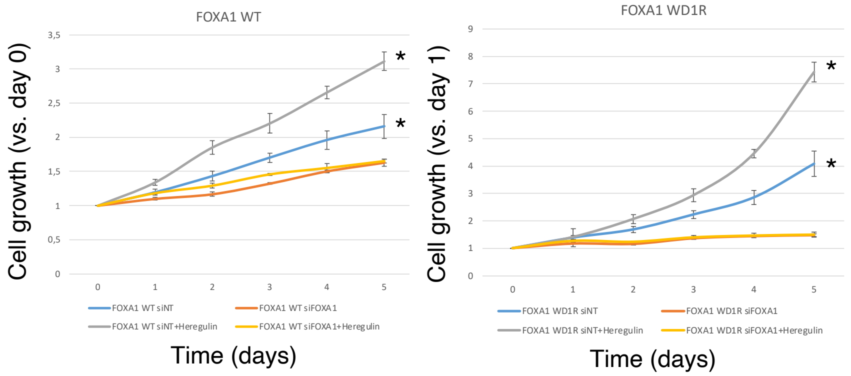

*: FOXA1 WT or FOXA1 WD1R: siFOXA1 vs siNT (p<0.01)

*: FOXA1 WT or FOXA1 WD1R: siFOXA1+Heregulin vs siNT+Heregulin (p<0.01)

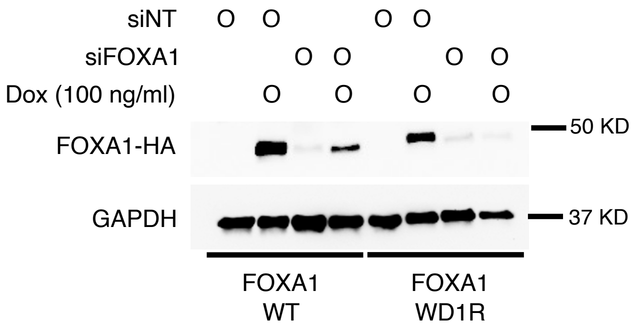

E Cell growth mediated by FOXA1 and Heregulin

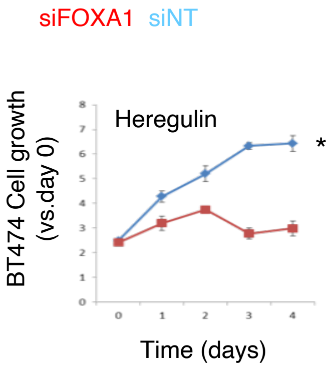

*: siFOXA1 vs siNT (p<0.01)

Legend Figure Supplementary 5. Complementary information of figure 4. (**A**) Western blot confirming reduced levels of ER after treatment with fulvestrant in MCF-7 cells. (**B**) Cell proliferation of MCF-7 or BT474 with EGF or Heregulin in the presence or absence of fulvestrant. (**C**) Western blot analyses of HER2non-phosphorylated or phosphorylated, HER3 non-phosphorylated or phosphorylated and actin (as a loading control) in MCF-7 cells control treated (vehicle), EGF, heregulin or heregulin plus trastuzumab. (**D**) siControl or siFOXA1 transfected MCF-7 cells stably expressing FOXA1 WT (left panel) or FOXA1 WD1R mutant (right panel). Cell growth was measured when cells were stimulated with heregulin or vehicle. The data are the mean of six independent replicates ± s.d. T-test (two tails) (n = 3, *, p<0.05). (**E**) siControl or siFOXA1 transfected BT474 cells. (**D**) Upper panel: cell growth was measured when cells were stimulated with heregulin and compared siNT vs. siFOXA1. The data are the mean of six independent replicates ± s.d. T-test (two tails) (n = 3, *, p<0.05). Lower panel: Western blotting of MCF-7-FOXA1-WT or MCF-7-FOXA1-WD1R mutant were transiently transfected with non-targeting siRNA (siNT), siRNAs targeting FOXA1 (siFOXA1). Cells were treated with Doxycycline (100 ng/ml) and protein extracts from MCF-7 transfected was collected and HA (tag of FOXA1 stably transfected cells) was detected by Western blotting. GAPDH was used as a loading control.

Figure Supplementary 6

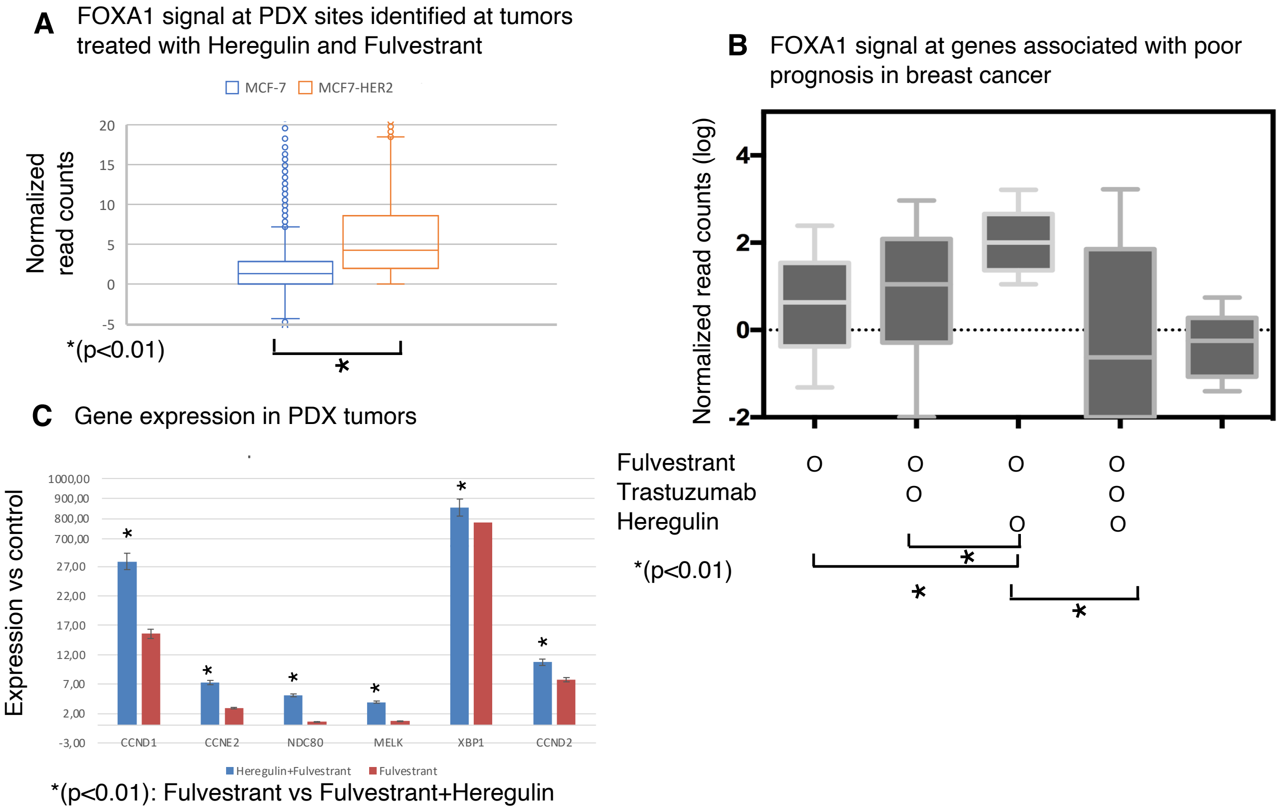

Legend Figure Supplementary 6. Additional complementary information of figure 4. (**A**) Box-plot indicating the average binding intensity of FOXA1 normalized reads (in a logarithmical scale) for peaks from 19,353 FOXA1 peaks identified in fulvestrant and heregulin treated animals. The FOXA1 binding intensity from ChIP-seq MCF-7 and MCF7-HER2. Wilcoxon rank-sum test was used to test any statistical difference between samples (5% FDR with two tails). (**B**) Box-plot indicating the average binding intensity of FOXA1 normalized reads (in a logarithmical scale) for peaks from FOXA1 peaks identified in fulvestrant and heregulin treated animals associated to genes expressed in patients with worse prognosis in breast cancer. The FOXA1 binding intensity from ChIP-seq PDX tumors of animals with different treatments. Wilcoxon rank-sum test was used to test any statistical difference between samples (5% FDR with two tails). (**C**) Real-time PCR of genes expressed in worse prognosis patients from PDX tumors of animals treated with control, fulvestrant or heregulin with fulvestrant. Relative mRNA level is normalized against TPB, then to the average of treatments is normalized vs control. The data is represented as the mean of independent replicates ± s.d. T-test (two tails) (n = 3, *, $p<0.05$).

Figure Supplementary 7

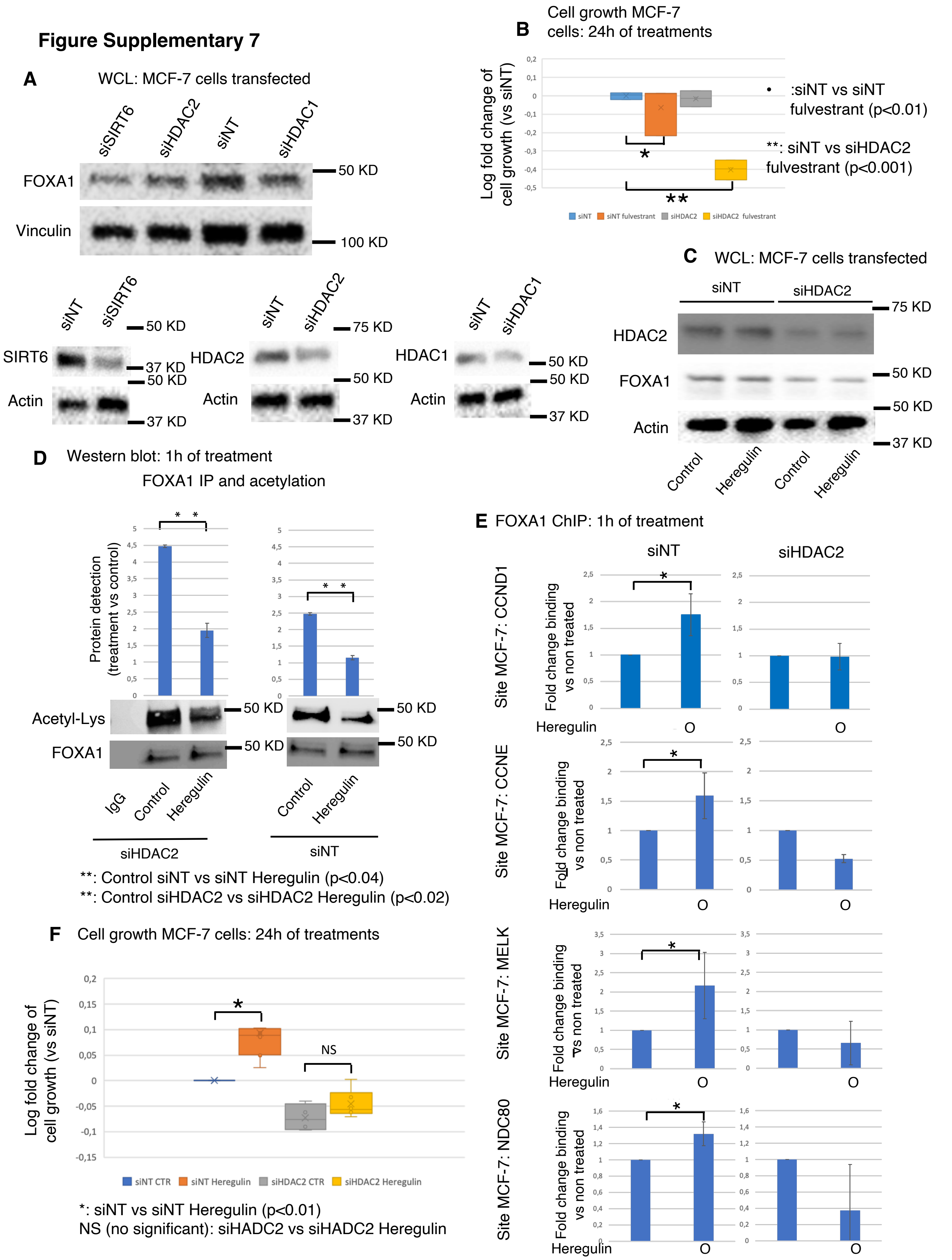

Figure Supplementary 7. Complementary information of figure 5. (**A**) Western blotting of MCF-7- cells transiently transfected with non-targeting siRNA (siNT), siRNAs targeting HDAC1 (siHDAC1) or HDAC2 (siHDAC2) or SIRT6 (siSIRT6). Protein extracts from MCF-7 transfected was collected and FOXA1 (upper panel) and SIRT6, HDAC2 and HDAC1 (lower panel) were detected by Western blotting. Actin was used as a loading control. (**B**) Cell growth of MCF7 cells transfected with siNT or siHDAC2 oligonucleotides. The plot aims at comparing the growth with non-treated or combined treatment of fulvestrant in siNT (control) transfected cells. Furthermore, the plot aims at comparing the growth with non-treated or combined treatment of fulvestrant in siHDAC2 transfected cells. (**C**) Western blotting of MCF-7- cells transiently transfected with non-targeting siRNA (siNT), siRNAs targeting HDAC2 (siHDAC2). Protein extracts from MCF-7 transfected was collected and HDAC2 and FOXA1 were detected by Western blotting. Actin was used as a loading control. Data is represented as logarithmic fold change of cell growth at 24h of treatment vs 0h. The data are the mean of independent replicates ± s.d. T-test (two tails) was applied to determine statistical differences. (**D**) FOXA1 acetylation from immunoprecipitated FOXA1 protein was determined by western blot in MCF7 transiently transfected with siHDAC2 or siNT. The plot aims to compare the FOXA1 binding to chromatin of transfected cells when cells were treated with heregulin for 1h. (**E**) MCF7 transiently transfected with siHDAC2 or siNT. The plot aims to compare the FOXA1 binding to chromatin of transfected cells when cells were treated with heregulin for 1h. Data is represented as fold change of FOXA1 binding of heregulin treated cells compared to control treated cells. The data are the mean of independent replicates ± s.d. T-test (two tails) was applied to determine statistical differences. (**F**) Cell growth of MCF7 transiently transfected with siHDAC2 or siNT. The plot aims to compare the growth of transfected cells when cells were treated with heregulin for 1h. Data is represented as logarithmic fold change of cell growth at 24h of treatment vs 0h. The data are the mean of independent replicates ± s.d. T-test (two tails) was applied to determine statistical differences.
